# Ancient mitogenomes from Pre-Pottery Neolithic Central Anatolia and the effects of a Late Neolithic bottleneck in sheep (*Ovis aries*)

**DOI:** 10.1126/sciadv.adj0954

**Published:** 2024-04-12

**Authors:** Edson Sandoval-Castellanos, Andrew J. Hare, Audrey T. Lin, Evangelos A. Dimopoulos, Kevin G. Daly, Sheila Geiger, Victoria E. Mullin, Ingrid Wiechmann, Valeria Mattiangeli, Gesine Lühken, Natalia A. Zinovieva, Petar Zidarov, Canan Çakırlar, Simon Stoddart, David Orton, Jelena Bulatović, Marjan Mashkour, Eberhard W. Sauer, Liora Kolska Horwitz, Barbara Horejs, Levent Atici, Vecihi Özkaya, Jacqui Mullville, Michael Parker Pearson, Ingrid Mainland, Nick Card, Lisa Brown, Niall Sharples, David Griffiths, David Allen, Benjamin Arbuckle, Jordan T. Abell, Güneş Duru, Susan M. Mentzer, Natalie D. Munro, Melis Uzdurum, Sevil Gülçur, Hijlke Buitenhuis, Elena Gladyr, Mary C. Stiner, Nadja Pöllath, Mihriban Özbaşaran, Stefan Krebs, Joachim Burger, Laurent Frantz, Ivica Medugorac, Daniel G. Bradley, Joris Peters

**Affiliations:** ^1^ Population Genomics Group, Department of Veterinary Sciences, LMU Munich, 82152 Martinsried, Germany.; ^2^ Institute of Palaeoanatomy, Domestication Research and the History of Veterinary Medicine, LMU Munich, 80539 Munich, Germany.; ^3^ Smurfit Institute of Genetics, Trinity College Dublin, Dublin D02 PN40, Ireland.; ^4^ The Palaeogenomics and Bio-archaeology Research Network, Research Laboratory for Archaeology and History of Art, University of Oxford, Oxford, UK.; ^5^ Department of Anthropology, National Museum of Natural History, Smithsonian Institution, Washington, DC, 20560 USA.; ^6^ Department of Veterinary Medicine, University of Cambridge, Cambridge, UK.; ^7^ School of Agriculture and Food Science, University College Dublin, Dublin, Ireland.; ^8^ Institute of Animal Breeding and Genetics, Justus Liebig University of Gießen, Ludwigstr. 21, 35390 Gießen, Germany.; ^9^ L.K. Ernst Federal Research Centre for Animal Husbandry, Dubrovitsy, Podolsk, Moscow Region, Russia.; ^10^ Institute of Prehistory, Early History and Medieval Archaeology, Tübingen University, Tübingen, Germany.; ^11^ Institute of Archaeology, University of Groningen, 9712 ER Groningen, Netherlands.; ^12^ Magdalene College, University of Cambridge, Cambridge CB3 0AG, UK.; ^13^ BioArCh, Department of Archaeology, University of York, York YO10 5NG, UK.; ^14^ Department of Historical Studies, University of Gothenburg, BOX 200, 40530 Gothenburg, Sweden.; ^15^ Unité Archéozoologie, Archéobotanique, Sociétés Pratiques et Environnements (AASPE), CNRS, Muséum National d’Histoire Naturelle, 75020 Paris, France.; ^16^ School of History, Classics and Archaeology, University of Edinburgh, Old Medical School, Teviot Place, Edinburgh EH8 9AG, UK.; ^17^ National Natural History Collections, Faculty of Life Sciences, The Hebrew University, Jerusalem, Israel.; ^18^ OeAI, Austrian Academy of Sciences and HEAS, University of Vienna, Vienna, Austria.; ^19^ Department of Anthropology, University of Nevada, Las Vegas, NV 89154, USA.; ^20^ Department of Archaeology, Dicle University, Diyarbakir, Türkiye.; ^21^ School of History, Archaeology and Religion, Cardiff University, Cardiff CF10 3EU, UK.; ^22^ Institute of Archaeology, University College London, London, UK.; ^23^ The University of the Highlands and Islands Orkney, Kirkwall, UK.; ^24^ Wiltshire Museum, Devizes SN10 1NS, UK.; ^25^ University of Oxford, OUDCE, Rewley House, Oxford OX1 2JA, UK.; ^26^ Hampshire Cultural Trust, Chilcomb House, Winchester, SO23 8RB, UK.; ^27^ Department of Anthropology, University of North Carolina at Chapel Hill, Chapel Hill, NC 27599, USA.; ^28^ Department of Geosciences, University of Arizona, Tucson, AZ 85721, USA.; ^29^ Department of Archaeology, Mimar Sinan Fine Arts University, 34381 Şişli/İstanbul, Türkiye.; ^30^ Senckenberg Centre for Human Evolution and Palaeoenvironment, Institute for Archaeological Sciences, Department of Geosciences, Tübingen University, 72074 Tübingen, Germany.; ^31^ Department of Anthropology, University of Connecticut, Storrs, CT 06269, USA.; ^32^ Department of Archaeology, Ondokuz Mayıs University, 55270 Atakum/Samsun, Türkiye.; ^33^ Prehistory Department, Faculty of Letters, Istanbul University, 34134 Istanbul, Türkiye.; ^34^ Archeosupport, 9712 LN Groningen, Netherlands.; ^35^ School of Anthropology, University of Arizona, Tucson, AZ 85721, USA.; ^36^ Bavarian Natural History Collections, State Collection of Palaeoanatomy Munich, 80333 Munich, Germany.; ^37^ ArchaeoBioCenter, LMU Munich, 80539 Munich, Germany.; ^38^ Laboratory for Functional Genome Analysis (LAFUGA), Gene Center, LMU Munich, Feodor-Lynen-Straße 25, 81377 Munich, Germany.; ^39^ Institute of Organismic and Molecular Evolution (iomE), Johannes Gutenberg University Mainz, 55128 Mainz, Germany.; ^40^ Palaeogenomics Group, Institute of Palaeoanatomy, Domestication Research and the History of Veterinary Medicine, LMU Munich, 80539 Munich, Germany.; ^41^ School of Biological and Behavioural Sciences, Queen Mary University of London, London, UK.

## Abstract

Occupied between ~10,300 and 9300 years ago, the Pre-Pottery Neolithic site of Aşıklı Höyük in Central Anatolia went through early phases of sheep domestication. Analysis of 629 mitochondrial genomes from this and numerous sites in Anatolia, southwest Asia, Europe, and Africa produced a phylogenetic tree with excessive coalescences (nodes) around the Neolithic, a potential signature of a domestication bottleneck. This is consistent with archeological evidence of sheep management at Aşıklı Höyük which transitioned from residential stabling to open pasturing over a millennium of site occupation. However, unexpectedly, we detected high genetic diversity throughout Aşıklı Höyük’s occupation rather than a bottleneck. Instead, we detected a tenfold demographic bottleneck later in the Neolithic, which caused the fixation of mitochondrial haplogroup *B* in southwestern Anatolia. The mitochondrial genetic makeup that emerged was carried from the core region of early Neolithic sheep management into Europe and dominates the matrilineal diversity of both its ancient and the billion-strong modern sheep populations.

## Teaser

Analyses of 629 sheep mitogenomes detected no early domestication bottleneck but a Late Neolithic bottleneck in Anatolia.

## INTRODUCTION

The establishment of Neolithic sedentary societies in southwest Asia was associated with the development of farming practices between 10 thousand and 12 thousand calibrated years before the present (ka cal BP). Those practices included the cultivation of cereals and legumes and the management of sheep, goats, cattle, and pigs, which ultimately resulted in their domestication (*1*–*9*). Crop-livestock subsistence strategies started gaining ground around 10.5 ka cal BP in the northern “Fertile Crescent,” and by 9.5 ka cal BP, this mode of subsistence had replaced the foraging lifestyle in parts of southwest Asia and Cyprus (*2*, *10*–*14*). Archaeobotanical and zooarcheological analyses showed that these millennia-long practices of crop cultivation and ungulate management led to phenotypic changes in both plants and animals (*2*–*4*, *15*–*18*). To understand these changes, it is often necessary to integrate the evidence of multiple sites and millennia (*3*, *19*). However, only a few Pre-Pottery Neolithic sites preserved a sufficiently long occupation history and representative faunal assemblages to track morphological, biometric, and demographic changes related to early livestock management at a single location. The list of such exceptional sites in Anatolia includes Çayönü, Cafer Höyük, and Nevalı Çori in Southeastern Anatolia and Aşıklı Höyük in Central Anatolia (*1*, *3*, *20*).

Aşıklı Höyük is situated on the bank of the Melendiz River (*21*, *22*). Here, large numbers of caprine bones (i.e., sheep and goats) have been excavated from occupational phases spanning over a thousand years, between ~10.3 and 9.3 ka cal BP. The importance of small livestock management at the site was such that, during the thousand-years occupation, the composition of animal remains identified as sheep and goat increased from ~50% to 87% (table S1) (*8*, *23*, *24*). Analyses of this extraordinary assemblage provided a unique glimpse into early strategies of sheep management. This includes mortality curves that are indicative of the culling of juvenile males, which in turn reflects exploitation for meat (*23*), and spatial patterns of skeletal distribution that imply that slaughtering took place near the living quarters (*18*). Further analyses of intra-articular joint pathologies suggested restricted mobility close to the village, including residential stabling (*25*), which led to the accumulation of dung and urine salts in the sediments (*26*, *27*).

By 9.7 ka cal BP, however, sheep management strategies apparently shifted toward extensive herding. Evidence for this includes a decrease in urine salt and dung concentrations in residential areas (*26*, *27*), an increase in carcass size (table S2), an improvement in joint health implying greater mobility (table S3) (*25*), and shifts in phytolith and stable isotope profiles that imply more extensive grazing (*18*, *28*, *29*). Together, the evidence obtained at Aşıklı Höyük demonstrates that sheep management in early Neolithic communities was a dynamic process of learning by doing (*8*, *18*, *25*).

Although management strategies at Aşıklı Höyük likely affected the phenotype of sheep populations, it is not clear whether they initiated evolutionary changes that ultimately led to the strong genetic differentiation between wild and domestic populations that we observe today. A common assumption is that capture and spatial isolation of a subset of a wild population induced a “domestication bottleneck,” provoking the general reduction of genetic diversity evident in modern domestic sheep populations (*30*).

Here, to address whether the initial management of sheep at Aşıklı Höyük caused shifts in their genetic makeup, we analyzed 629 whole mitogenomes sourced from 15 countries, including 62 from Aşıklı Höyük, spanning a period of over 10,000 years. This allowed us to infer the mitochondrial phylogeography and maternal demographic history of Anatolian and European sheep, and the contribution of the Aşıklı Höyük community to the formation of the Neolithic package dispersing across north and southwestern Anatolia between 10.0 ka and 8.0 ka cal BP, and subsequently into Europe.

## RESULTS

### Mitogenome dataset

We investigated 171 ancient samples from 7 sites in Anatolia (*n* = 102), 8 localities in the Levant and Caucasus (*n* = 24), and 18 localities in Europe (*n* = 45). Radiocarbon dating of associated charcoal and plant annuals placed our ancient samples in the Neolithic, Chalcolithic, Bronze Age, Iron Age, Middle Ages, and post-Medieval periods. In addition, we used data mining to retrieve two historic sequences (York, UK, 0.247 ka cal BP—the year 1776 AD—) from the Sequence Read Archive (see Materials and Methods).

**Fig. 1.**
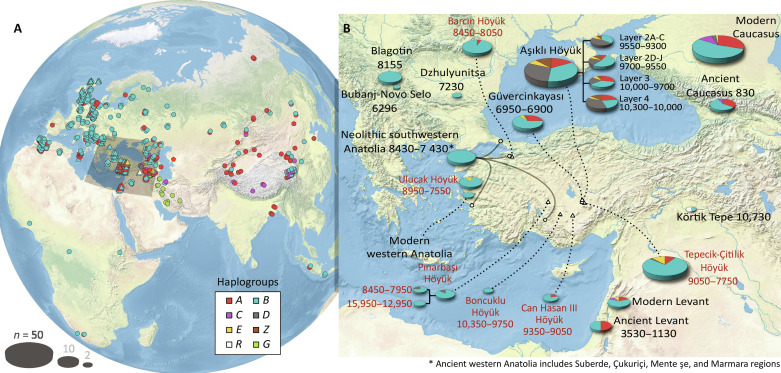
Temporal and geographical distributions of haplogroup frequencies. (**A**) Worldwide (jittered) location of all 629 samples analyzed in this study. (**B**) The map shows details of Anatolia and surrounding regions with haplogroup frequencies by archeological site, except for Neolithic southwestern Anatolia, where the grouping is at the regional scale (notice the tentacles out of the pie chart indicating the included locations, named below the map). Text in red indicates data reported in previous studies (*42*, *43*), wherein haplogroups *A* to *E* were assigned using five diagnostic single-nucleotide polymorphism (SNP) markers of a 144–base pair (bp) control region fragment. Pie chart areas are proportional to their sample sizes (key, bottom left). The numbers accompanying location names refer to their estimated age range (calibrated years before the present) based on the site’s stratigraphy, material culture, and radiocarbon dating of appropriate materials including charcoal and annual plants.

For modern sheep, our study generated 147 mitogenomes and retrieved 164 mitogenomes by data mining from the Sequence Read Archive. Furthermore, we sourced 136 mitogenomes from published studies (*30*–*40*) and 9 directly from GenBank (acc. numbers in table S5). The complete dataset, which included mitogenomes from 24 modern mouflons (*Ovis gmelini*) from Iran, and 1 urial (*Ovis vignei*), encompasses 173 ancient and 456 modern sheep mitogenomes for a total of 629 individuals (Fig. 1 and tables S4 and S5).

### Haplogroups distribution and diversity/neutrality indexes

**Fig. 2.**
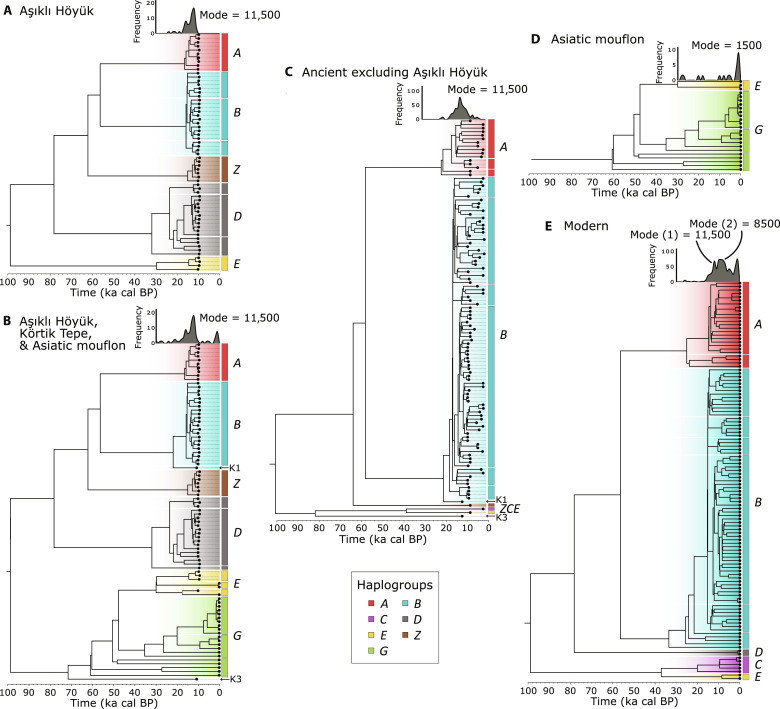
Subtrees from the phylogenetic inference. This figure shows five subtrees of the full phylogenetic tree (figs. S3 to S7) to facilitate interpretation: (**A**) subtree of all 62 Aşıklı Höyük samples; (**B**) subtree of samples from Aşıklı Höyük, Körtik Tepe, and Asiatic mouflons (*O. gmelini*); (**C**) subtree of all ancient samples except those from Aşıklı Höyük; (**D**) subtree of Asiatic mouflons; and (**E**) subtree from present day samples (randomly subsampled with proportional representation of haplogroups). K1 and K3 indicate the mouflons from Körtik Tepe. Graphs above the trees correspond to smoothed histograms of inferred coalescent times (nodes). Their horizontal scale (times) is the same as that of the trees. The tree of modern domestic sheep exhibits a first peak of coalescences at 11.5 ka cal BP and a second peak in the tree of modern sheep around 8.5 ka cal BP. These are absent in the wild sheep sample.

In our dataset, we identified previously reported domestic sheep haplogroups *A* to *E*, as well as an unknown haplogroup that we labeled as *Z* (Fig. 2). In addition, we labeled modern mouflon (*O. gmelini*) haplogroups collectively as *G*. In modern sheep, the major mitochondrial haplogroups show a marked structure. In western herds populating Europe and North Africa, haplogroup *B* predominated (frequencies = 0.87 and 0.95, respectively). However, haplogroup *B* appeared less prominently in the Caucasus (frequency = 0.54) and in the Levant (frequency = 0.63) and exhibited a lower frequency than *A* in eastern Asia [frequency (*B*) = 0.36; frequency (*A*) = 0.5], where it coexisted with haplogroups *C*, *D*, and *E* that were less common and mostly endemic (Fig. 1 and Table 1).

**Table 1.** Mitogenomic haplogroup frequencies. Individual samples match the sample codes in table S5. Temporal tests of haplogroup frequencies used the groups and frequencies shown here, except for modern Anatolia, Körtik Tepe, and Asiatic mouflon, that were not subject to such analyses due to sample size. The table shows the absolute frequencies of mitochondrial haplogroups *A*, *B*, *C*, *D*, *E*, *Z*, *G*, and unassigned (*R*).

Sample code	Sample origin	Mean age (ka cal BP)	Haplogroup								
			*A*	*B*	*C*	*D*	*E*	*Z*	*R*	*G*	Total
aEU	Ancient Europe	4.11	1	46							47
aSWAN	Neolithic southwestern Anatolia	7.72		18							18
aCC	Ancient Caucasus	0.83	5	8	1						14
aLEV	Ancient Levant	2.72	5	5							10
aAH	Asikli Höyük (all layers)	9.83	10	22		19	4	7			62*
aAH1	Asikli Höyük levels 2A-C	9.43		4		5	1	1			11
aAH2	Asikli Höyük levels 2D-J	9.63	1	8		3	1	2			15
aAH3	Asikli Höyük level 3	9.85	4	5		3	1	1			14
aAH4	Asikli Höyük level 4	10.15	5	5		8	1	3			22
aGK	Chalcolithic Güvercinkayası	6.93	5	13			1	1			20
aKT	Körtik Tepe	10.73		1					1		2
mAFR	Modern Africa	0.0	3	89	2						94
mEU	Modern Europe	0.0	23	154							177
mWAN	Modern western Anatolia	0.0		3							3
mCC	Modern Caucasus	0.0	18	32	6	2	1				59
mLEV	Modern Levant	0.0	2	7	1		1				11
mEASIA	Modern eastern Asia	0.0	44	32	11						87
mOO	Asiatic mouflon	0.0					3			21	24
OUT	Urial	0.0							1		1
Total											629

*Includes all four layers below.

**Fig. 3.**
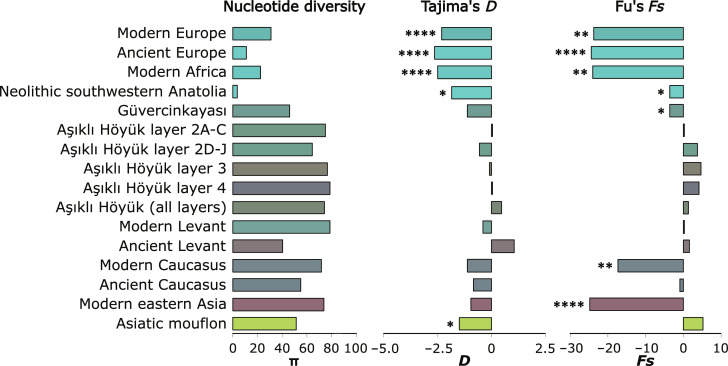
Population genetics statistics. Estimations of nucleotide diversity, Tajima’s *D*, and Fu’s *Fs*. The asterisks show the significance level of the associated statistical test on the statistics (* = 0.01 to 0.05, ** = 0.001 to 0.01, *** = 0.0001 to 0.001, and **** = 0.00001 to 0.0001). Significant values of Tajima’s and Fu’s neutrality tests could indicate past demographic bottlenecks. The bars’ colors correspond to the sample frequency-weighted average of the haplogroup’s colors shown in Figs. 1 and 2. These statistics suggest a bottleneck in the ancestry line of the European populations and possibly Neolithic southwestern Anatolia, while the colors reflect the haplogroup differences between eastern and western samples (notice the gray tones of Aşıklı Höyük samples).

A spatial pattern compatible with the haplogroups distribution appeared in the nucleotide diversity as well as in the neutrality indexes and tests (Fig. 3). The samples from modern Africa, modern and ancient Europe, and Neolithic southwestern Anatolia, which had haplogroup *B* at high frequencies, show a depleted nucleotide diversity (0.0004 to 0.0022) and values of Tajima’s *D* and Fu’s *Fs* statistics that were negative, large, and significant (Tajima’s *D* = −2.63 to −1.84; Fu’s *Fs* = −23.8 to −to 3.65; *P* < 0.00001 to 0.015 and 0.002 0.048, respectively). In contrast, all post-Neolithic samples (including modern) from Anatolia, Caucasus, Levant, and eastern Asia displayed markedly higher nucleotide diversities (0.0033 to 0.0053), higher nonsignificant Tajima’s *D* values (−1.121 to 1.048; *P* = 0.113 to 0.894), and low and significant Fu’s *Fs* values (−24.80 to −3.653; *P* ≤ 0.0001 to 0.712). More notably, Aşıklı Höyük displayed the highest nucleotide diversity (0.0053), a positive nonsignificant Tajima’s *D* value (0.453; *P* = 0.762), and a positive nonsignificant value of Fu’s *Fs* (1.283; *P* = 0.722). In addition, the subsequent levels of Aşıklı Höyük did not show significant differences among them for these statistics and tests (Fig. 3).

### Tests of temporal change in haplogroup frequencies

When we tested the temporal changes in haplogroup frequencies, we found, in general, significant differences between, but not within, groups. The western group included Africa, Europe (modern and ancient), and Neolithic southwestern Anatolia, while the eastern group included Neolithic Aşıklı Höyük, Chalcolithic Güvercinkayası, Caucasus, Levant, and eastern Asia. This pattern was true when the tests were performed with effective population sizes (*N_e_*) between 10^4^ and 10^6^ (we refrained from writing *P* values here because of the numerous combinations of *N_e_* used in these analyses; see table S6 for the exact values and fig. S2 for a graphical summary of these results). The whole pattern of significance makes sense of the patterns of diversity/neutrality indexes and leads to an important observation that the divergence between eastern and western groups seems to have originated between central and southwestern Anatolia, during the Neolithic (fig. S2).

In addition, the temporal tests detected no significant changes in haplogroup frequencies across Aşıklı Höyük’s occupation layers (*P* = 0.28 to 0.97; fig. S2 and table S6).

### Phylogenetic analyses and demographic inference on coalescent times

Our phylogenetic analysis recovered clades corresponding to the major domestic sheep haplogroups *A* to *E*, as well as an unknown haplogroup that we labeled as *Z*, in addition to the modern mouflon haplogroups (*O. gmelini*) collectively labeled as *G* (Fig. 2 and figs. S3 to S7). Notably, within haplogroups *A* to *E* and *Z*, we found a high density of coalescent events (nodes) between 12.0 ka and 7.6 ka cal BP (50% high-density interval), peaking around 11.5 ka cal BP.

**Fig. 4.**
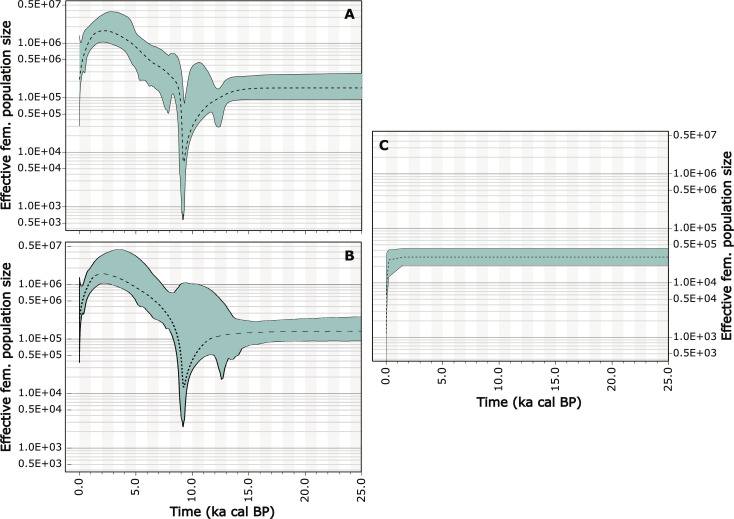
Extended Bayesian skyline plots. The charts show demographic histories inferred with the samples of (**A**) modern and ancient Europe, the Neolithic sites of Aşıklı Höyük, Körtik Tepe, and southwestern Anatolian (including Suberde, Çukuriçi, Menteşe, and Marmara regions); (**B**) the same samples minus Neolithic southwestern Anatolian sites; and (**C**) modern Asiatic mouflon, plus two Körtik Tepe and one Aşıklı Höyük samples for time calibration. The dotted lines indicate the median values, while the gray areas represent the 95% highest probability density (HPD) regions. The plots in (A) and (B) show a strong population bottleneck peaking around 9.0 ka to 9.5 ka cal BP followed by a significant population expansion (consider the HPD areas). This may be a compendium of the domestication process, a demographic constriction postdating site occupation at Aşıklı Höyük, and a historical expansion of flocks of sheep into Europe.

We tested if this concentration of coalescences was an artifact of sampling by comparatively inferring the demographic histories of European-Anatolian samples and mouflon samples based solely on coalescent times (nodes ages) and sample ages. For this, we used an in-house developed method that takes advantage of the statistical technique known as Markov chain Monte Carlo (MCMC) (see text S8). Although at low resolution, the inferred demographic history of European-Anatolian sheep was compatible with the demographic history inferred by extended Bayesian skyline plots that were co-estimated with the phylogenetic reconstruction in the software BEAST. Both the MCMC procedure and the skyline plots suggest the occurrence of at least one major bottleneck that reached its peak between 8.0 ka and 10.0 ka cal BP, and a long-term demographic expansion thereafter, except for the last millennium that is characterized by a decline (Fig. 4 and fig. S8).

### Simulation-based inference by approximate Bayesian computation

After integrating the results of the different analyses, we constructed some hypotheses regarding the evolution of domestic sheep in Eurasia. To test them, we used the simulations-based statistical technique known as approximate Bayesian computation (ABC). The specific goal was to infer a temporal population structure relating to European and Anatolian sampled populations and to estimate key parameters such as *N_e_*. We split this inference into four phases with each phase targeting a simple inferential goal to prevent a number of technical problems such as high dimensionality and overparametrization. Figure 4A and figs. S9 to S11 show schematic representations of the models used in each phase.

The first phase of analyses by ABC targeted a demographic inference at Aşıklı Höyük. It showed that a model with two demographic phases (fig. S9B) was moderately better supported than a non-changing model (fig. S9A and table S9) (Bayes factor = 1.7 to 3.8). The parameter estimation of such a model supported a major increase in the sheep population throughout site occupation, although posterior distributions showed a large variance (fig. S12). Furthermore, an analysis of pseudo-observed datasets suggested that this analysis did not lack statistical power to detect large bottlenecks, as our ABC methodology could detect an eightfold bottleneck at Aşıklı Höyük around 90% of the time with very low stringency criteria (Bayes factor > 1.0) (fig. S13).

The second phase of ABC analyses expanded the focus of interest to the spatiotemporal structure of the sheep populations in Neolithic-Chalcolithic Anatolia. Specifically, the analyses aimed to pick the best among eight alternative scenarios (fig. S10). The best support was divided between two models. In both models, the populations of Neolithic southwestern Anatolia and Güvercinkayası were sisters, but in one model they descended directly from Aşıklı Höyük (model likelihood = 0.110 to 0.171), while in the other, they descended from an independent ancestral population (model likelihood = 0.159 to 0.188) (fig. S10 and table S11).

The third phase of ABC analyses aimed to select the best scenario of the ancestry of the European sheep population with respect to the populations of Neolithic-Chalcolithic Anatolia. In the best-supported scenario, Neolithic southwestern Anatolia was the ancestor of European sheep (Bayes factor = 1.4 to 342.0) (fig. S11 and table S13). This result was expected, though, considering the high prevalence of haplogroup *B* in both the European and Neolithic western Anatolian samples (frequencies = 0.979 and 0.870 in ancient and modern Europe; and = 1.0 in Neolithic western Anatolia) (see Fig. 1).

**Fig. 5.**
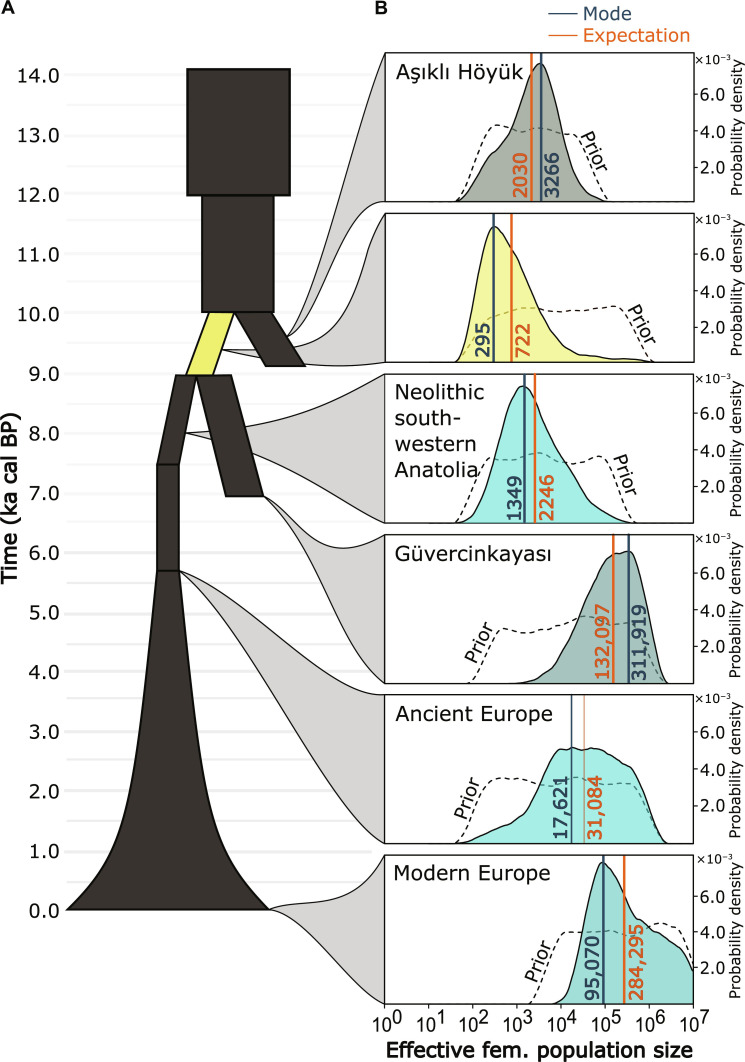
Modern and ancient *N_e_* values inferred by ABC. (**A**) Schematic representation of the model used in phase four of the simulation-based inference by ABC. Rectangles and trapezoids represent constant-size populations, while the inverted-funnel shape at the bottom represents a population with exponential growth (distributions corresponding to initial and final *N_e_* values); (**B**) posterior probability distributions (in color) of *N_e_*. The color matches the average haplogroup color of the corresponding samples (as in Fig. 3), except for the ancestral population (in yellow) of Neolithic southwestern Anatolia and Chalcolithic Güvercinkayası. Notice the small value of the central estimations of *N_e_* in such a population as compared to the sheep population of Aşıklı Höyük.

In the last phase of ABC analyses, we estimated with improved precision the *N*_*e*_ values of sampled and unsampled populations of a simulation design that was constructed upon the selected models of the second and third phases. In such a model, modern and ancient European sheep descend from herds populating Neolithic southwestern Anatolia, which in turn descended (together with Güvercinkayası) from a population that branched off Aşıklı Höyük’s stem population at a variable time between the Epipaleolithic and Aşıklı Höyük’s abandonment (see fig. S11). The punctual estimations for *N_e_* in Aşıklı Höyük were in the few thousand (mode = 2030), but the value estimated for the population that followed (i.e., the ancestor of Neolithic southwestern Anatolia and Güvercinkayası) dropped one order of magnitude (mode = 295). The estimations of *N_e_* for Neolithic western Anatolia were ~1.0 × 10^3^ to 2.0 × 10^3^, while for Güvercinkayası they were ~2.0 × 10^5^ to 3.0 × 10^5^. Expectedly, the population of sheep increased from the Anatolian Neolithic to the European Neolithic, and from there to the modern European sheep population (Fig. 5).

## DISCUSSION

### Haplogroups as potential markers of independent domestication

The phylogeographic structure that we observed in modern mitochondrial sheep haplogroups, where haplogroup *B* predominates in western populations and haplogroup *A* does so in eastern populations, is consistent with various reports [e.g., (*33*, *41*–*43*)]. Such structure has been explained by means of multiple domestication origins (*33*, *44*–*47*), the domestication of different wild populations or species (*33*), introgression (*41*), and lineage sorting-and-gene drift (*33*). Although multiple domestication origins are still being invoked to explain haplogroup structure (*44*–*47*), its popularity was most certainly influenced a few decades ago by the discovery of multiple domestication origins in pigs and cattle (*33*). However, sheep domestication centers outside southwest Asia have never been identified (*48*). Furthermore, goats, also believed to have had a unique domestication, exhibited a phylogeographic structure similar to the one in sheep before the post-Neolithic rise of the modern dominant haplogroup (*49*). In cattle, the two independently domesticated species (*Bos taurus* and *Bos indicus*) display a mitochondrial differentiation that is much higher than the observed among sheep populations, and their phylogeographic structure is heavily influenced by post-Neolithic introgression (*50*). Further criticisms of the multiple domestication hypothesis in sheep point out that the hypothesis equals haplogroups with populations while ignoring the diversity of the ancestral wild populations (*48*). In this regard, we show here that early Neolithic Aşıklı Höyük from Central Anatolia had haplogroups *A*, *B*, *D*, *E*, and unreported *Z* at high frequencies (0.161, 0.355, 0.306, 0.065, and 0.113, respectively) which is broadly consistent with what is reported in Neolithic sheep (see fig. S14) (*41*–*43*). These findings most definitely refute multiple domestication origins as an explanation for the modern distribution of these haplogroups.

Regarding another hypothesis, available evidence disregards the contribution of species other than Asiatic mouflon (*O. gmelini*) to the domestic sheep lineage, despite ample documentation of introgression among wild sheep species (*33*, *41*, *48*). It is possible, however, that domestic sheep descend from several mouflon populations with some degree of differentiation (see discussion below). Regarding lineage sorting and gene drift, we believe that they have some etiological value that is, however, conferred by further anthropogenic and demographic causes. When put in context, our evidence suggests that the near-fixation of haplogroup *A* in eastern Eurasian and haplogroup *B* in Europe and Africa emerged from Neolithic human-mediated migrations and demographic bottlenecks.

### Links between an increased density of coalescences and sheep domestication

In our phylogenetic reconstruction, we found a very high density of coalescent events (nodes) between 12.0 ka and 7.6 ka cal BP (peaking around 11.5 ka cal BP) within haplogroups *A* to *E* and *Z*. Two types of analyses, our in-house developed MCMC procedure and the extended Bayesian skyline plots, suggest that the observed pattern of concentrated coalescences is not due to a sampling bias but rather associated with demographic constriction. Furthermore, Asiatic mouflons did not present a concentration of coalescences nor bottlenecks in the demographic histories inferred by the skyline plots or our MCMC procedure (Fig. 3 and fig. S8), suggesting that the demographic process responsible for it may well be unique to managed rather than to wild sheep. One more clue regarding the origin of the concentration of coalescences is the fact that it coincides with the early phases of sheep management in southwest Asia (*2*, *19*). A similar coalescence pattern (between 11.0 ka and 9.5 ka cal BP) also appeared in ancient domestic goats (*51*) and is consistent with an inferred Neolithic bottleneck in cattle (*52*). Therefore, it seems that the concentration of coalescent events in sheep relates to founder events associated with the species’ early management potentially because of multiple capture events and breeding in captivity, briefly a domestication bottleneck.

That said, we acknowledge our limited statistical power to detect a comparable phenomenon in wild sheep due to the lack of samples of pre-domestic Anatolian sheep. Thus, we cannot completely discard the possibility that the concentration of coalescences was, at least partially, due to pre-domestication events in mouflons. Further studies, especially those incorporating ancient mouflon specimens and whole genome sequencing data, could confirm or reject that such a pattern resulted from initial capture and human management of sheep, subsequent demographic changes, or demographic changes caused by non-anthropogenic factors, such as the climate conditions of the Younger Dryas cold spell.

### Impact of sheep management on microevolutionary processes at Aşıklı Höyük

One exciting possibility that our dataset allowed us to test is the hypothesis that management practices at Aşıklı Höyük caused the bottleneck suggested by the coalescence patterns in domestic sheep. However, nucleotide diversity, Tajima’s *D*, and Fu’s *Fs* statistics did not show significant changes across layers and levels (Fig. 3) and neither did the temporal tests on haplogroup frequencies (*P* = 0.28 to 0.97; fig. S2 and table S6). More powerful simulation–based analyses better supported a two-phased model with an increase rather than a decrease in population size (table S9). Although statistical support for this was moderate, a population increase is in line with zooarcheological evidence (*8*, *23*, *24*). Overall, our findings indicate that 1000 years of local management did not alter sheep mitogenomic diversity. Although our findings only refer to female ancestry, they nonetheless contradict the commonly held narrative that a genetic bottleneck accompanied early centuries of human management of livestock.

The constancy of diversity and neutrality indexes and haplogroups’ composition across archeological levels, combined with the presence in the phylogenetic reconstruction of sequences that coalesce during the site’s occupation (see Fig. 2 and figs. S3 to S7), suggests breeding continuity in the sheep of Aşıklı Höyük. The development of breeding practices could help explain the decline in the relative importance of game observed in the human diet during site occupation (*18*, *23*, *25*). However, the deep phylogenetic branching of some individuals at Aşıklı Höyük and the fact that some of these cluster with modern mouflons in haplogroups *E* may be a consequence of human exploitation of non–locally bred individuals. Those individuals can be compared to two Körtik Tepe individuals (Fig. 2, B and C, labeled K1 and K3) that coalesce, one at the base of haplogroup *B* and one to a deep branch that is paraphyletic to all mouflon and sheep individuals. The Körtik Tepe individuals, dated to 11.7 ka cal BP, are considered wild based on their archeological context, morphology, and dating (*53*). The possibility that the entire sheep population of Aşıklı Höyük was hunted or captured and raised [see, e.g., (*8*)] could explain the high diversity—and lack of bottleneck—at the site but is at odds with the mitogenomic relatedness that we observe and with the archeological evidence pointing toward dense stabling. Thus, we hypothesize that a minority of individuals at Aşıklı Höyük are likely wild sheep, which is in agreement with archeological evidence of hunted individuals that are distinguishable from the local stock, both in Aşıklı Höyük and in related sites (*8*, *23*).

The simultaneous presence of presumably wild and managed individuals in Aşıklı Höyük brings up an obvious question: Why? Although speculative, the presence of wild individuals may be explained by re-stocking that was used to compensate for losses caused by abortions and lamb mortality (*54*). It is also possible that some individuals at Aşıklı Höyük were exchanged with other communities of sheep keepers or that the founder herd of managed individuals was relatively large and diverse in geographic origin, possibly coming from somewhere else. A geographical translocation was the case for a “new” glume wheat that was coevally cultivated at Cafer Höyük, Aşıklı Höyük, and Boncuklu. This variety was not endemic to Central Anatolia but transferred long-distance from the Upper Euphrates valley (*7*, *55*, *56*). Since in this part of Anatolia, early cereal cultivation went along with sheep and goat management (*2*, *19*, *20*), a transfer of small stock on the hoof must be taken into consideration.

### Beyond Aşıklı Höyük

We found, in the phylogenetic reconstruction, multiple mitogenomes from modern and ancient Anatolian and European sheep that coalesced with mitogenomes from Aşıklı Höyük within 2000 years of the site’s occupation. This suggests that the genetic makeup of modern sheep populations retains a legacy from early Central Anatolia. As Central Anatolia participated in extensive trade networks since the Epipaleolithic (*57*), we assume that this legacy stemmed from an ancestral sheep metapopulation that included Aşıklı Höyük.

In contrast to Aşıklı Höyük, we found haplogroup *B* dominating later western Anatolian sites, Chalcolithic Güvercinkayası, and reported Anatolian sites dated between the Epipaleolithic and the Late Neolithic (*41*–*43*). Outside Anatolia, the same haplogroup appeared quasi-fixed in ancient and modern European and African sheep but became less dominant in the Levant and the Caucasus regions and turned out to be less prevalent than haplogroup *A* in modern sheep from central and eastern Asia. The spatial and temporal pattern of diversity and neutrality statistics, complemented and refined by the results of the temporal tests, suggests that the near fixation of haplogroup *B* in western Eurasia and *A* in central and eastern Asia resulted from demographic processes that postdate the occupation of Aşıklı Höyük yet predate the introduction of sheep to Europe (~8.5 ka cal BP) and central (~7.0 ka cal BP) and eastern Asia (~5.0 ka cal BP). More specifically, a bottleneck.

We tracked the origin of this putative bottleneck with several rounds of simulations-based analyses. These analyses revealed an approximately tenfold reduction in *N_e_* in the ancestral sheep population from which the populations in Neolithic southwestern Anatolia and Chalcolithic Güvercinkayası putatively descended (Fig. 5). This conspicuous bottleneck most likely occurred after Aşıklı Höyük had already been abandoned and coincided with the expansion of the Neolithic way of life beyond its formative zone. From the archeological record, the Neolithic expansion beyond Central Anatolia into western Anatolia and further (north-)west occurred by an inland route, via the Lake District, as well as through maritime expansion following the southern Anatolian coast, as has also been suggested recently (*58*–*60*). The migration of humans and their flocks was neither a single event nor a linear phenomenon, but a complex process involving various groups, which has recently been confirmed by the palaeogenomics of Neolithic Anatolian farmers (*61*). We therefore hypothesize that the demographic constraint observed in sheep could be the result of serial founder events that took place as sheep husbandry dispersed into (north-)western Anatolia.

Our analyses provide a definitive answer to previous reports that hinted at a possible gradient of increasing diversity and decreasing frequency of haplogroup *B* toward central and Eastern Anatolia and toward the Neolithic [e.g. (*41*, *42*)] but failed to track its origin. Our results indicate that the rise of haplogroup *B* was not the result of founder events that co-occurred with the introduction of sheep into Europe but rather related to a prior demographic process when sheep husbandry gained a foothold beyond Central Anatolia.

Our ABC analyses, MCMC-estimated historical demography, and Skyline Plots also support a substantial population expansion (~10×) in European sheep over the past ~5000 years (Figs. 3 and 4 and fig. S8). This demographic process can conceivably be the result of post-Neolithic human expansion, and an increase in genetic diversity related to the dispersal of wool sheep lineages originating in southwest Asia and beyond (*62*, *63*).

## MATERIALS AND METHODS

### Experimental design

To evaluate the mitogenomic diversity and the possible presence of a domestication bottleneck during the early stages of sheep management, we used a worldwide sample of mitogenomic sequences of sheep that was exceptionally dense in Neolithic southwest Asia and Europe. We computed population genetics statistics and applied multiple statistical analyses, including a phylogenetic reconstruction and simulation-based analyses by ABC. To better understand the numerous analyses of our study, consider that they pertain to three spatiotemporal scales. The first one focuses on Aşıklı Höyük, an aceramic Neolithic site in Central Anatolia with a long occupation span; at the next scale, analyses focused on Neolithic-Chalcolithic sites in Anatolia, and at the largest scale, our analyses involved all ancient and modern sites in Anatolia and Europe.

With respect to Aşıklı Höyük, we compared four subsequent archeological occupation layers that experienced substantial changes in sheep management. We first estimated and compared gene and nucleotide diversities, and the neutrality statistics *Fs* of Fu and *D* of Tajima (along with the associated statistical tests). Next, we tested the temporal changes in the haplogroup frequencies across layers, and afterward, we carried out simulation-based analyses by ABC for testing a set of possible scenarios of demographic change (or lack of it) across one millennium of site occupation while estimating their *N_e_*.

In a subsequent phase of analyses, we inferred the relationships between Aşıklı Höyük and the other ancient Anatolian samples in our sheep dataset, namely, Neolithic southwestern Anatolia and Chalcolithic Güvercinkayası. For this stage, we also compared distinct scenarios and inferred *N_e_* by means of simulations and ABC.

With the results of the previous analyses, we moved forward to study the relationship between European domestic sheep and their Anatolian ancestors. As in previous phases of analysis, we used simulation-based analyses by ABC to select the best statistically supported scenario among a suite of alternatives. With the selected scenario we carried out a second simulation-based inference (by ABC) to estimate *N_e_* for all populations of the selected scenario, including Aşıklı Höyük, Neolithic southwestern Anatolia, Chalcolithic Güvercinkayası, and ancient and modern Europe.

### Sampling

Samples were obtained from three sources (see tables S4 and S5 for details and table S14 for contact information).

1) De novo sequences obtained from direct sampling of both live specimens and ancient remains. The set of ancient samples resulted from the study of >500 bone and tooth specimens from archeological sites and museum collections in southwest Asia and Europe, and subsequent processing through targeted capture and next-generation sequencing.

2) Sequences retrieved from public repositories of high throughput sequencing data by means of data mining techniques (see text S7).

3) Published mitogenomes. We gathered mitogenomes associated with published studies or directly from GenBank. Published data from archeological sheep originated from sites in Bulgaria, Georgia, Germany, Ireland, Israel, Malta, Serbia, Türkiye, and the United Kingdom. Details of the samples including age, geographical origin, and haplogroup are shown in table S5.

### DNA extraction and sequencing

We processed modern and ancient samples for DNA extraction, polymerase chain reaction, mitochondrial DNA (mtDNA) bait capture of sequencing libraries, and sequencing, in separate facilities, following established protocols (see texts S1 to S6). We processed ancient DNA samples in clean room facilities designed to deal with ancient and highly degraded materials both at the University of Munich (LMU) and Trinity College Dublin. Sequencing took place at the Gene Center, LMU, and the TrinSeq facility, Trinity College Dublin. We aligned sequences and generated consensus FASTA files as described in texts S4 to S6. The final database of 629 mitogenomes was initially subject to five packages of statistical analyses.

### Haplogroup identification and population genetics indexes

After alignment, we identified haplogroups *A* to *E* by using the published sequences in our dataset as a reference. These haplogroups displayed split times older than 30 ka cal BP. We called *Z* a new haplogroup constituting a deep monophyletic clade that was paraphyletic to haplogroups *A* and *B*. Another unreported mouflon-exclusive haplogroup was named *G*. This haplogroup is polyphyletic, though this was not problematic since it was not used for statistical inference. We noticed that haplogroup *A* contains two individuals that diverged so deep that it would even challenge their haplogroup calling. However, we kept their classification as *A*, as their impact on the statistical analysis would be negligible or null.

After assigning haplogroups to all samples, we grouped all samples into 12 groups based on temporal and geographic closeness and computed haplogroup frequencies for them. In addition, we subdivided the sample from Aşıklı Höyük into four groups corresponding to different archeological occupation layers. Last, we used these 12 + 4 sample groups to estimate the average number of pairwise differences, nucleotide diversity, and gene diversity as well as to compute the neutrality tests, and the corresponding indexes, of Tajima (*D*), Fu (*Fs*), Ewens-Watterson, and Chakraborty. We computed diversity and neutrality statistics and tests with the software Arlequin v3.5.2.2 (*64*) and MEGA X v10.0.5 (*65*).

### Tests of temporal change in haplogroup frequencies

To discard gene drift and sampling error as the causes behind the observed differences in haplogroups, we applied temporal tests of allele frequencies, here applied to haplogroup frequencies. We applied these tests among all sample pairs that had different ages. The rationale is that a significant result with these tests would suggest the presence of evolutionary forces, other than gene drift under a constant population size. Those forces could be selection or gene flow but also intense demographic changes. We applied simultaneously four tests for every comparison: a conventional contingency-table chi-squared test, the test of Waples (*66*), and a Bayesian test that includes the estimation of a temporal version of the statistic *F*_ST_ (*67*). The Waples and the Bayesian tests are statistically more suited and accurate for testing temporal changes in genetic diversity but require knowledge of *N_e_* values. To bypass this problem, we tested five values of *N_e_* for each comparison (10^2^, 10^3^, 10^4^, and 10^5^). For these analyses, it was necessary to group temporally some samples that displayed different ages but were geographically and chronologically close (see Table 1 and table S6). Because of their large number, we presented the comparisons in two groups: only ancient Anatolian samples, and comparisons among Anatolian samples and other samples (see fig. S1).

### Phylogenetic analyses

We reconstructed a temporally calibrated phylogenetic tree, taking advantage of our high coverage time-stamped mitogenomes, and inference of historical demography by means of an extended Bayesian skyline plot associated with the phylogenetic inference (*68*). We used all modern and ancient mitochondrial sequences reported here, including a urial specimen (*O. vignei*) as an outgroup. We partitioned the sequences in three codon positions (all protein-coding genes), tRNAs, rRNAs, and noncoding regions, and used the HKY85 + G as a substitution model, following the result of the model selection tool implemented in MEGA X v10.0.5 (*65*). For the phylogenetic inference, we used the Bayesian method with trees of the posterior set sampled by MCMC, as implemented in the software BEAST v2.6.6 (*68*). We used the software BEAUti (*69*) to create the input files. We carried out a pre-run of 100 million generations to obtain operator diagnostics for improving convergence, and two final parallel runs with 25% burn-in and an overall of one billion generations each. The full run allowed most parameters to reach the minimum effective sample size of 200. Mixing was assessed using the software Tracer (*70*).

To infer the extended Bayesian skyline plot (*71*, *72*), we ran three separate runs: the first one using samples from modern and ancient Europe and Neolithic sites Aşıklı Höyük and Körtiktepe, the second one with the previously described set plus samples from Neolithic southwestern Anatolia (Suberde, Çukuriçi, Menteşe, and Marmara regions), and the third one using only samples of modern mouflons.

### Demographic inference on coalescent times

In our phylogenetic reconstruction, we found a very high density of nodes between 12.0 ka and 7.6 ka cal BP, inside the clades corresponding to haplogroups *A* to *E* (excluding mouflons). To test whether this high density of coalescences is the result of a founder event or an artifact of having numerous ancient samples with broadly contemporaneous ages, we applied a Monte Carlo statistical procedure to the set of coalescent times and samples ages: the first ones obtained from the BEAST-estimated nodes’ ages and second ones from the radiocarbon- and stratigraphy-estimated samples ages. This procedure, developed in-house, takes advantage of the known mathematical relationship between demographic history and inter-coalescent times to sample the demographic history of Anatolian and European sheep by means of MCMC. The inference should produce an approximately constant demographic history if the increased density of coalescence events was solely due to sampling bias (see details in text S8).

### Simulation-based inference by approximate Bayesian computation

The latest and most extensive package of analyses consisted in a series of simulation-based analyses performed by ABC (*73*). ABC analyses were performed in four phases, each with a specific inferential goal. They were the following:

1) To test and estimate the changes in *N_e_* between the subsequent excavation layers of Aşıklı Höyük. These analyses included an analysis that used pseudo-observed datasets to estimate the statistical power that our methodology had to detect a bottleneck around Aşıklı Höyük.

2) To select the best model of ancestry relationships among the three sampled ancient Anatolian populations, namely, Neolithic Aşıklı Höyük, Neolithic southwestern Anatolia, and Chalcolithic Güvercinkayası.

3) To select the best ancestry model for the European population out of the ancient Anatolian populations.

4) To estimate with improved precision the population sizes in all involved populations, from Aşıklı Höyük to modern Europe and thus confirm or discard the presence of a domestication bottleneck.

All analyses included optimization and inferential steps, and the fit of the summary statistics was controlled by visual inspection on the predictive distribution of the summary statistics (figs. S15 and S16). All analyses were also replicated with different sets of summary statistics, as well as with a procedure based on random forests, a type of ABC inference assisted by machine learning (see text S9 and tables S7, S8, S10, and S12 for details on the simulation-based inference by ABC).

### Ethics statement

All work involving living animals was conducted according to the national and international guidelines for animal welfare. Blood samples were collected with owner consent during routine examinations under good veterinary practice. Sampling was approved by the regional government of Upper Bavaria (55.2-1-54-2532.0-47-2016).

## Supplementary Materials

### This PDF file includes:

Texts S1 to S10 Figs. S1 to S16 Tables S1 to S14 References
