# Supplementary material for: Ancient mitogenomes from Pre-Pottery Neolithic Central Anatolia and the effects of a Late Neolithic bottleneck in sheep (Ovis aries)
Source: Sci Adv. 2024 Apr 12;10(15):eadj0954. doi: 10.1126/sciadv.adj0954 (PMC11014441; doi:10.1126/sciadv.adj0954)
Supplement: Supplementary file 1 — Texts S1 to S10 Figs. S1 to S16 Tables S1 to S14 References [file sciadv.adj0954_sm.pdf]

Science Advances
AAAS

Supplementary Materials for

Ancient mitogenomes from Pre-Pottery Neolithic Central Anatolia and the effects of a Late Neolithic bottleneck in sheep (*Ovis aries*)

Edson Sandoval-Castellanos *et al.*

Corresponding author: Edson Sandoval-Castellanos, edson.sandoval@gen.vetmed.uni-muenchen.de;
Ivica Medugorac, ivica.medjugorac@gen.vetmed.uni-muenchen.de; Daniel G. Bradley, dbradley@tcd.ie;
Joris Peters, joris.peters@palaeo.vetmed.uni-muenchen.de

Sci. Adv. **10**, eadj0954 (2024)
DOI: 10.1126/sciadv.adj0954

This PDF file includes:

Texts S1 to S10
Figs. S1 to S16
Tables S1 to S14
References

Supplementary Text S1. Ancient and modern sample preparation

Each bone sample was irradiated with UV light from a short distance for 30 minutes (UV hand lamp 115C with lamp stand, 254 nm, Peqlab). Then, in a DNA workstation I (Kisker Biotech), approximately 1 mm of the bone surface was removed with a mill/drill unit (Micromot 50/E, Proxxon) at the position intended for sampling. The milling head was then flamed with a staff lighter and the drill cleaned with DNA away™ (Carl Roth). Subsequently, about 250 mg of sample powder were obtained with the mill/drill tool. Before processing a new sample, the instruments used and the workstation were cleaned with DNA away™, the milling head of the drill was flamed, and laboratory gloves changed.

For modern samples, DNA from previous projects of collaborating groups or EDTA blood was used as starting material for further molecular genetic analyses.

Supplementary Text S2. DNA extraction of ancient and modern DNA samples

DNA extraction from ancient material combined the extraction protocols of Rohland et al. (74) and Rohland and Hofreiter (75). Rohland et al. (74) tested the ratio of binding buffer to extraction buffer, with the best results obtained when binding and extraction buffers were in a 1:2 ratio. Extraction and binding buffers were prepared according to the protocol of (74). The described wash buffer, elution buffer, and the preparation of the silica suspension are identical in both protocols. The following extraction steps were performed according to the protocol of (74): To about 250 mg of sample powder, 5 mL of extraction buffer were added, and the samples were then incubated at 37° C in a warming cabinet under constant agitation. The incubation period was extended to approximately 40 h. Subsequently, the samples were centrifuged for at least 2 min at 5,000 × g and the supernatant transferred to a new 15 mL tube. To the supernatant, 2.5 mL of binding buffer and 100 µL of silica suspension were added and the samples were incubated for 3 h under constant agitation in the dark. The samples were then centrifuged for 2 min at 5,000 × g and the supernatant discarded. The subsequent extraction steps were carried out according to the protocol of (75): 1 mL of binding buffer was added to the silica pellet and the silica re-suspended and transferred to a 2 mL tube. This was followed by the described purification steps. The silica pellet was finally dried at room temperature with open lids for about 15 min. To the dried silica pellet 50 µL elution buffer was added, the silica was re-suspended and incubated for about 10 min with closed lids. After centrifugation at 16,000 × g for 2 min, the supernatant was finally transferred to a new 1.5 mL tube. All DNA extractions were accompanied by extraction controls.

DNA extraction of tooth and petrous bone samples was performed with minor changes according to the method of Dabney et al. (76). 80 mg to 180 mg of sample powder were used per sample to which 2 mL extraction buffer were added. The incubation time was extended to approximately 40 h. As a binding apparatus the High Pure Viral Nucleic Acid Large Volume Kit (Roche) described by Korlevic et al. (77) was used. After two wash steps with PE buffer (Qiagen), two elution steps with 25 µL elution buffer EB (Qiagen) were performed, resulting in 50 µL DNA extract for each sample.

For modern samples, the commercially available reagent kit DNeasy Blood & Tissue Kit (Qiagen, Germany) was used to extract DNA from ovine blood samples according to the manufacturer's instructions.

Supplementary Text S3. PCR of ancient and modern DNA samples

In the initial phase of the study, the following PCR primer sets were used to test the quality and the haplogroup of the ancient DNA: Ovis 110 bp (*cytochrome b*, *CYTB*), nt positions 14578 – 14687 (accession nr. AF010406, haplotype *B*) with a sequence framed by the primer pair of 67 bp (nt positions 14601 – 14667) using the primer pair 110-F (5’-CTGAGGAGCAACAGTTATTACCA-3’, nt positions 14578 – 14600) and 110-R (5’-GGGTGAGGGAGCTTTGTCT-3’, nt positions 14687 – 14668), Ovis HVRI, 137 bp, nt positions 16051 – 16187 (accession nr. AF010406, haplotype B) with a sequence framed by the primer pair of 97 bp (nt positions 16071 – 16167) using the primer pair 137-F (5’-GATCACGAGCTTGTTCACCA-3’, nt positions 16051 – 16070) and 137-R (5’-GCCCTGAAGAAAGAACCAGA-3’, nt positions 16187 – 16168) as well as the primer set Ovis HVRI, 118 bp, nt positions 16175 –16292 (accession nr. AF010406, haplotype *B*) with a sequence framed by the primer pair of 78 bp (nt positions 16195 – 16272) using the primer pair 118-F (5’-

CTTTCTTCAGGGCCATCTCA -3', nt positions 16175 – 16194) and 118-R (5'-ACCAAATGCATGACACCACA-3', nt positions 16292 – 16273).

Conventional PCR: PCRs were performed in 20 µL reaction mixtures consisting of 50 mM KCl, 15 mM Tris-HCl (pH 8.0), 4 mM MgCl2, 0.250 mM dATP (Peqlab), 0.250 mM dCTP (Peqlab), 0.250 mM dGTP (Peqlab), 0.500 mM dUTP (Peqlab), 0.8 mg/mL UV irradiated bovine serum albumin (BSA; Thermo Scientific), 0.20 µM each primer, 2 U AmpliTaq Gold DNA polymerase (Life Technologies), 0.5 U uracil-DNA glycosylase (UDG), heat-labile (Affymetrix), and 2 µL ancient DNA extract. Initially, an incubation step with uracil-DNA glycosylase was performed at 37°C for 15 min. Amplification cycles were started by an initial denaturation step at 94°C for 10 min, followed by 45 cycles of: denaturation at 95°C for 45 sec, annealing at specific temperature for 40 sec, extension at 72°C for 40 sec. PCR was finished by final extension at 72°C for 10 min. The amplification reactions were carried out in a TPersonal thermal cycler (Biometra).

Real-Time PCR: qPCRs were performed in 20 µL reaction mixtures consisting of 0.8 mg/mL UV irradiated bovine serum albumin (BSA; Thermo Scientific), 0.20 µM each primer, 10.0 µL Fast SYBRTM Green Master Mix (2x mix; Applied Biosystems) and 2 µl ancient DNA extract. The Fast SYBRTM Green Master Mix contains uracil-DNA glycosylase (UDG).

Initially, an incubation step at 37°C for 10 min was performed. Amplification cycles were started by an initial denaturation step at 95°C for 20 sec, followed by 50 cycles of: denaturation at 95°C for 5 sec, annealing at specific temperature for 20 sec, and extension at 60°C for 10 sec. Amplifications were followed by a dissociation gradient from 65°C to 95°C with 0.5°C / 5 sec. The qPCRs were carried out in a CFX96 Touch™ Real-Time PCR Detection System (Bio-Rad).

For the three primer sets the following annealing temperatures were used: 110-F/110-R (58°C), 137-F/137-R (56°C), 118-F/118-R (56°C).

For modern samples, mitochondrial DNA was amplified with three overlapping large PCR products spanning the whole circular genome. The following PCR primer pair were used: (i) MitoOAR1F (5'-AGCAATTCCCATAGCCTCCT-3', nt positions 13654-13673) and MitoOAR1R (5'-GAAACAAACCGAGCACCATT-3', nt positions 3318-3337) with PCR product of 6320 bp, (ii) MitoOAR2F (5'-GAAAAGGCCCAAACGTTGTA-3', nt positions 2845-2864) and MitoOAR2R (5'-TTCACTCGCCCATTTCCTAC-3', nt positions 8304-8323) with PCR product of 5460 bp, and (iii) MitoOAR3F (5'-AGTCAACAACCGCCTCATCT-3', nt positions 8046-8065) and MitoOAR3R (5'-CCAACCCCACCACTTACAAT-3', nt positions 13967-13986) with PCR product of 5920 bp.

PCRs were performed in 12.5 µL reaction mixtures consisting of 2.25 µl GoTaqLong PCR Master Mix, 0.9 µl upstream primer, 0.9 µl downstream primer, 1.0 µl template DNA 15ng/µl and 3.45 µl Nuclease-Free Water. Amplification cycles were started by an initial denaturation step at 94°C for 2 min, followed by 10 cycles of: denaturation at 94°C for 30 sec, annealing at 61°C for 30 sec, extension at 70°C for 36 min and 30 sec followed by 25 additional cycles of: denaturation at 94°C for 30 sec, annealing at 60°C for 30 sec, extension at 70°C for 6 min and 30 sec plus 10 sec per cycle. PCR was finished by final extension at 72°C for 10 min. The amplification reactions were carried out in a TPC-100 thermal cycler (MJ Research Inc.).

Supplementary Text S4. DNA sequencing of short ancient DNA amplicons

Purified amplification products were directly sequenced with the BigDye Terminator v1.1 Cycle Sequencing Kit and a 3730 DNA Analyzer (Applied Biosystems). The 10 µL cycle sequencing reaction mix contained 4.62 µL PCR grade water (Thermo Fisher Scientific), 1.63 µL 5× Sequencing buffer, 0.75 µL BigDye Terminator Sequencing Mix v1.1 (2.5×), 1.0 µl of 5.0 pmol/µl sequencing primer, and 2.0 µL DNA template. Cycling conditions were as follows: an initial denaturation step at 96°C for 1 min, followed by 35 cycles of: 96°C for 10 sec, 55°C for 15 sec, and 60°C for 4 min; and 4°C until storage. Instrument: TPersonal thermal cycler (Biometra). Clean-up of the reaction and the sequencing runs were performed by the Sequencing Service of the Genomics Service Unit, Faculty of Biology, Biocenter, Ludwig Maximilian University of Munich (LMU).

Supplementary Text S5. TCD ancient samples preparation and sequencing

Ancient material processed at Trinity College Dublin was done so in dedicated ancient DNA facilities as previously described in (78). Briefly, 125 mg of bone powder was obtained from cleaned, surface UV-irradiated bone via pulverisation. DNA was extracted from bone powder using a 48-hour EDTA-proteinase K digest at 37°C following an initial 30 min EDTA pre-digestion. Supernatant was separated from the remaining bone powder via spin-down and subjected to cleanup using MinElute columns, with DNA eluted in 40ul. To reduce DNA damage, purified DNA was USER™ treated: 16.25ul of DNA with 5 µl (1U/1uL) USER™ were incubated for 3 hours at 37°C. USER treated DNA was then used in dsDNA library construction (79), modified by Gamba and colleagues (80), followed by Accuprime Pfx supermix amplification for 10-16 cycles to reach ~5ng/ul. DNA was quantified using an Agilent Tapestation 2200.
Libraries were sequenced using an Illumina HiSeq 2500 (Macrogen, Seoul). After bioinformatic processing (see below), samples with low (< 5%) endogenous DNA content were subjected to mitochondrial read enrichment using RNA baits (Daicel Arbor Bioscience) following manufacturer's protocol for degraded DNA as described in (78). Captured libraries were not subjected to the uracil-DNA glycosylase treatment. Enriched, amplified libraries were sequenced using an Illumina MiSeq (TrinSeq, Dublin).

Supplementary Text S6. LMU ancient samples preparation and sequencing

Library preparation

Library preparation was performed, with slight modifications, according to the protocol of Gansauge and Meyer (81). For the library preparation, 10 µL of ancient DNA extract were used per sample. In addition, water controls, extraction controls and samples not treated with the enzyme Archaeoglobus fulgidus (Afu) uracil-DNA glycosylase (New England Biolabs) were carried along.
First, it was tested whether the amount of CircLigase II (Epicentre) added per sample could be reduced while prolonging the incubation time, as this enzyme is very cost-intensive. The amount of CircLigase II per sample was finally reduced by half.
Thus, after treatment of the samples with the enzymes Afu uracil-DNA glycosylase, endonuclease VIII (New England Biolabs), and FastAP alkaline phosphatase (Thermo Fisher Scientific), 2 µL instead of 4 µL CircLigase II were added per sample. In this context, the reaction volume was set to 60 µL instead of 80 µL and the incubation time for the ligation of the first adapter was extended from 1.0 to 3.0 h. The following steps, i.e. immobilization of ligation products on beads, primer annealing and extension, blunt-end repair and ligation of the second adapter, were performed exactly as described by Gansauge and Meyer (81).
Subsequently, the beads were resuspended in 30 µL TET buffer, the bead suspension was incubated at 95°C in a ThermoMixer MKR13 (HLC/DITABIS) for 2 min and then immediately transferred to a magnetic rack (DynaMagTM-2 magnet, Life Technologies). Finally, the supernatant was pipetted into a 0.5 mL tube and frozen to -20 C.
The library amplification and indexing was performed in a reaction volume of 70 µl.
Index Primers from Index Primers Set 1 and Index Primers Set 2 (NEBNext® Multiplex Oligos for Illumina®, New England Biolabs) were used as "P7 indexing primers".
The reaction conditions corresponded to the protocol of Gansauge and Meyer (81), whereby 15 cycles were carried out in a Peqstar 2× gradient thermocycler (Peqlab).
The subsequent purification was performed with the MinElute PCR purification kit (Qiagen). Since the AccuPrime Pfx Reaction mix (Life Technologies) is alkaline (~ pH 9.0), 2 µL of a 3 M Sodium acetate buffer (pH 5.2, Sigma Aldrich) were added to each sample during purification. Finally, the libraries were eluted in a 26 µL elution buffer and frozen to -20°C.
Regarding library amplification and indexing, the reaction mixture was prepared in a laboratory of the ArchaeoBioCenter, but amplification and indexing reactions were performed in the Biocenter.

Sequencing and assembly of mitogenomes

For ancient samples barcoded libraries were pooled and enriched by hybridization to an Agilent SureSelect capture array with 244k oligo spots. The oligos were designed to include all mitogenome variants observed by sequencing a panel of European breeds included in this study. After a 65-hour hybridization in the presence of cot-I sheep DNA and adapter blocking oligos at 65°C, the capture array was washed, and the captured library molecules were eluted at 95°C for 10 min in a volume of 500 µL of DNA-grade water. The enriched libraries were then amplified by PCR, analyzed on the Bioanalyser and sequenced in 2*50bp paired end mode on a HiSeq1500 sequencer from Illumina. The custom sequencing Primer CL-72 (5'-ACACTCTTTCCCTACACGACGCTCTTCG-3') was used for read-1.

For modern samples, 2µl of three overlapping long PCR products (6320 bp, of 5460 bp and 5920 bp) were visualized in 1% agarose gel electrophoresis separately. Three PCR products from a single animal that were clearly visible and of the expected length were pooled in a 1.5ml tube and were purified with Wizard SV Gel and PCR Clean-Up System. 1.0 ng of pooled and clean PCR products were converted to sequencing libraries by tagmentation (NexteraXT, Illumina, San Diego USA) following the manufacturers instruction. Dual-barcoded libraries were pooled in equimolar amounts and sequenced in 2*100 bp mode on a HiSeq1500 sequencer from Illumina.

The reads obtained by sequencing of short-read mtDNA libraries were clipped to remove the sequencing adapter, length filtered to >20bp and mapped to mitogenome reference (GenBank NC_001941.1, concatenated with human mitogenome MF479165.1 to exclude spurious contamination by human DNA) using a Burrows–Wheeler transform (82). After removal of duplicate reads, variants were called with VARSCAN (83) and replaced in the reference fasta file. This adapted reference was used for iterative mapping and variant calling until no further variants could be identified. The resulting fasta file of the assembled mitochondrial genome, masked for bases with no coverage, was used for further analyses.

Supplementary Text S7. Online sourced mitogenomes

Our dataset included 309 samples sourced from published studies and public databases (details in Tables S4-S5). From them, 172 were sourced from whole genome-sequencing data and from them 166 mitogenomes were extracted from the NCBI Sequence Read Archive. For this, we downloaded the data converted to fastq files that were later mapped to a reference mitogenome and then filtered to keep only reads that mapped to the mitochondrial genomes. All the bioinformatics procedures involved in this step, as well as the validation, alignment and annotation of the obtained sequences are identical to those described in (50).

Supplementary Text S8. Demographic inference on coalescent times

The phylogenetic tree, in the clade that corresponds to the domestic sheep, contains an apparent excess of nodes around 12.0-7.6 ka cal BP that can be either the signature of some events associated with domestication or a byproduct of having a proportionally large number of ancient samples in the mentioned interval. To choose between these alternatives, we designed an analysis based on the coalescent theory to link coalescent times (nodes) and a demographic history. It consisted in an MCMC procedure that sampled the posterior distribution of demographic histories conditional to the empirical data. The "empirical" data was constituted by the set of coalescent times (nodes) estimated with the BEAST phylogeny plus the entire set of samples' ages. The historical demography consisted in a time-series of effective population size values (N_e). The posterior probabilities of the MCMC were obtained from the probability of a coalescent happening at time t, conditional to the effective population size N_e:

$$P(T=t) = e^{\frac{kt}{2N_e}} * (2N_e)^{-1} \quad \text{, Equation 1 (84)}$$

where t is the time duration of the period, and k is the number of combinations of 2 in X (X is the number of lineages available for coalescence). If the number of lineages changes due to a coalescent event (reducing the number of available lineages) or the appearance of ancient samples or migrants (increasing the available

lineages) then the "lack of memory" property of the coalescent can be invoked, recalling that the coalescent is a continuous time Markov chain. Because of that, the entire probability can be calculated piecewise using Equation 1 upon time bins where both N_e and the number of lineages are constant, and the whole probability being the product of them all. This still can approach any conceivable demographic history with an adjustable degree of resolution. It is also mathematically very convenient because the use of non-stepwise functions would require the solution of analytically unsolvable integral equations (except for functions of exponential growth).

To calculate the probability of the "empirical" data (coalescent times and samples' ages) given a defined demographic history the vector of inter-arrival times is made not only considering the coalescent times and samples ages but also the boundaries of the time-bins of the demographic history (see Fig. S1).

With this simple procedure we carried out an MCMC procedure that sampled the parametric space of demographic histories. We inferred demographic histories with 16-, 32-, and 64-time bins. The time boundaries of the 16-bins demographic histories were: 0.0, 1.0, 2.0, 3.0, 4.0, 5.0, 6.0, 7.0, 8.0, 9.0, 10.0, 11.0, 12.0, 13.0, 14.0, 15.0, and 25.0 ka cal BP. The 32-bins and 64-bins histories just had the same periods split by half or split in four equal-sized bins.

Convergence was assessed by running two chains in parallel and comparing the demographic histories of them. The differences among averaged histories, measured as the sum of differences of time bins were under 1% after 100-200 thousand generations. We ran 10 million generations sampled, stored every 10,000 generations, and used a 25% burnin for analysis. We used a Metropolis-Hastings algorithm with a normal distribution used as the jump distribution with standard deviation set to 2.5% the parameters range. The prior distributions of the demographic histories consisted in a set of log-uniform distributions for each N_e value with boundaries in 10^2-10^7. The software was coded, debugged, and compiled in Fortran 90 with the assistance of the IDE implemented in Microsoft Visual Studio Professional 2015 version 14.0.25431.01 update 3, with Fortran compilers from the package Intel® Parallel Studio XE 2018 update 1. The software is available at: https://doi.org/10.5281/zenodo.10392203.
This analysis allowed us to assess the potential effect of the ancient samples in the accumulation of coalescent events around 12.0-7.6 ka cal BP. If such an accumulation was a sampling artefact, the estimated demographic history would be non-informative, flat, or noisy, around the focal period.

Supplementary Text S9. Simulation-based inference by approximate Bayesian computation

To better understand the relationships among the sampled ancient and modern populations, and to infer their demographic parameters, we carried out several rounds of statistical analysis by means of approximate Bayesian computation, or ABC (73, 85). ABC performs statistical inference by running large amounts of simulations under a specific model (in our case the coalescent model of molecular evolution). ABC methods use simulations that link the known data with unknown variables (parameters) that are sampled from prior probability distributions and create simulated datasets that are theoretically equivalent to the empirical data. Parameters are inferred from posterior probability distributions approached by a rejection algorithm that ranks simulations according to the distance between the empirical and the simulated datasets and rejects simulations out of a threshold or by selecting the k-nearest neighbours of the empirical data. For calculating the distances ABC employs summary statistics that ideally should capture all the relevant information of the datasets. To perform hypotheses contrasts, called model choice analyses, ABC approaches the model likelihoods from the acceptance ratios of the different models in the non-rejected (accepted) set.
We used ABC for four inferential goals plus one validation analysis, each (except the first one) employing the results of previous ones:

1. The first phase was aimed to determine the demographic differences among layers of Asikli Höyük. For this, we carried out two analyses, a parameters inference of the effective population size (N_e) in each layer and a model choice analysis (hypotheses contrast) with three models: (i) a continuous model over the entire occupation timespan of Asikli Höyük; (ii) a model with two periods with boundary around 9.7 ka cal BP; and (iii) a model with four periods, one for each sampled layer (dated at 9.425, 9.625, 9.850 and 10.150 ka cal BP) (see Fig. S9). All models also included one pre- and one post-occupation period containing samples

from Körtik Tepe and Güvercinkayası, respectively. The time boundaries of all periods had some variability (set with priors) following time constrains of the samples.

As an extension of phase one, we carried out an analysis of PODs (Pseudo-Observed Dataset Analysis) to test the statistical power of the ABC methodology to detect demographic changes at Asikli Höyük. The rationale of this analysis is that simulated datasets can be used as empirical data to estimate the statistical power to detect the alternative scenarios in a model choice analysis (hypothesis contrast). For this, the datasets created in the simulations under every model are used as empirical data to perform model choice analyses by ABC many times recording the number of times when the analyses selected the right model. The PODS analysis used simulations under model A of phase one (Fig. S9) but with the population sizes being fixed according to the alternative models. We tested 21 models, each with a different size in the Aşıklı Höyük population: 10 models had larger sizes, 10 smaller, and 1 had the same size as the previous population. Each of the 21 models required a full PODs analysis with 1,000 PODs (i.e. 1,000 rounds of post-simulation analysis) with instrumental tables made of 125,000 simulations for each of the 21 models.

2. The second phase consisted in a model choice analysis for selecting the best scenario of evolutionary relationships among the sampled ancient Anatolian populations, namely, Neolithic Aşıklı Höyük, Calcolithic Güvercinkayası, and Neolithic southwestern Anatolia. The models included a scenario where all samples belonged to a single continuous population as well as a scenario where all three populations split from a common ancestor during the Epipaleolithic (see Fig. S10).
3. The third phase was also a model choice analysis aimed to select the best model describing the evolutionary relationship of the European population (including both ancient and modern) with respect to the ancient Anatolian populations. The five tested models were constituted by scenarios where the European sheep population descended from either Neolithic Aşıklı Höyük, Calcolithic Güvercinkayası, Neolithic southwestern Anatolia, or an unrelated (and unsampled) ancestral population (Fig. S11).
4. The fourth phase was an extension of phase three, where the select model (Europe descending from Neolithic southwestern Anatolia) was used to carry out a parameter estimation analysis focused on population sizes (N_e) of all the eight populations involved in the time-space structure of the used model (Fig. S11-C).

All analyses described above were performed in two steps each: the optimization step and the inferential step. The optimization step consisted in a pre-run of typically one million simulations (for each model) and the obtained posterior distributions were used to set the priors of the inferential runs. This was performed in two ways: if the posterior was a relatively narrow, symmetrical, and bell-shaped, then a normal distribution (usually a log-normal for population sizes and mutation rate) was adjusted by matching the 95% probability boundaries of the posterior and new distributions and verifying the closeness of their distribution means. If the posterior was not bell shaped, then the adjustment only consisted in re-defining the boundaries of a uniform or log-uniform prior.

For the inferential step, the model choice analysis only required $1.25\text{-}2.5\times10^6$ simulations (per model) while parameters estimation analyses were performed with 1.0×10^6 simulations. Phase 3, however, got a boost in the number of simulations to 2.4×10^7 for improving accuracy and reducing the approaching error.

We replicated all post-simulation analyses to address Bayes sufficiency. This is because a high number of summary statistics improves the Bayes sufficiency problem i.e. the need to capture the relevant information of the data (here, the DNA alignment) (86) whereas a low number of summary statistics improves the statistical power, reduces the approaching error, and ameliorates the accuracy loss due to high dimensionality, a phenomenon called “the curse of dimensionality” (87). We observed such a problem in finding different parameters being better estimated with different sets of summary statistics while the use of a composite set produced worse results (e.g. high variance posteriors). To fix that, we analyzed in parallel three to six sets of summary statistics and reported the posteriors of an intermediate set with the highest consistency (see Tables S7-S10).

We also replicated all model choice analysis with a machine learning-assisted method termed random forests-ABC (86). This method, which is within the spectrum of ABC methods, offers several advantages including a theoretical resilience to the “curse of dimensionality” that affects conventional ABC. All this to push the boundaries of the statistical capabilities of ABC to detect the magnitude and placement of the presumptive

demographic events associated with domestication. Following its creators' advice, we ran the random forests-ABC procedure with 100,000 simulations per model and used all summary statistics available, namely: HapTypes, PrivHaps, SegSites, PairDiff, NucDiver, GenDiver, TajimasD, and FusFs for each statistical group (population); and PairDifG, Fst, SharedHap, and ShareFreq for each pair of statistical groups (populations). See the definition of the summary statistics in Table S7.
The coalescent simulations and the post-simulation analysis (rejection, Eppanechnikov adjustment, regression procedures) were performed in an updated cluster-compatible version of the software BaySICS (88), available at https://doi.org/10.5281/zenodo.10210930. The updates to BaySICS were coded, debugged, and compiled in Fortran 90 with the assistance of the IDE implemented in Microsoft Visual Studio Professional 2015 version 14.0.25431.01 update 3, with Fortran compilers from the package Intel® Parallel Studio XE 2018 update 1.

Supplementary Text S10. Archaeological information

Bulgaria

The site of Dzhulyunitsa (or Džuljunica) is located north of the Balkan Mountains and south of the Danube basin, in north central Bulgaria. The four stratified layers of the settlement are radiocarbon dated to c. 8.15–7.45 ka cal BP, covering the entire sequence of the Early Neolithic in southeastern Europe. Dž–I is contemporary with the pre-Karanovo I and Karanovo I cultures of the Early Neolithic in Bulgaria. Dž–II is characterized by rather thick deposits partly overlapping with Dž–I. Dž–III is a levelling layer. Dž–IV is characterized by a drastic decrease in the settlement size, and new ceramics representing the early 6th millennium BCE (89). Cattle and caprines were important, while suids were avoided throughout the sequence (90). Samples from this site are in rows 457-458 of Table S5.

Georgia

Tamara's Fort, also called Dariali Fort (Coordinates: UTM 38N 469400, 4731800), is found in the border zone between Georgia and Russia in the Kazbegi region, sitting atop a high flat outcrop on the west bank of the Tergi river. The site was investigated by Eberhard Sauer (Edinburgh University) and colleagues in the ERC project "Persia and its Neighbors".
Excavations at Tamara Fort indicated several occupations, focused between the Sassanian era (3rd-7th centuries A.D.) and until the 9/10th centuries during which period its function was a military fort. Between the late 13th and early 15th centuries AD the site was re-occupied, but there is no evidence for occupation following this until the 20th century (91).
Approximately half a ton of animal bones was analyzed during four excavation seasons, supervised by Marjan Mashkour. Domestic herbivores (cattle, sheep, and goats) are dominant in the faunal remains, with a single domestic goat genome showing Iranian-like ancestry (78). Caucasian tur specimens were discovered among the remains (91), which showed East Caucasian tur (*Capra cylindricornis*) genetic affinity at the nuclear and mitochondrial genome levels (92).
14 samples are reported here, corresponding to rows 504-517 of Table S5.

Germany

The Alzey site is located in western Germany, in the state of Rhineland-Palatinate. The site is dated to Roman era, at approximately the second century A.D. The sample from here has been classified as Iron Age in all analyses, given the Roman era was considered part of the historic Iron Age (93). The sample from this site is in row 461 of Table S5.
A single sample included in this project was from the Roman site of Nida-Heddernheim in Frankfurt, from the state of Hesse in Germany. The sample was archaeologically dated to the Iron Age, in the second century, contemporary with Roman era samples also included in this project. Four samples were from Mainz in

Germany. These samples were dated to the second century A.D. and are contemporary to the sample from Frankfurt. The samples from these sites are in rows 459, 460, and 462-464 of Table S5.
The site Herxheim in southwest Germany is a Neolithic site, associated with the linear pottery culture (LBK). The site was occupied for three to four hundred years, from about 7,000 years ago. The site is famous for containing a mass grave. Three samples from this site are included in this study. Two of them are considered Neolithic, although have not been directly darted. A third sample which gave both abnormal archaeological context and abnormal results in subsequent analyses has been given the designation Iron Age, however without directly dating the sample it is difficult to accurately predict the age of a sample and hence the designation of Iron Age should not be considered firm (94). In consequence this sample was removed from the dataset. The two other samples are in rows 465-466 of Table S5.

United Kingdom

The Ness of Brodgar is a Late Neolithic complex on Mainland, Orkney, dating from circa 3500 to 2300 cal BC (95). The two samples included are from the large animal bone deposit in Structure 10 dating to around 2340–2200 cal BC (96) or 2565–2360 cal BC (96). The two samples, aBROD1-GBR (= NOB'09 1237 tRP Bag3 3331) and aBROD2-GBR (= NOB'09 1239 Bag3 3382) are in lines 485-486 of Table S5.
Snusgar is a Norse settlement at the Bay of Skaill, West Mainland, Orkney. Due to the dimensions of the hall and the assemblage, together with the place-name of the bay, the site has been interpreted as an estate center or skáli, which was probably subordinate in status to the Earldom estates (96, 97). The dataset includes two samples from a well-preserved longhouse on East Mound, which is one of two excavated settlement foci at the site: aORKN1-GB - SG10 TR5 2204 phase 3 - 10th-11th Century, and aORKN2-GB - SG10 TR5 2259 phase 5 - 11th-12th Century, which are in lines 472 and 470 of Table S5, respectively.
Sligeanach is a Bronze Age site on the island of South Uist, Western Isles, dated to 2200–1700 cal BC (98). One sample is included in this dataset, aSILG1-GBR, (= SL98 11.6.98 Context 18 TrenchA Mound3 1339), which is in line 467 of Table S5.
The Early Bronze Age-Early Iron Age (c. 2000-500 BC) site of Cladh Hallan in South Uist, Western Isles, is known for its exceptionally well preserved Late Bronze Age-Early Iron Age roundhouses c. 1050-500 BC (99). The huge artefact assemblage, including bronze and gold metalwork, attests to the inhabitants' prosperity and long-distance contacts. Five samples are included dating from Late Bronze Age to Early Iron Age contexts:
aHALL1-GBR = CH00 1414 House 401 Phase 11 (995-860 BC)
aHALL2-GBR = CH97 462 House 401 Phase 14 (755-595 BC)
aHALL3-GBR = CH00 1436 House 150 Phase 14 (755-595 BC)
aHALL4-GBR = CH01 2405 W Area Phase 9 (1085-965 BC)
aHALL5-GBR = CH01 2408 NE Area Phase 6 (1310-1080 BC)
Those samples are in lines 479-483 of Table S5.
Bornais is a multiperiod site located on South Uist in the Western Isles. Analysis of the site has dated a large amount of material to the mid-first millennium A.D (100). One sample is included in this dataset from a Late Iron Age midden dated to the 5th-6th century AD (aBORN1-GBR = B099 21/6/99 M1 Newtrench context 448) which is in line 475 of Table S5.
The Norse site of Cille Pheadair (Kilpheder) on the island of South Uist, Western Isles, is a single farmstead successively occupied and rebuilt in nine phases between c. AD 1000 and 1220 (101). The rich assemblage of pottery, ironwork, gold, and silver reveals that this longhouse's inhabitants had long-distance connections across the Viking world. Two samples are included in this dataset, aKILP2-GBR (= Kil'96 #091 Phase 8; AD 1140-1205) and aKILP3-GBR (= Kil'96 #303 Phase 7; AD 1105-1160), which are in lines 473-474 of Table S5.
Potterne is a late Bronze Age/Early Iron Age site in the village of Potterne, Wiltshire. Dates for the site range from 3430 ±110 to 2490 ±70 uncal BP; the majority of finds are attributed to the Late Bronze Age and Early Iron Age. The site contained a rich record of pottery, charred plants, and animal remains (102). One sample is included in this dataset, aPOTT2-GBR (= 1983.200 2624 2616-2624 Box 4088), which is in line 484 of Table S5.

Danebury is an Iron Age Hillfort, Hampshire, excavated in the 1970s and is considered a type-site for hillforts. Occupation of the site lasted approximately 500 years, beginning 2,500 years ago (103). Three samples are included in this dataset: aDANE1-GBR (= DA71 P78 10), aDANE2-GBR (= DA74 P658 4), and aDANE3-GBR (= DA71 P110 3) which are in lines 476-478 of Table S5.

The site of Hungate, York, has had continuous occupation for over 2000 years. One sample included in this study originates from Area H2, a medieval pit backfill that likely dates to the 11th Century. The sample is labelled aHUNG1-GBR (= 48302 Area H2) and is in line 471 of Table S5.

Ireland

Three samples were provided via a collaboration with Dr. Finbar McCormick. Two samples are from the Viking site of Fishamble Street, Dublin, and are among the most recent (ancient) samples included in this study, being approximately 900 years old (104). The third sample from Moynagh Crannog near Nobber in Co. Meath is an early medieval sample probably 7-9th century AD. These samples are in rows 487-489 of Table S5.

Malta

Tarxien is a late Neolithic temple site in Malta. A single sample included in this project is from the site and has not been directly dated. Three megalithic temples are at the site, dating to approximately 3,000 B.C.E. (105). This sample is in row 490 of Table S5.

Serbia

The site of Blagotin is in central Serbia within the village of Poljna, 26 km from the town of Trstenik. It is located on a gently sloping terrace, above an incised stream valley at the base of a mountain. Blagotin is a multi-period and stratigraphically complex site. It was initially inhabited during the Early Neolithic (Starčevo culture), reoccupied during the Eneolithic, and occupied again during the Early Iron Age. Each of the occupations only partially overlapped the other, resulting in both lateral displacement and vertical superposition of cultural stratigraphy (106). Recent radiocarbon dates place it among the earliest sites in the Starčevo culture – ca. 6100 cal BC (107, 108). In total 12 samples from the site were analyzed and directly dated as a part of this project, and it is confirmed to be the Early Neolithic. The samples from this site appear in rows 491-502 of Table S5.

The site of Bubanj is located on a low river terrace, near the confluence of the Nišava and the Južna Morava rivers, 5 km west of the town of Niš, in south-eastern Serbia. Bubanj is the eponymous site of the Central Balkan Eneolithic cultural traditions and is of great importance for the understanding of the development of prehistoric metal age societies in the region. It is a multi-layered site, with up to 3.5-m-thick cultural layers dating from the Early/Middle Neolithic, through the Eneolithic and Early Bronze Age, to the Early Iron Age. Altogether, the occupation covers a time frame of about 5000 years. The major portion of the cultural deposit is attributed to the Eneolithic (ca. 4500-2500 cal BC), which is represented by three successive settlements (109). The only sample from the site was directly dated to confirm its context and it is dated to the Early Eneolithic. This sample appears in row 503 of Table S5.

Israel

One sample came from the Israeli site of Tel Masos, which is a small tel located some 12 km east of the city of Beersheva in the northern Negev. The main occupation of the tel dates to the Chalcolithic and Iron Age I periods. The sample included in this project was directly dated to the Chalcolithic, mid-5th to 4th millennium BC. It is in row 524 of Table S5.

The site of Tel Miqne/Ekron is situated on the Israeli coastal plain ca. 35 km south-west of the city of Jerusalem. The main site occupation was in the Late Bronze Age II to the end of the Iron Age (ca. late 16th -

15th centuries to 7th /6th centuries BC) with scanty earlier Chalcolithic and Early Bronze Age remains (110). Four samples from the site were included in this project. They are in rows 520-523 of Table S5.

Tel Yoqne'am (sometimes referred to as Tel Yokneam) is located ca. 30 km inland and to the south of the city of Haifa. It was occupied from the Early Bronze Age (ca. 3150-2200 cal BC) to the Ottoman period i.e. 19th century AD (111). Three samples analyzed for this project are from the later layers of the site associated with the Islamic period or the Crusader occupation of the site, and as such have been classified as Medieval. One of the samples was directly dated and confirms that it is from these later, Medieval layers. Those samples appear in rows 518-519 and 525 of Table S5.

Shiqmim is a large village site, dating to the Chalcolithic period, that is located on the banks of the Beersheva Stream in the the northern Negev Desert, Israel. The site has four occupation strata spanning the time range 4500 to 3700 cal BC. Two samples from the site were included (112). They are in rows 526-527 of Table S5.

Türkiye

A single sample in this study is from the site of Çukuriçi Höyük, in western Türkiye. The site was used through both the Neolithic and the Chalcolithic periods. The sample from this site is considered to be from the middle of the Neolithic occupation of the site, approximately 8,500 years ago. Settlers diet consisted of both domesticated animals like cattle and sheep, and wild hunted animals such as hare and deer (113). This sample appears in row 545 of Table S5.

The site of Körtik Tepe, in Diyarbakır, in eastern Türkiye is one of the oldest included in this study, dating from 12,000 years before present. The site is from the pre-pottery Neolithic A and does not show signs of animal domestication (114-116). The site was later occupied during the Medieval period, and although some caution is advised, all samples were archaeologically dated to the early Neolithic. The two samples analyzed here appear in rows 628-629 of Table S5.

Our study also includes several samples from the Marmara region in western Türkiye. The samples are considered Neolithic, with two being directly dated, confirming that they are approximately 8,000 years old. The samples from this region appear in rows 528-532 and 541-542 of Table S5.

Menteşe Höyük in northwestern Türkiye is dated as Neolithic. The site shows evidence of domesticated animals, with the animal remains being skewed towards domestic species and away from hunted wild animals (*11*). None of the eight samples included in this project have been directly dated, however other samples from the site have been dated, confirming the Neolithic context. These specimens appear in rows 533-540 of Table S5.

Two samples included within this project are from the site of Suberde, a Neolithic site that is considered one of the earliest to show sheep management in central Anatolia. In initial studies, the site was thought to have been a hunter village but recent work suggests that the site represents an early central Anatolian location in which sheep were being managed (117). The large faunal assemblage that was extracted from the site is still being studied. The two samples in this project have not been directly dated and appear at rows 543 and 544 of Table S5.

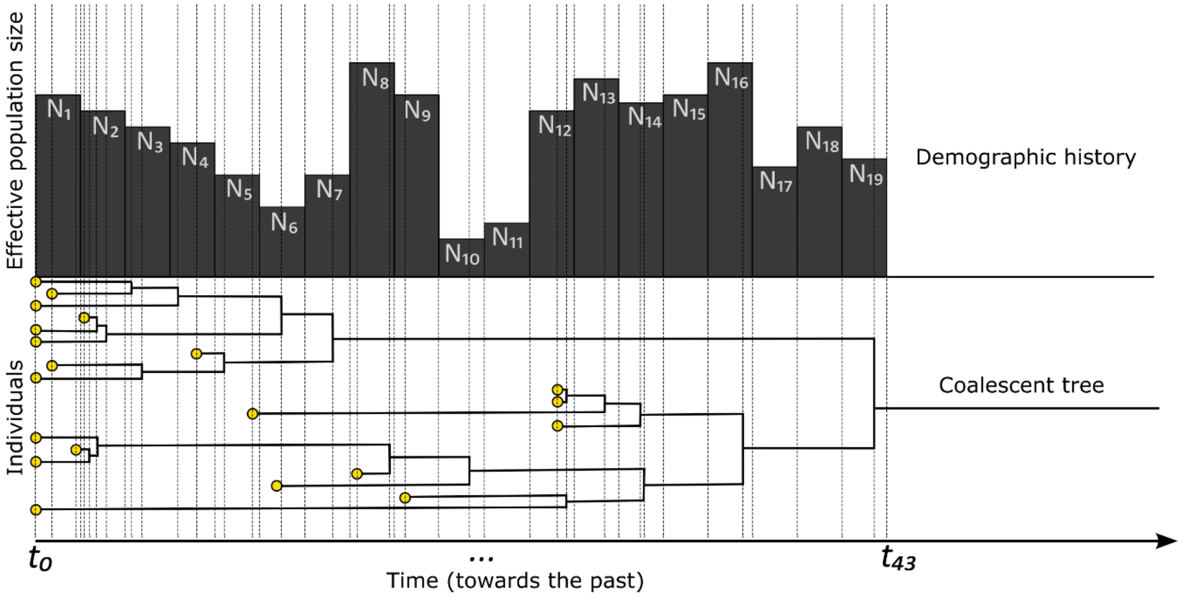

Fig. S1. Demographic inference from coalescent times. A direct probability modelling (an exponential probability density function) relates the effective population size to the inter-coalescent times. The computation is much facilitated by using stepwise demographic history i.e. a collection of constant size periods with fixed time boundaries (here the set $\{N_1, N_2, N_3, \ldots N_{19}\}$). The posterior probability of the demographic history is the prior probabilities times the product of every constant-size time period that is limited by any of these options: (i) a coalescent event, (ii) an appearance of an ancient sample or migrant, or (iii) a population size change. In the figure, there are 43 of those periods, indicated by vertical dotted lines.

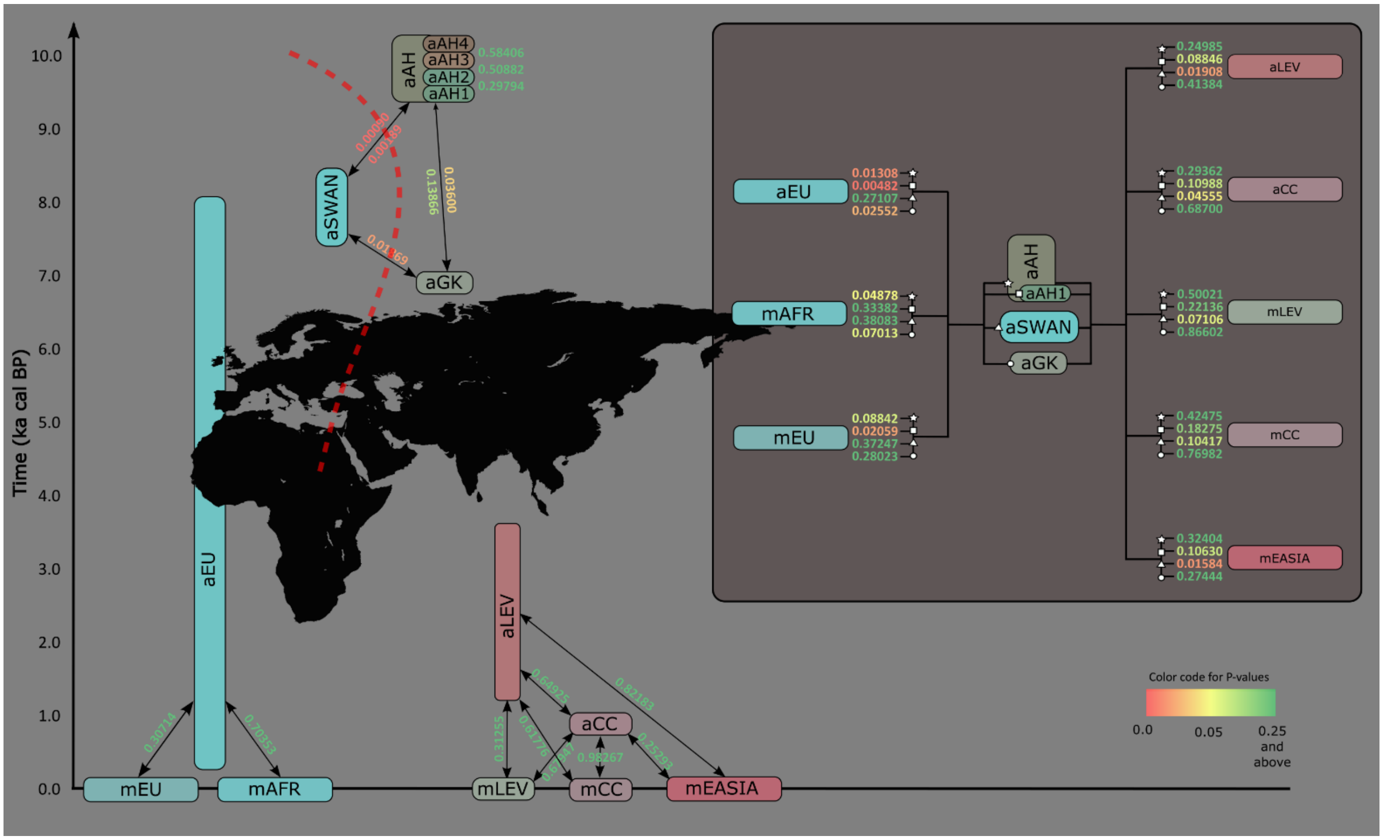

Fig. S2. Graphical summary of temporal tests. Values upon black lines show the P-value of a temporal test of haplogroup frequencies (Bayesian test with $\boldsymbol{N_e} = 10^4$) between the two samples at the two extremes of the line, pointed by the arrow tips. They were extracted from **Table S6**. The colors of those values only indicate the degree of statistical significance (color code at bottom right). The box (top right) shows the P-values of the comparisons between pairs made of an ancient Anatolian sample and a non-Anatolian one. The four values correspond to the comparison of the given sample (e.g. aEU = ancient Europe) and the four Anatolian samples at the center, namely aAH (Aşıklı Höyük, full sample), aAH1 (Aşıklı Höyük youngest layers, 2A-C), aSWAN (Neolithic southwestern Anatolia including Suberde, Çukuriçi, Menteşe and Marmara regions), and aGK (Güvercinkayası). For instance, the four values in front of mAFR (modern Africa), correspond to the comparisons: modern Africa-Aşıklı Höyük (0.048), modern Africa-Aşıklı Höyük layers 2A-C (0.33), etc. Samples codes follow Table 1 and S5.

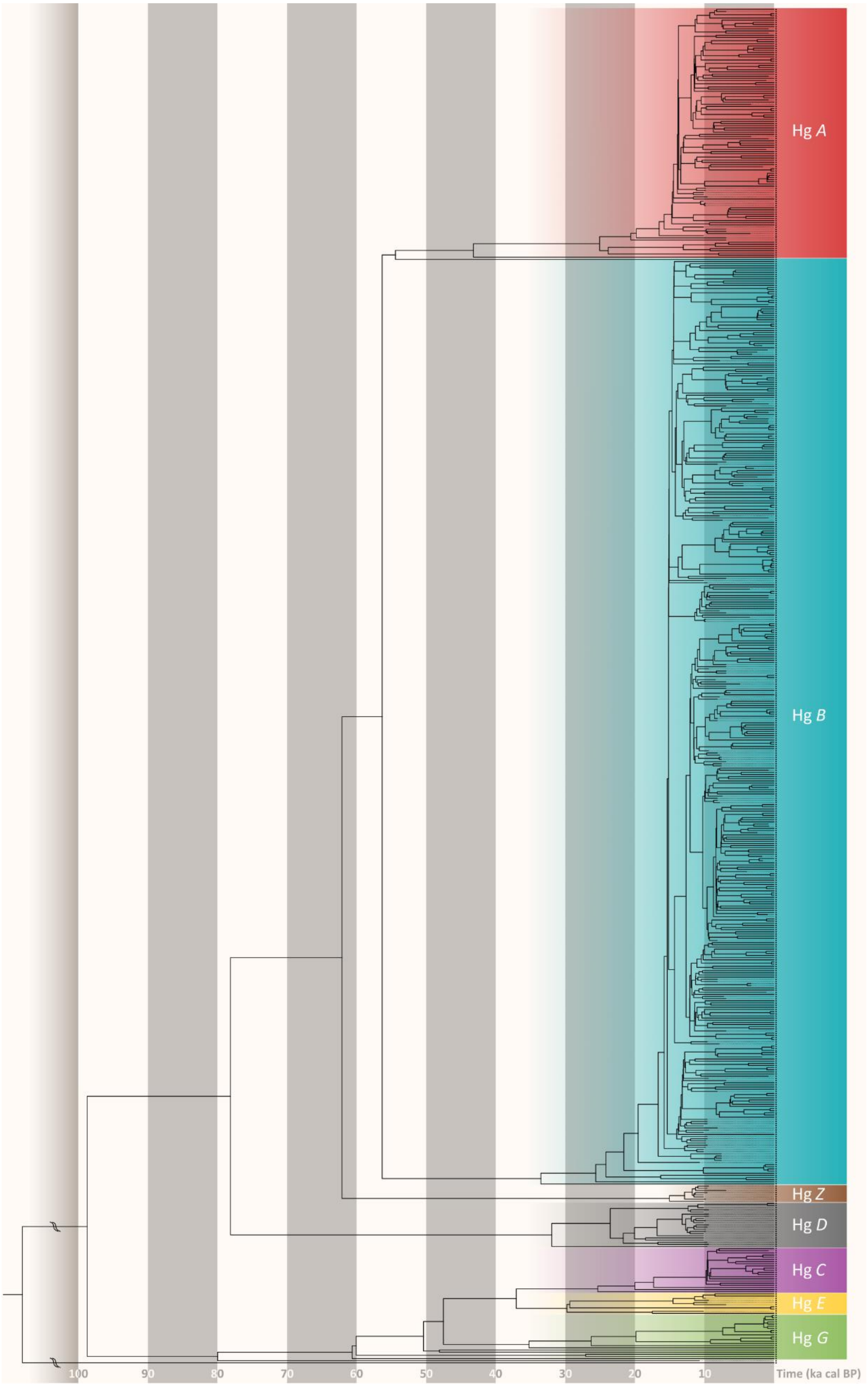

Fig. S3. Phylogenetic reconstruction of all 629 mitogenomes. The labels have been removed (see Figs. S4-S7 to identify individual labels and geographic origin). Hg is short for haplogroup.

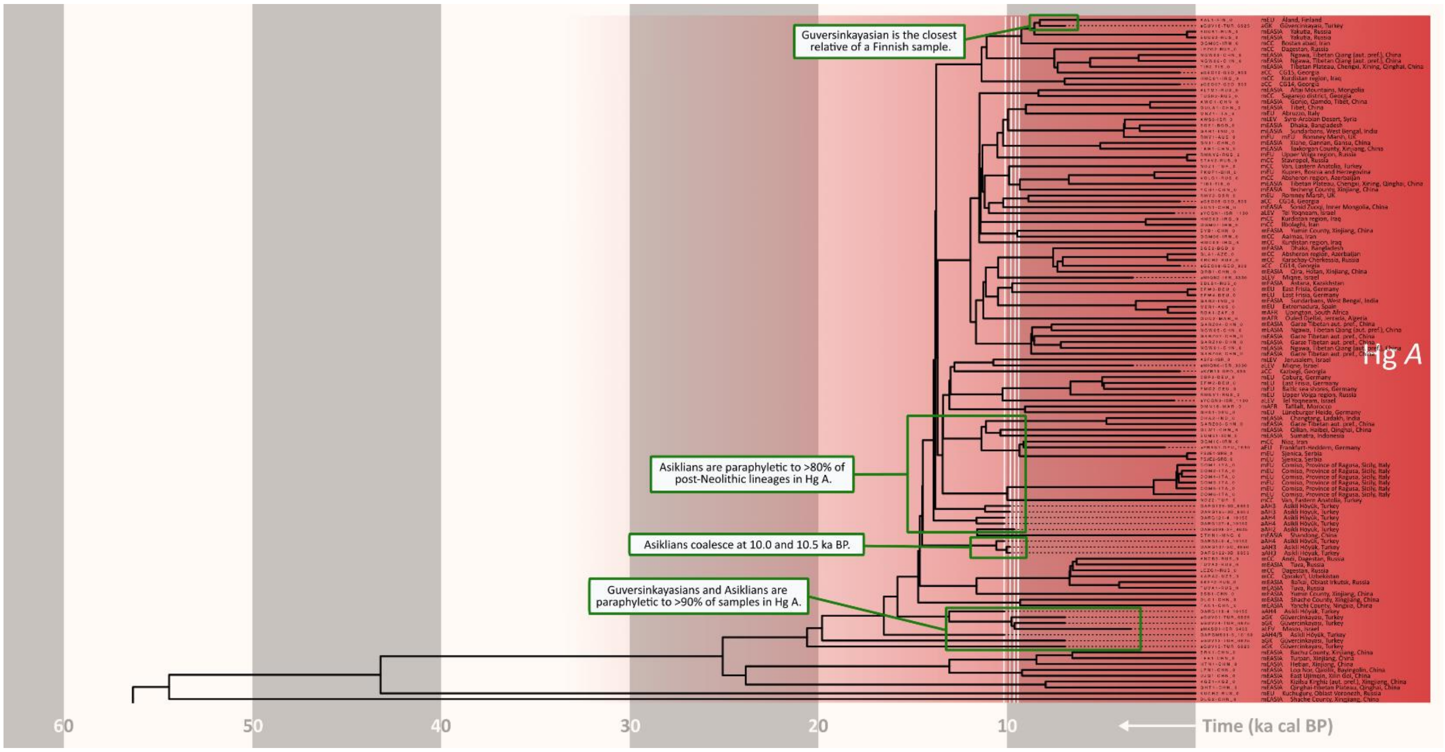

Fig. S4. Phylogenetic reconstruction, partial. This corresponds to an expanded view, with labels and comments, of Fig. S3, top. White lines indicate the medium time of archaeological layers 2A-C (youngest), 2D-J, 3A-E, & 4A-C (oldest) of Asikli Höyük.

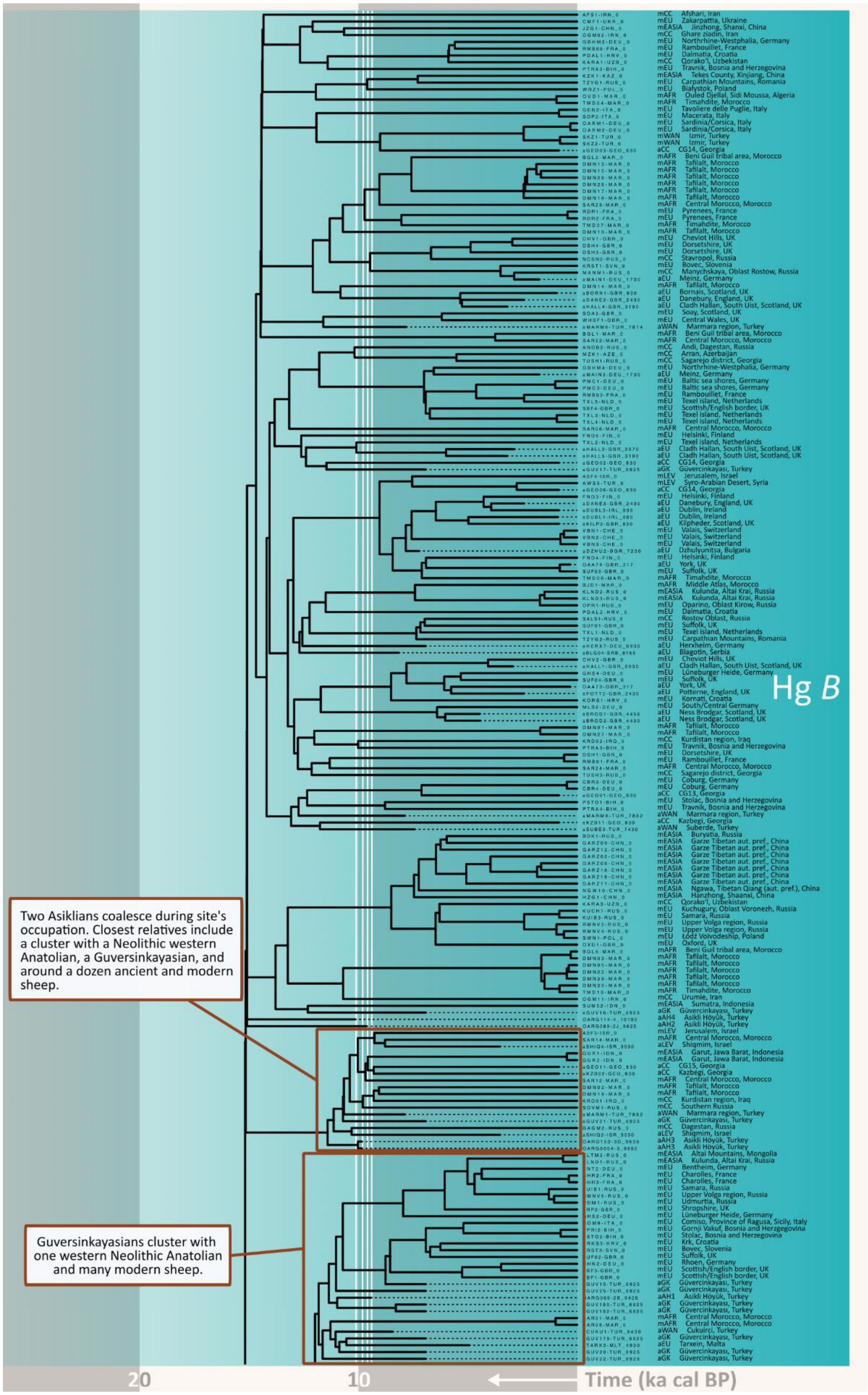

Fig. S5. Phylogenetic reconstruction, partial. This corresponds to an expanded view, with labels and comments, of Fig. S3, middle-top. White lines indicate the medium time of archaeological layers 2A-C (youngest), 2D-J, 3A-E, & 4A-C (oldest) of Asikli Höyük.

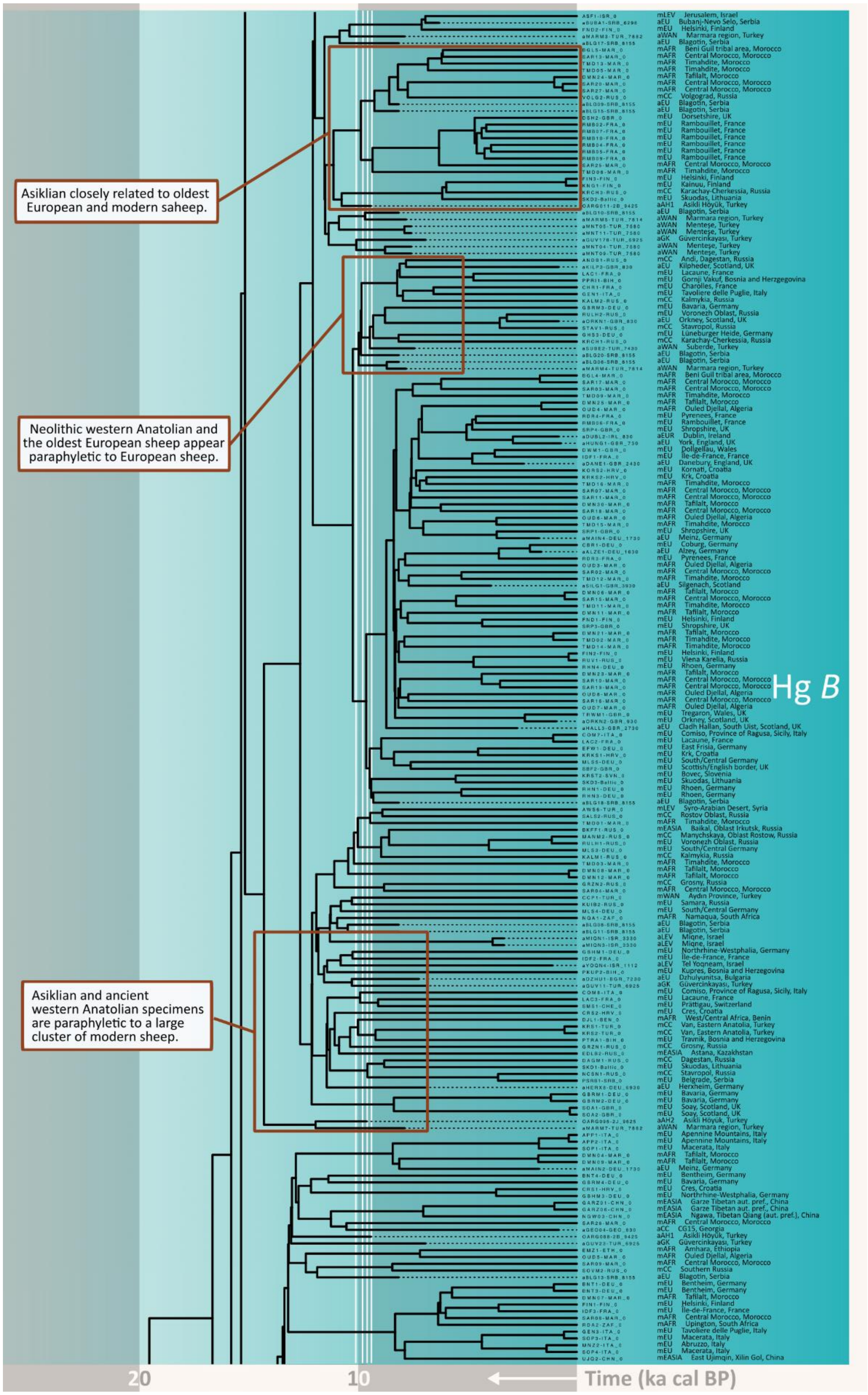

Fig. S6. Phylogenetic reconstruction, partial. This corresponds to an expanded view, with labels and comments, of Fig. S3, middle-bottom. White lines indicate the medium time of archaeological layers 2A-C (youngest), 2D-J, 3A-E, & 4A-C (oldest) of Asikli Höyük.

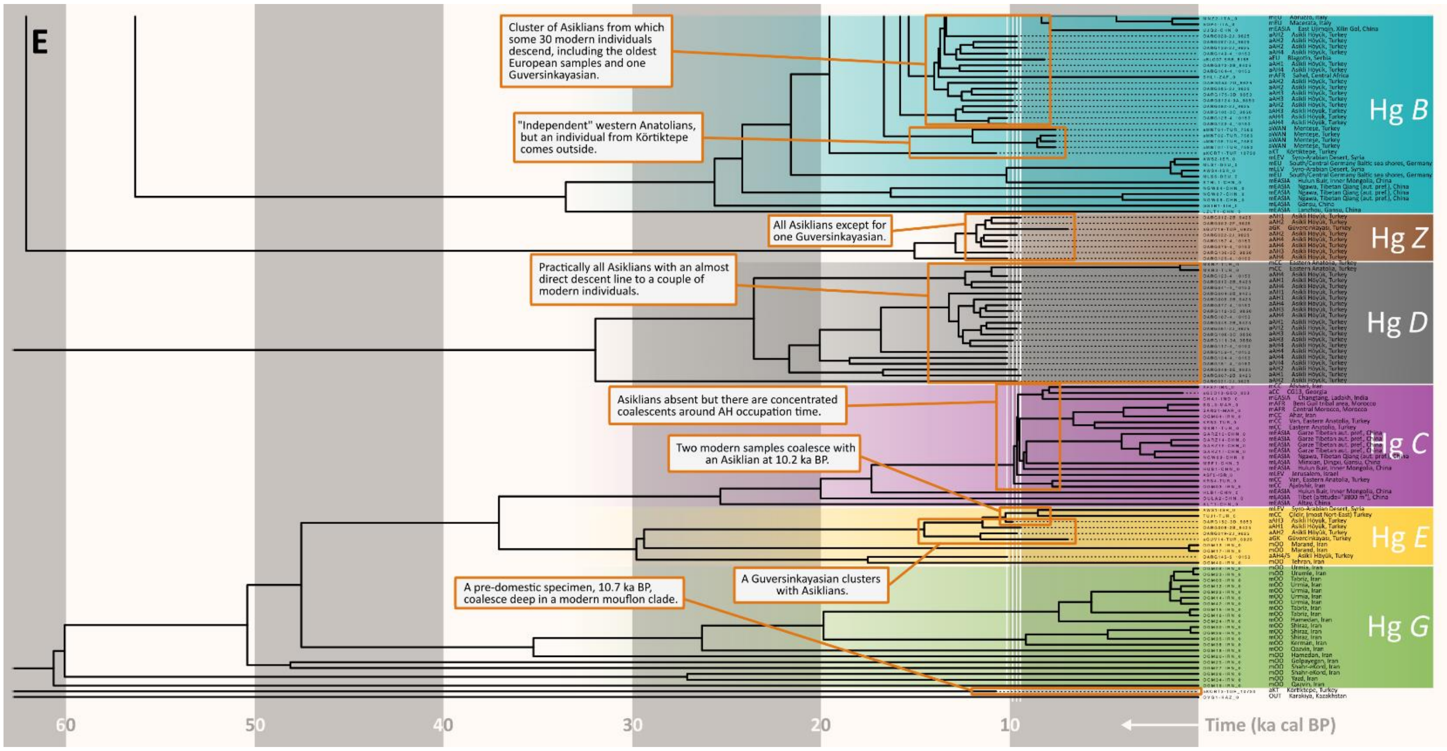

Fig. S7. Phylogenetic reconstruction, partial. This corresponds to an expanded view, with labels and comments, of Fig. S3, bottom. White lines indicate the medium time of archaeological layers 2A-C (youngest), 2D-J, 3A-E, & 4A-C (oldest) of Asikli Höyük.

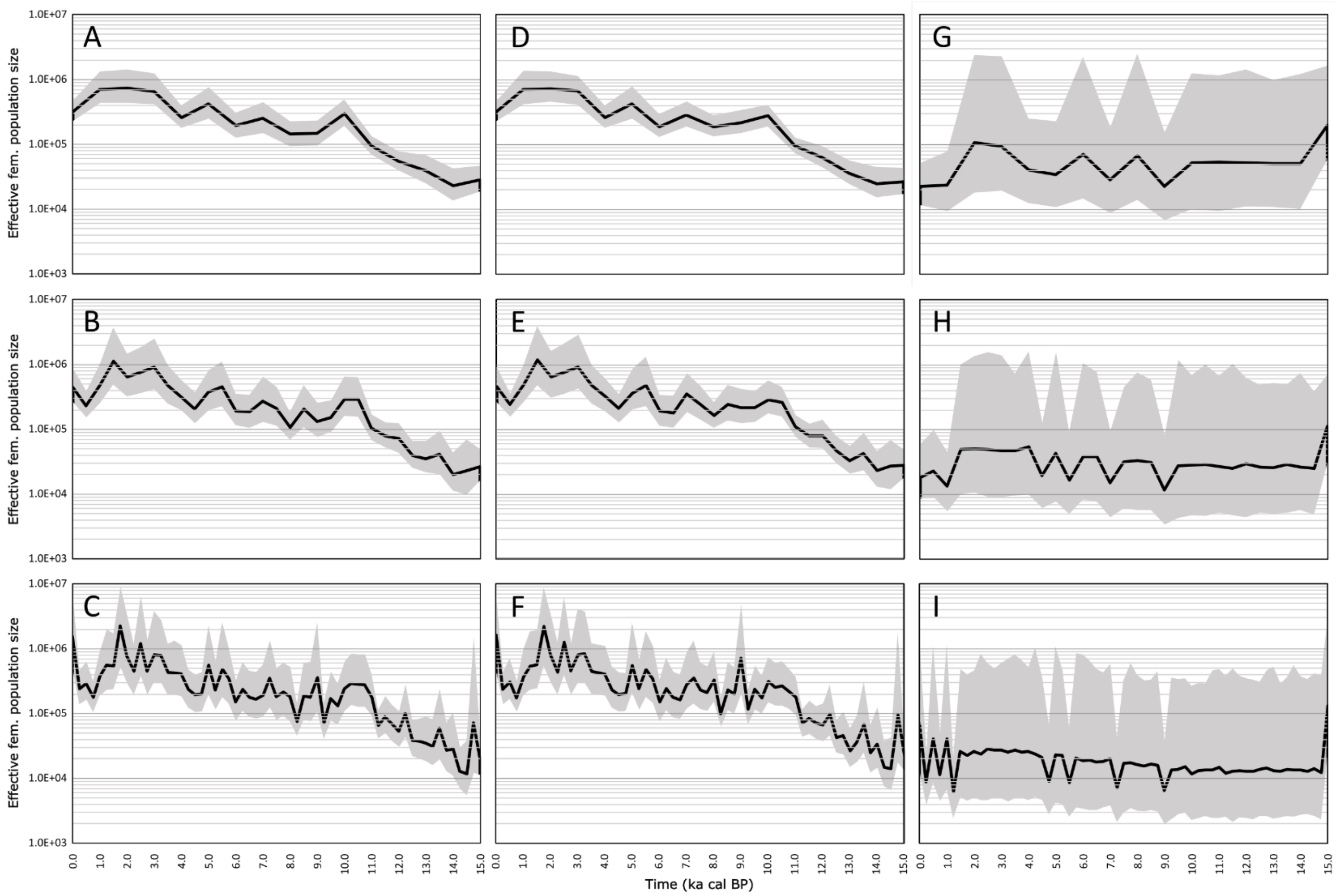

Fig. S8. Demographic histories inferred from coalescent times. The histories were made with 16 (top, **(A)**/**(D)**/**(G)**), 32 (middle, **(B)**/**(E)**/**(H)**), or 64 (bottom, **(C)**/**(F)**/**(I)**) time bins. More time bins means that the inferred history has more resolution but more statistical noise while less time bins is more statistically reliable but misses detail. Panels **(A)-(C)** show the plots obtained analyzing samples of modern and ancient Europe, and the Neolitihc sites Aşıklı Höyük, and Körtik Tepe. Panels **(D)-(F)** show plots made with the samples of the group **(A)-(C)** plus samples from Neolithic southwestern Anatolia (including Suberde, Çukuriçi, Menteşe, and Marmara regions) and Chalcolithic Güvercinkayası. Panels **(G)-(I)** show the plot obtained with modern mouflon samples. The histories and their 95% HDP region (in grey) were obtained from a MCMC procedure described in Supplementary Text S7.

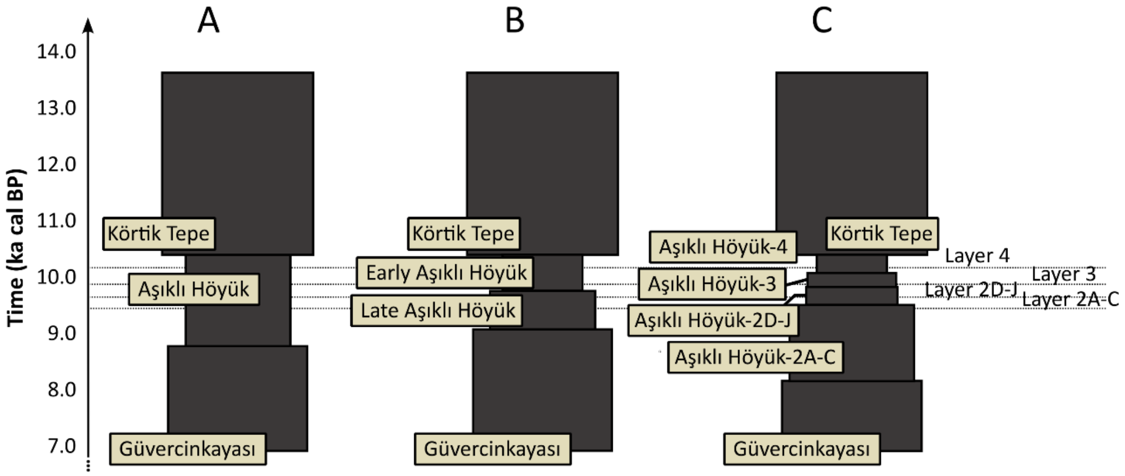

Fig. S9. Alternative models of the ABC model choice analysis phase 1. The three showed scenarios correspond to three alternative models representing the demographic history of Aşıklı Höyük: **(A)** a model with no change during the occupation of the site; **(B)** a model with two demographic stages during the occupation of the site (separated around 9.7 ka BP); and **(C)** a model with four stages, each containing one of the four archaeological layers sampled in this study (named at right hand).

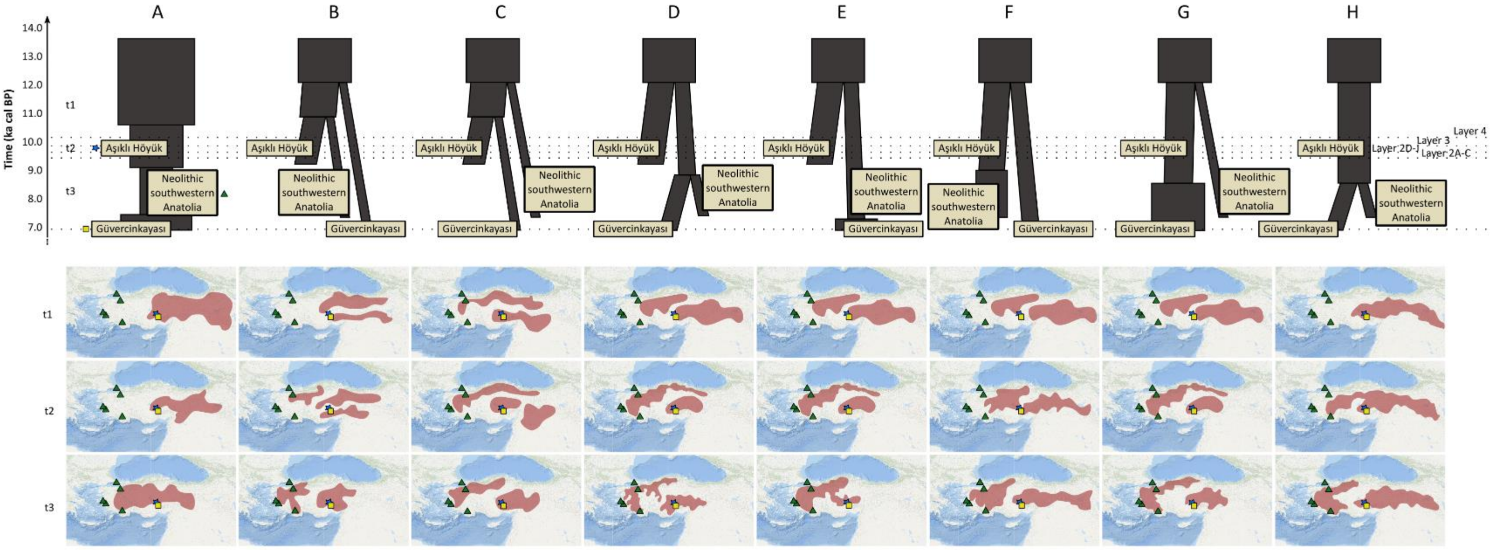

Fig. S10. Alternative models of the ABC model choice analysis phase 2. The scenarios represent a non-exhaustive but sensitive set of scenarios modelling the relationships among the populations to which Aşıklı Höyük, Güvercinkayası and Neolithic southwestern Anatolia belonged to. The mini-map series at the bottom show possible spatial distributions (scarlet shades) of the sampled sheep populations at three time points (*t*1, *t*2, and *t*3) to help visualizing the abstract models in a geographical context (by no means they correspond to accurate or inferred distributions). The star, triangle, and square icons in them indicate the locations of Aşıklı Höyük, Güvercinkayası, and the sites of Neolithic southwestern Anatolia. For a more realistic assessment, consider that unsampled populations may have existed and that each shade could be constituted by a network of human communities with their respective sheep herds.

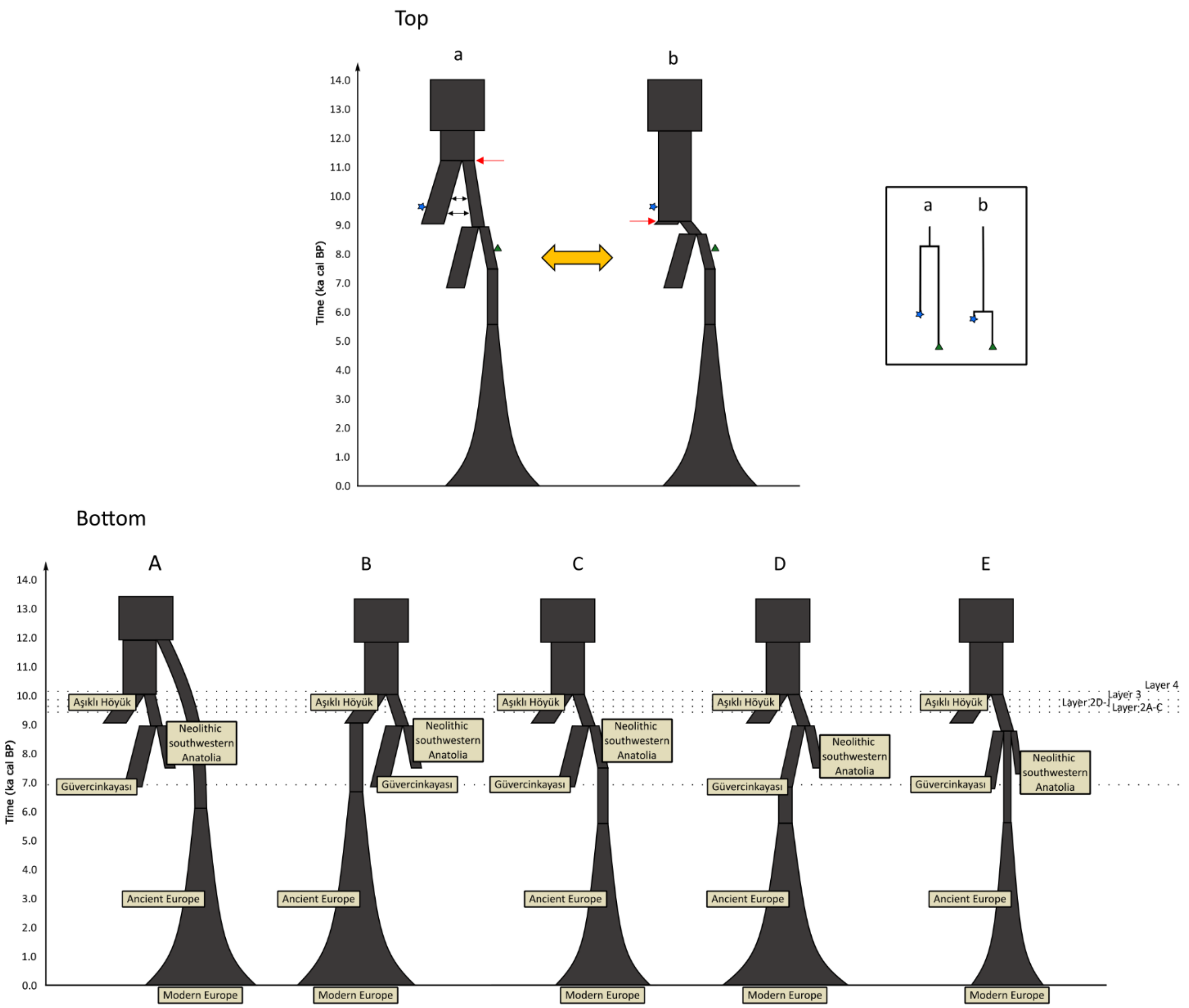

Fig. S11. Competing models of the ABC model choice analysis phase 3. The scenarios constitute alternative models of the ancestry relationship between the European population and the (sampled) ancient Anatolian populations. The models **(A)-(E)** were constructed considering the best model selection of our ABC analysis of phase 2 (Fig. S10) and considering five alternative ancestry relationships of Anatolian populations and the European sheep population (both ancient and modern). Because there were two models selected in the phase 2 (D and H on Fig. S10), we used a flexible base model (top chart) that approaches both models depending on the value of a single parameter: the split time of Aşıklı Höyük and the ancestor of Güvercinkayası and Neolithic southwestern Anatolia (indicated with a red arrow). Notice that, when this time is in the Epipaleolithic, the ancestor of other Anatolian populations is a sister population of Aşıklı Höyük while if the value is younger than the occupation of Aşıklı Höyük, then the other populations descend directly from Aşıklı Höyük. The little asterisk and triangle shapes represent samples from Aşıklı Höyük and Neolithic southwestern Anatolia, respectively, and the scheme in the box shows the expected coalescent diagrams of those samples under both models (identified as 'a' and 'b'). Interestingly, our phylogenetic reconstruction contained both types of coalescences among samples from Aşıklı Höyük and Neolithic southwestern Anatolia.

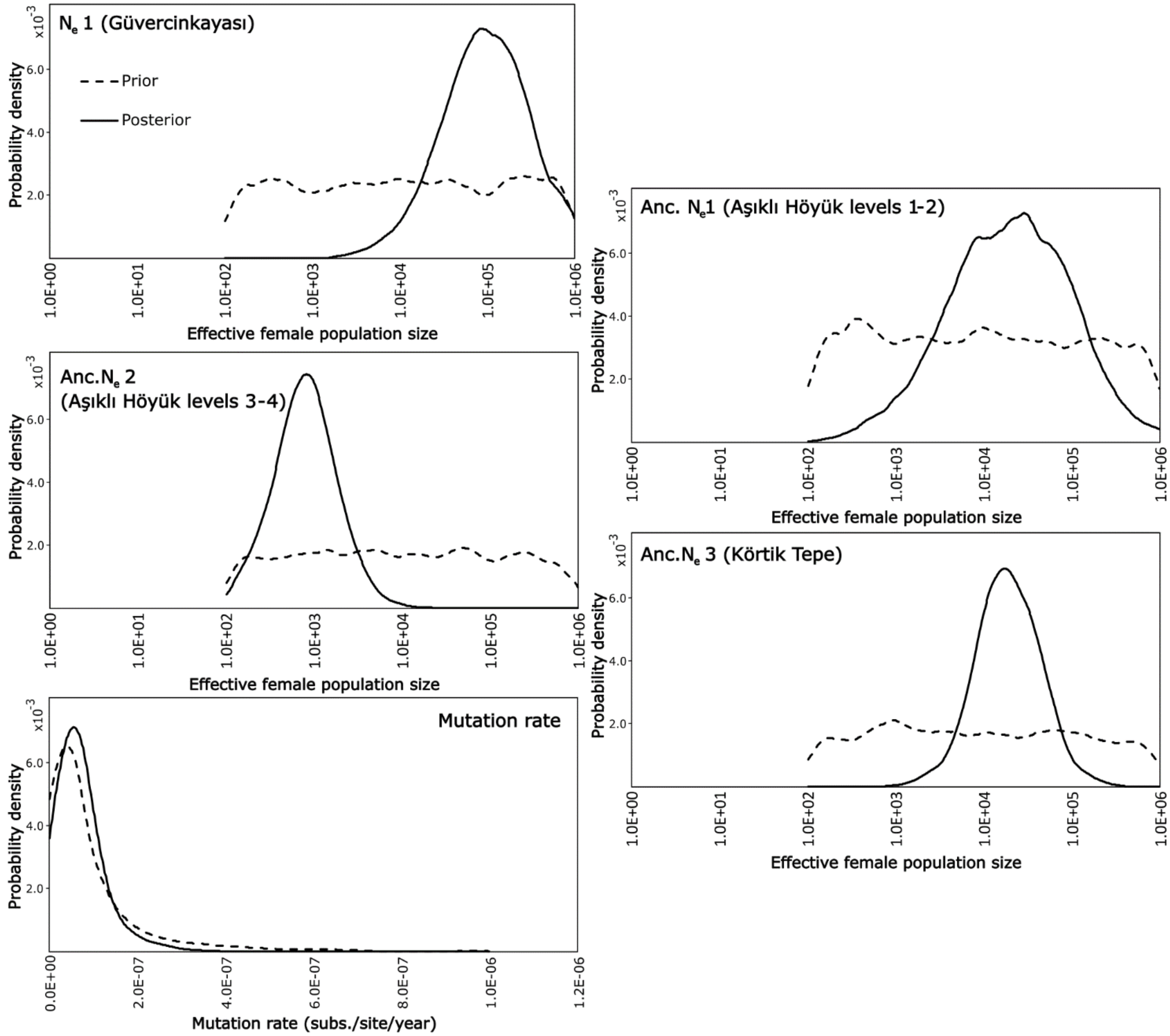

Fig. S12. Posterior distributions from the ABC analyses phase 1. The posteriors correspond only to the effective population sizes (N_e) and mutation rate of the best supported model (the two-period model) in the model choice analysis phase 1.

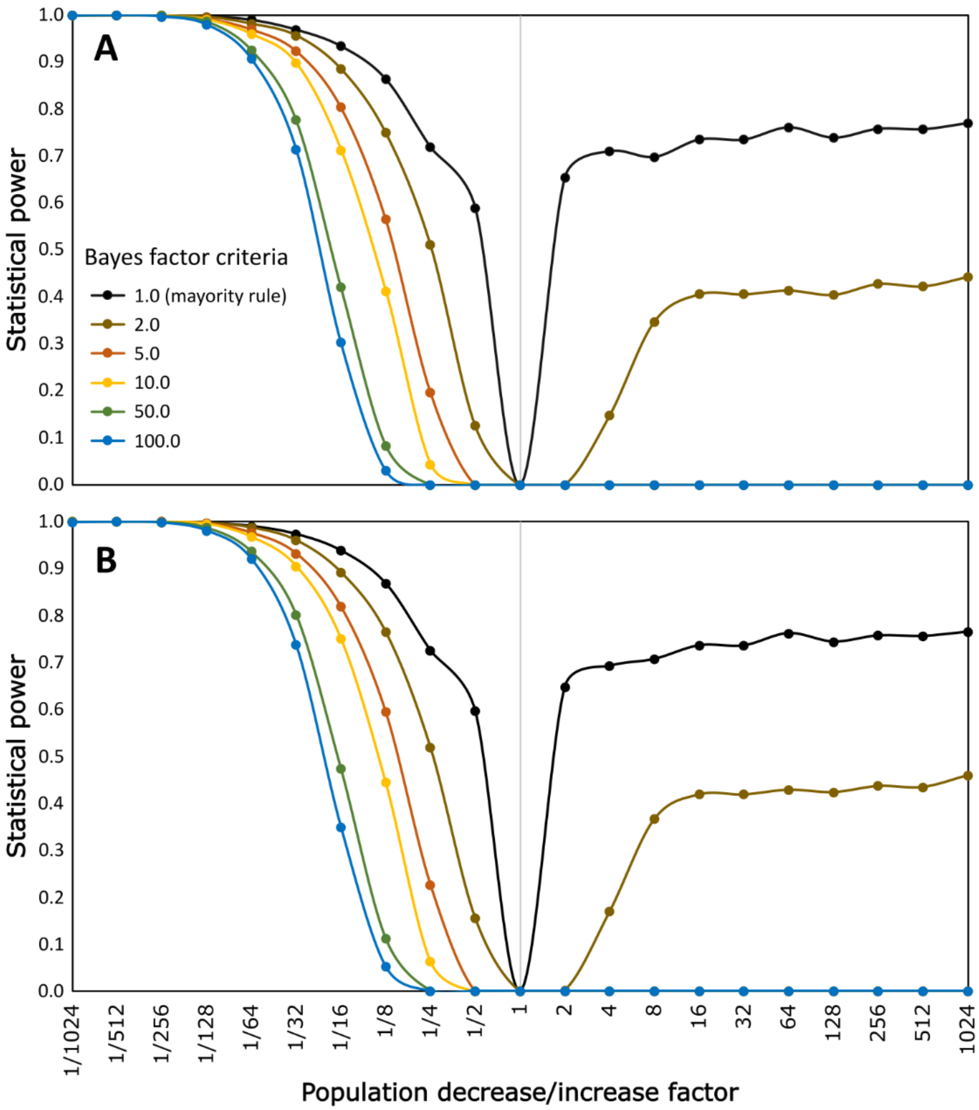

Fig. S13. Statistical power for bottleneck detection at Asikli-Hoyuk. The test consisted in a model choice analysis by approximate Bayesian computation (ABC) with two competing models: a model without a change and a model with a population reduction/increase by a factor of 2, 4, 8, 16, 32, 64, 128, 256, 512, or 1024. The charts resulted from estimating the model likelihoods directly from: **(A)** acceptance ratios; or **(B)** with a distance-weighting adjustment by means of an Epanechnikov kernel.

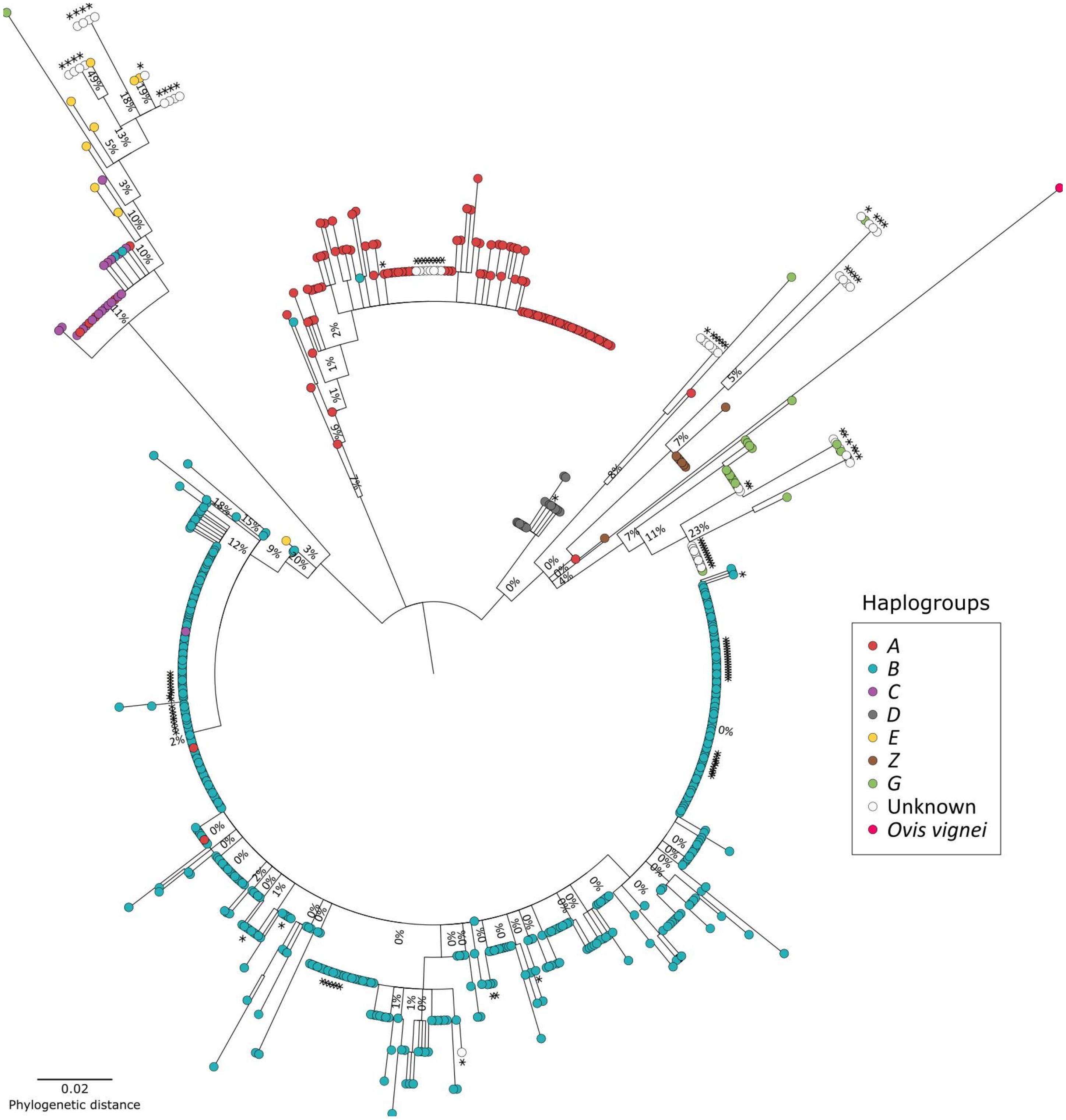

Fig. S14. Phylogeny of the pooled dataset. The phylogenetic tree was obtained from a composite dataset including 711 sequences, from which 629 came from downsampling our dataset and adding 82 sequences of a 120 bp fragment of control region published in (41). The tree was inferred by using the Maximum Likelihood method and General Time Reversible model (+G, parameter = 0.7818, +I, 35.00% sites) and 100 bootstrap replicates (branches with <50% support were collapsed). Evolutionary analyses were conducted in MEGA X (65). The asterisks indicate the samples published in (41) and the color correspond to the assignation given in this study. Notice the enrichment of published sequences in the clade where all haplogroup Z are located making it possible that those unidentified published sequences belong to haplogroup Z.

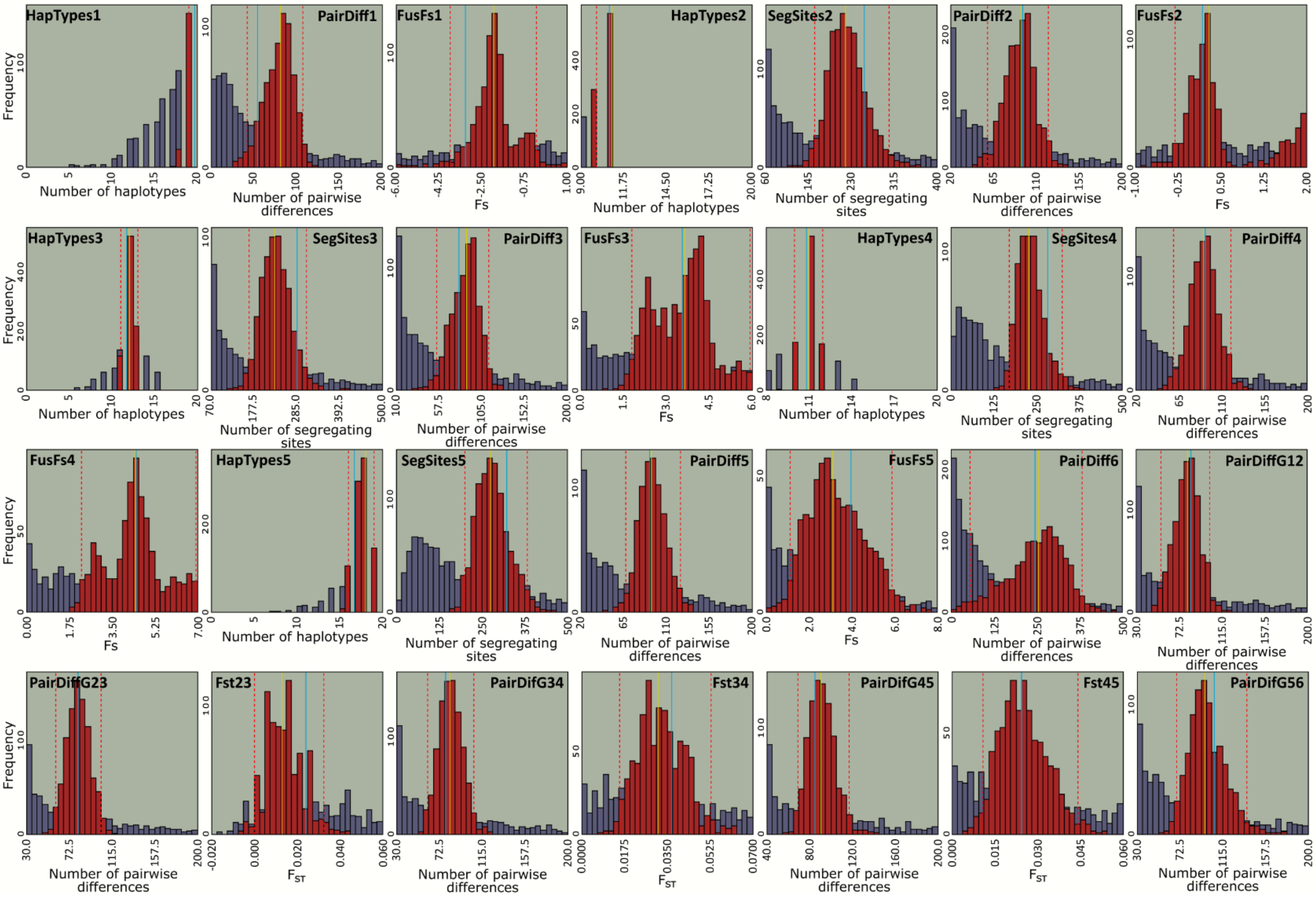

Fig. S15. Predictive distributions of summary statistics, ABC phase 1. The predictive distributions of the analysis of 28 summary statistics ("mid set' in Table S8) are shown in red. The distributions in purple correspond to those before rejection. The vertical lines correspond to the empirical value (blue) and the median (yellow). For further information see Tables S7 and S8.

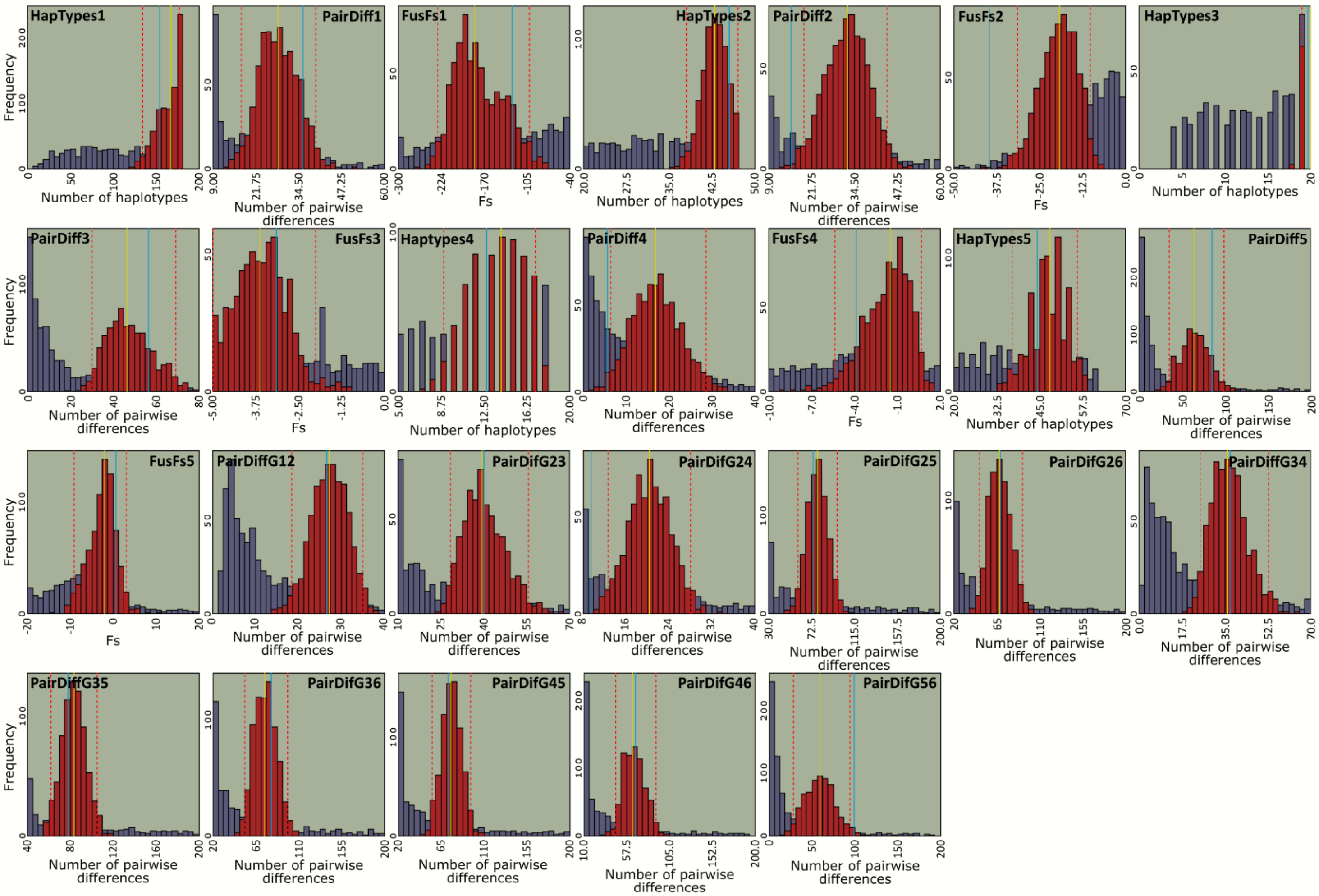

Fig. S16. Predictive distributions of summary statistics, ABC phase 4. The predictive distributions of 26 summary statistics (“26-set-F” in Table S12) are shown in red while the purple distributions behind correspond to those before rejection. The vertical lines correspond to the empirical value (blue) and the median (yellow). For further information see Tables S7 and S12.

Table S1. Composition of animal remains at Aşıklı Höyük. Contingency chi-square ($\chi 2$) test of the composition of *Ovis sp.*, *Capra sp.*, and other animal remains at Aşıklı Höyük. The tests were performed between consecutive pairs of layers, so the statistic value and the P-value appear in between the rows of the compared layers. The bottom row shows the statistic value and the P-value of the test performed over all layers. The information was taken from (8, 24).

Layer	*Ovis*	*Capra*	Rest	*n*	$\chi 2$	P-value
Phase 2A-C	0.67455	0.19473	0.13071	12501		
					345.20	< 0.00001
Phase 2D-J	0.68211	0.13707	0.18082	37109		
					364.15	< 0.00001
Phase 3A-E	0.57967	0.09550	0.32483	2863		
					456.26	< 0.00001
Phase 4A-C	0.36586	0.13698	0.49716	14619		
				Overall	7249.3	< 0.00001

Table S2. Size of sheep carcasses at Aşıklı Höyük. Two-sample t-test for unequal variances applied to the estimated length of sheep carcasses at Aşıklı Höyük. The tests were performed between consecutive pairs of layers, so the statistic values, degrees of freedom (d.f.), and P-values appear in between the rows of the compared layers. The information was taken from (24).

Layer	Mean	Variance	*n*	*t*	d.f.	P-value (one-tail)	P-value (two-tail)
Phase 2A-C	20.5638	1.3758	221				
				2.6250	410	0.00449	0.00899
Phase 2D-J	20.3177	1.3472	525				
				0.1496	79	0.44643	0.89285
Phase 3A-E	20.2927	2.2018	69				
				-2.8626	151	0.00240	0.00480
Phase 4A-C	20.9740	2.1701	96				

Table S3. Joint pathologies at Aşıklı Höyük. Two-sample t-test for unequal variances applied to the classification of the severity of joint pathologies (1=nonpathological, 4=maximum severity) of sheep remains at Aşıklı Höyük. The tests were performed between pairs of layers, so the statistic values, degrees of freedom (d.f.), and P-values appear in between the rows of the compared layers. The information was taken from (24, 25).

Layer	Mean	Variance	*n*	*t*	d.f.	P-value (one-tail)	P-value (two-tail)
Phase 2A-C	1.7840	1.1210	287				
				-1.1079	617	0.13417	0.26833
Phase 2D-J	1.8763	1.1329	372				
				0.3932	101	0.34749	0.69498
Phase 3A-E	1.8219	1.1762	73				
				1.9750	129	0.02520	0.05041
Phase 4A-C	1.4746	0.8743	59				

Table S4. Origin of the samples included in this study. The origin of Anatolian ancient samples is indicated specifically as archaeological site or region whereas samples outside southwestern Asia are only indicated at regional or continental scale due to their more scattered presence. Samples indicated as published include mitogenomes extracted from whole genome-sequencing data available in public databases and mitogenomes available in the GenBank. See further details in Table S5.

Origin	Type	*n*	This Study	Published	
				WGS	mtDNA
Europe	Ancient	47	45	2	
Neolithic southwestern Anatolia	Ancient	18	18		
Caucasus	Ancient	14	14		
Levant	Ancient	10	10		
Asikli Höyük	Ancient	62	62		
Güvercinkayası	Ancient	20	20		
Körkit Tepe	Ancient	2	2		
Africa	Modern	94		92	2
Europe	Modern	177	107	22	48
Anatolia	Modern	3		3	
Caucasus	Modern	59	28	13	18
Levant	Modern	11		5	6
Eastern Asia	Modern	87	12	13	62
Asiatic mouflon (*O. gmelini*)	Modern	24		24	
Outgroup (*O. vignei*)	Modern	1			1
Total	Ancient	173	171	2	0
Total	Modern	456	147	172	137
Overall		629			

Table S5. List of samples included in this study. This table show the full list of the 629 samples analyzed in this study along with their label, accession number, species, haplogroup, and sample code indicating the population in which they were grouped for analysis. The list also shows the estimated age and location data including coordinates and geographical origin that included each of the 163 localities of modern sheep and 33 localities for ancient specimens. The column "reference" also provides the source of the samples indicating the study where they were first published (name-date for easier identification) or whether they were sequenced *de novo* in this study, or retrieved from the sequence read archive (SRA: available at https://www.ncbi.nlm.nih.gov/sra). The alignment containing the full mitogenomic dataset is available at: 10.6084/m9.figshare.24552790.

#	ID	Accession ID	Species	Origin	Age	lat	long	Reference	Hg	Sample Code
1	OVG1-KAZ	HM236186.1	*Ovis vignei*	Karakiya, Kazakhstan	0	42.99	54.55	(31)	U	OUT
2	OGM08-IRN	ERS154529	*Ovis gmelini*	Urmia, Iran	0	37.485	45.653	SRA	G	mOO
3	OGM09-IRN	ERS154532	*Ovis gmelini*	Tabriz, Iran	0	37.855	46.570	SRA	G	mOO
4	OGM12-IRN	ERS154530	*Ovis gmelini*	Urmia, Iran	0	37.485	45.653	SRA	G	mOO
5	OGM13-IRN	ERS154526	*Ovis gmelini*	Marand, Iran	0	38.932	45.385	SRA	E	mOO
6	OGM14-IRN	ERS154527	*Ovis gmelini*	Urmia, Iran	0	37.481	45.628	SRA	G	mOO
7	OGM15-IRN	ERS154533	*Ovis gmelini*	Tabriz, Iran	0	38.769	46.147	SRA	G	mOO
8	OGM16-IRN	ERS154531	*Ovis gmelini*	Tabriz, Iran	0	37.747	46.393	SRA	G	mOO
9	OGM17-IRN	ERS419579	*Ovis gmelini*	Marand, Iran	0	38.746	45.458	SRA	E	mOO
10	OGM18-IRN	ERS419580	*Ovis gmelini*	Qazvin, Iran	0	36.611	49.117	SRA	G	mOO
11	OGM19-IRN	ERS239062	*Ovis gmelini*	Qazvin, Iran	0	36.721	49.321	SRA	G	mOO
12	OGM20-IRN	ERS239061	*Ovis gmelini*	Hamedan, Iran	0	34.643	48.622	SRA	G	mOO
13	OGM22-IRN	ERS239058	*Ovis gmelini*	Urmia, Iran	0	37.485	45.599	SRA	G	mOO
14	OGM23-IRN	ERS239059	*Ovis gmelini*	Urumie, Iran	0	37.487	45.637	SRA	G	mOO
15	OGM24-IRN	ERS239060	*Ovis gmelini*	Hamedan, Iran	0	34.705	48.863	SRA	G	mOO
16	OGM25-IRN	ERS239063	*Ovis gmelini*	Golpayegan, Iran	0	33.697	50.804	SRA	G	mOO
17	OGM26-IRN	SRR11657635	*Ovis gmelini*	Shahr-eKord, Iran	0	32.230	50.470	(36)	G	mOO
18	OGM27-IRN	SRR11657636	*Ovis gmelini*	Shahr-eKord, Iran	0	32.230	50.470	(36)	G	mOO
19	OGM30-IRN	SRR11657501	*Ovis gmelini*	Shiraz, Iran	0	29.587	52.527	(36)	G	mOO
20	OGM34-IRN	SRR11657505	*Ovis gmelini*	Yazd, Iran	0	31.880	54.357	(36)	G	mOO
21	OGM35-IRN	SRR11657506	*Ovis gmelini*	Shiraz, Iran	0	29.587	52.527	(36)	G	mOO
22	OGM36-IRN	SRR11657507	*Ovis gmelini*	Shiraz, Iran	0	29.587	52.527	(36)	G	mOO
23	OGM38-IRN	SRR11657639	*Ovis gmelini*	Kerman, Iran	0	30.286	57.079	(36)	G	mOO
24	OGM40-IRN	SRR11657641	*Ovis gmelini*	Tehran, Iran	0	35.580	51.447	(36)	E	mOO
25	OGM42-IRN	ERS154528	*Ovis gmelini*	Urmia, Iran	0	37.490	45.599	SRA	G	mOO
26	SKD1-Baltic	24552790***	*Ovis aries*	Skuodas, Lithuania	0	56.27	21.52	This study, PCR	B	mEU
27	SKD2-Baltic	24552790***	*Ovis aries*	Skuodas, Lithuania	0	56.27	21.52	This study, PCR	B	mEU
28	SKD3-Baltic	24552790***	*Ovis aries*	Skuodas, Lithuania	0	56.27	21.52	This study, PCR	B	mEU
29	PKUP1-BIH	24552790***	*Ovis aries*	Kupres, Bosnia and Herzegovina	0	43.99	17.28	This study, PCR	A	mEU
30	PKUP2-BIH	24552790***	*Ovis aries*	Kupres, Bosnia and Herzegovina	0	43.99	17.28	This study, PCR	B	mEU
31	PPRI1-BIH	24552790***	*Ovis aries*	Gornji Vakuf, Bosnia and Herzgegovina	0	43.96	17.68	This study, PCR	B	mEU
32	PPRI2-BIH	24552790***	*Ovis aries*	Gornji Vakuf, Bosnia and Herzgegovina	0	43.96	17.68	This study, PCR	B	mEU
33	PSTO1-BIH	24552790***	*Ovis aries*	Stolac, Bosnia and Herzegovina	0	43.09	17.96	This study, PCR	B	mEU
34	PSTO2-BIH	24552790***	*Ovis aries*	Stolac, Bosnia and Herzegovina	0	43.09	17.96	This study, PCR	B	mEU
35	PTRA1-BIH	24552790***	*Ovis aries*	Travnik, Bosnia and Herzegovina	0	44.23	17.66	This study, PCR	B	mEU
36	PTRA2-BIH	24552790***	*Ovis aries*	Travnik, Bosnia and Herzegovina	0	44.23	17.66	This study, PCR	B	mEU
37	PTRA3-BIH	24552790***	*Ovis aries*	Travnik, Bosnia and Herzegovina	0	44.23	17.66	This study, PCR	B	mEU
38	PTRA4-BIH	24552790***	*Ovis aries*	Travnik, Bosnia and Herzegovina	0	44.23	17.66	This study, PCR	B	mEU
39	SMS1-CHE	SRS335692	*Ovis aries*	Prättigau, Switzerland	0	46.95	9.75	SRA	B	mEU
40	VBN1-CHE	24552790***	*Ovis aries*	Valais, Switzerland	0	46.20	7.60	This study, PCR	B	mEU
41	VBN2-CHE	24552790***	*Ovis aries*	Valais, Switzerland	0	46.20	7.60	This study, PCR	B	mEU
42	VBN3-CHE	24552790***	*Ovis aries*	Valais, Switzerland	0	46.20	7.60	This study, PCR	B	mEU

43	CRS1-HRV	24552790***	*Ovis aries*	Cres, Croatia	0	44.86	14.39	This study, PCR	B	mEU
44	CRS2-HRV	24552790***	*Ovis aries*	Cres, Croatia	0	44.86	14.39	This study, PCR	B	mEU
45	KORS1-HRV	24552790***	*Ovis aries*	Kornati, Croatia	0	43.81	15.31	This study, PCR	B	mEU
46	KORS2-HRV	24552790***	*Ovis aries*	Kornati, Croatia	0	43.81	15.31	This study, PCR	B	mEU
47	KRKS1-HRV	24552790***	*Ovis aries*	Krk, Croatia	0	45.08	14.59	This study, PCR	B	mEU
48	KRKS2-HRV	24552790***	*Ovis aries*	Krk, Croatia	0	45.08	14.59	This study, PCR	B	mEU
49	KRKS3-HRV	24552790***	*Ovis aries*	Krk, Croatia	0	45.08	14.59	This study, PCR	B	mEU
50	PDAL1-HRV	24552790***	*Ovis aries*	Dalmatia, Croatia	0	44.11	15.23	This study, PCR	B	mEU
51	PDAL2-HRV	24552790***	*Ovis aries*	Dalmatia, Croatia	0	44.11	15.23	This study, PCR	B	mEU
52	BNT1-DEU	24552790***	*Ovis aries*	Bentheim, Germany	0	52.50	7.05	This study, PCR	B	mEU
53	BNT2-DEU	24552790***	*Ovis aries*	Bentheim, Germany	0	52.50	7.05	This study, PCR	B	mEU
54	BNT3-DEU	24552790***	*Ovis aries*	Bentheim, Germany	0	52.50	7.05	This study, PCR	B	mEU
55	BNT4-DEU	24552790***	*Ovis aries*	Bentheim, Germany	0	52.50	7.05	This study, PCR	B	mEU
56	CBR1-DEU	24552790***	*Ovis aries*	Coburg, Germany	0	50.27	10.96	This study, PCR	B	mEU
57	CBR2-DEU	24552790***	*Ovis aries*	Coburg, Germany	0	50.27	10.96	This study, PCR	A	mEU
58	CBR3-DEU	24552790***	*Ovis aries*	Coburg, Germany	0	50.27	10.96	This study, PCR	B	mEU
59	CBR4-DEU	24552790***	*Ovis aries*	Coburg, Germany	0	50.27	10.96	This study, PCR	B	mEU
60	EFW1-DEU	24552790***	*Ovis aries*	East Frisia, Germany	0	53.23	7.47	This study, PCR	B	mEU
61	EFW2-DEU	24552790***	*Ovis aries*	East Frisia, Germany	0	53.23	7.47	This study, PCR	A	mEU
62	EFW3-DEU	24552790***	*Ovis aries*	East Frisia, Germany	0	53.23	7.47	This study, PCR	A	mEU
63	EFW4-DEU	24552790***	*Ovis aries*	East Frisia, Germany	0	53.23	7.47	This study, PCR	A	mEU
64	GBHM1-DEU	24552790***	*Ovis aries*	Northrhine-Westphalia, Germany	0	51.54	7.48	This study, PCR	B	mEU
65	GBHM2-DEU	24552790***	*Ovis aries*	Northrhine-Westphalia, Germany	0	51.54	7.48	This study, PCR	B	mEU
66	GBHM3-DEU	24552790***	*Ovis aries*	Northrhine-Westphalia, Germany	0	51.54	7.48	This study, PCR	B	mEU
67	GBHM4-DEU	24552790***	*Ovis aries*	Northrhine-Westphalia, Germany	0	51.54	7.48	This study, PCR	B	mEU
68	GBRM1-DEU	24552790***	*Ovis aries*	Bavaria, Germany	0	49.04	11.33	This study, PCR	B	mEU
69	GBRM2-DEU	24552790***	*Ovis aries*	Bavaria, Germany	0	49.04	11.33	This study, PCR	B	mEU
70	GBRM3-DEU	24552790***	*Ovis aries*	Bavaria, Germany	0	49.04	11.33	This study, PCR	B	mEU
71	GBRM4-DEU	24552790***	*Ovis aries*	Bavaria, Germany	0	49.04	11.33	This study, PCR	B	mEU
72	GHS1-DEU	24552790***	*Ovis aries*	Lüneburger Heide, Germany	0	53.17	9.92	This study, PCR	A	mEU
73	GHS2-DEU	24552790***	*Ovis aries*	Lüneburger Heide, Germany	0	53.17	9.92	This study, PCR	B	mEU
74	GHS3-DEU	24552790***	*Ovis aries*	Lüneburger Heide, Germany	0	53.17	9.92	This study, PCR	B	mEU
75	GHS4-DEU	24552790***	*Ovis aries*	Lüneburger Heide, Germany	0	53.17	9.92	This study, PCR	B	mEU
76	MLS1-DEU	AF010406.1	*Ovis aries*	South/Central Germany	0	49.04	11.33	(33)	B	mEU
77	MLS2-DEU	24552790***	*Ovis aries*	South/Central Germany	0	49.04	11.33	This study, PCR	B	mEU
78	MLS3-DEU	24552790***	*Ovis aries*	South/Central Germany	0	49.04	11.33	This study, PCR	B	mEU
79	MLS4-DEU	24552790***	*Ovis aries*	South/Central Germany	0	49.04	11.33	This study, PCR	B	mEU
80	MLS5-DEU	24552790***	*Ovis aries*	South/Central Germany	0	49.04	11.33	This study, PCR	B	mEU
81	MLS6-DEU	NC_001941.1	*Ovis aries*	South/Central Germany	0	49.04	11.33	(33)	B	mEU
82	PMC1-DEU	24552790***	*Ovis aries*	Baltic sea shores, Germany	0	54.40	13.36	This study, PCR	B	mEU
83	PMC2-DEU	24552790***	*Ovis aries*	Baltic sea shores, Germany	0	54.40	13.36	This study, PCR	A	mEU
84	PMC3-DEU	24552790***	*Ovis aries*	Baltic sea shores, Germany	0	54.40	13.36	This study, PCR	B	mEU
85	RHN1-DEU	24552790***	*Ovis aries*	Rhoen, Germany	0	50.47	10.07	This study, PCR	B	mEU
86	RHN2-DEU	24552790***	*Ovis aries*	Rhoen, Germany	0	50.47	10.07	This study, PCR	B	mEU
87	RHN3-DEU	24552790***	*Ovis aries*	Rhoen, Germany	0	50.47	10.07	This study, PCR	B	mEU
88	RHN4-DEU	24552790***	*Ovis aries*	Rhoen, Germany	0	50.47	10.07	This study, PCR	B	mEU
89	SRP1-GBR	24552790***	*Ovis aries*	Shropshire, UK	0	52.67	-2.80	This study, PCR	B	mEU
90	SRP2-GBR	24552790***	*Ovis aries*	Shropshire, UK	0	52.67	-2.80	This study, PCR	B	mEU
91	SRP3-GBR	24552790***	*Ovis aries*	Shropshire, UK	0	52.67	-2.80	This study, PCR	B	mEU
92	SRP4-GBR	24552790***	*Ovis aries*	Shropshire, UK	0	52.67	-2.80	This study, PCR	B	mEU
93	SUF01-GBR	24552790***	*Ovis aries*	Suffolk, UK	0	52.23	1.02	This study, PCR	B	mEU
94	SUF02-GBR	24552790***	*Ovis aries*	Suffolk, UK	0	52.23	1.02	This study, PCR	B	mEU
95	SUF03-GBR	24552790***	*Ovis aries*	Suffolk, UK	0	52.23	1.02	This study, PCR	B	mEU
96	SUF04-GBR	24552790***	*Ovis aries*	Suffolk, UK	0	52.23	1.02	This study, PCR	B	mEU
97	MER1-AUS	HM236174.1	*Ovis aries*	Extremadura, Spain	0	39.21	-6.11	(31)	A	mEU
98	AAL1-FIN	KF938342.1	*Ovis aries*	Åland, Finland	0	60.08	19.93	(30)	A	mEU
99	FIN1-FIN	KF938354.1	*Ovis aries*	Helsinki, Finland	0	60.16	24.92	(30)	B	mEU
100	FIN2-FIN	KF938355.1	*Ovis aries*	Helsinki, Finland	0	60.16	24.92	(30)	B	mEU

101	FIN3-FIN	SRS335716	*Ovis aries*	Helsinki, Finland	0	60.16	24.92	SRA	B	mEU
102	FND1-FIN	EF490451.1	*Ovis aries*	Helsinki, Finland	0	60.16	24.92	(34)	B	mEU
103	FND2-FIN	EF490452.1	*Ovis aries*	Helsinki, Finland	0	60.16	24.92	(34)	B	mEU
104	FND3-FIN	EF490453.1	*Ovis aries*	Helsinki, Finland	0	60.16	24.92	(34)	B	mEU
105	FND4-FIN	EF490454.1	*Ovis aries*	Helsinki, Finland	0	60.16	24.92	(34)	B	mEU
106	FND5-FIN	EF490455.1	*Ovis aries*	Helsinki, Finland	0	60.16	24.92	(34)	B	mEU
107	KNG1-FIN	KF938353.1	*Ovis aries*	Kainuu, Finland	0	64.53	28.50	(30)	B	mEU
108	CHR1-FRA	24552790***	*Ovis aries*	Charolles, France	0	46.45	4.28	This study, PCR	B	mEU
109	CHR2-FRA	24552790***	*Ovis aries*	Charolles, France	0	46.45	4.28	This study, PCR	B	mEU
110	CHR3-FRA	24552790***	*Ovis aries*	Charolles, France	0	46.45	4.28	This study, PCR	B	mEU
111	IDF1-FRA	24552790***	*Ovis aries*	Île-de-France, France	0	48.73	2.45	This study, PCR	B	mEU
112	IDF2-FRA	24552790***	*Ovis aries*	Île-de-France, France	0	48.73	2.45	This study, PCR	B	mEU
113	IDF3-FRA	24552790***	*Ovis aries*	Île-de-France, France	0	48.73	2.45	This study, PCR	B	mEU
114	LAC1-FRA	KF302447.1	*Ovis aries*	Lacaune, France	0	43.71	2.69	(35)	B	mEU
115	LAC2-FRA	KF302453.1	*Ovis aries*	Lacaune, France	0	43.71	2.69	(35)	B	mEU
116	LAC3-FRA	KF302460.1	*Ovis aries*	Lacaune, France	0	43.71	2.69	(35)	B	mEU
117	RDR1-FRA	24552790***	*Ovis aries*	Pyrenees, France	0	42.66	2.26	This study, PCR	B	mEU
118	RDR2-FRA	24552790***	*Ovis aries*	Pyrenees, France	0	42.66	2.26	This study, PCR	B	mEU
119	RDR3-FRA	24552790***	*Ovis aries*	Pyrenees, France	0	42.66	2.26	This study, PCR	B	mEU
120	RDR4-FRA	24552790***	*Ovis aries*	Pyrenees, France	0	42.66	2.26	This study, PCR	B	mEU
121	RMB01-FRA	SRS1709705	*Ovis aries*	Rambouillet, France	0	48.64	1.83	SRA	B	mEU
122	RMB02-FRA	SRS1709706	*Ovis aries*	Rambouillet, France	0	48.64	1.83	SRA	B	mEU
123	RMB03-FRA	SRS1709707	*Ovis aries*	Rambouillet, France	0	48.64	1.83	SRA	B	mEU
124	RMB04-FRA	SRS1709709	*Ovis aries*	Rambouillet, France	0	48.64	1.83	SRA	B	mEU
125	RMB05-FRA	SRS1709711	*Ovis aries*	Rambouillet, France	0	48.64	1.83	SRA	B	mEU
126	RMB06-FRA	SRS1709510	*Ovis aries*	Rambouillet, France	0	48.64	1.83	SRA	B	mEU
127	RMB07-FRA	SRS1709716	*Ovis aries*	Rambouillet, France	0	48.64	1.83	SRA	B	mEU
128	RMB08-FRA	SRS1709714	*Ovis aries*	Rambouillet, France	0	48.64	1.83	SRA	B	mEU
129	RMB09-FRA	SRS1709712	*Ovis aries*	Rambouillet, France	0	48.64	1.83	SRA	B	mEU
130	RMB10-FRA	SRS1709717	*Ovis aries*	Rambouillet, France	0	48.64	1.83	SRA	B	mEU
131	CHV1-GBR	SRS335729	*Ovis aries*	Cheviot Hills, UK	0	55.48	-2.15	SRA	B	mEU
132	CHV2-GBR	SRS335690	*Ovis aries*	Cheviot Hills, UK	0	55.48	-2.15	SRA	B	mEU
133	DSH1-GBR	SRS1709526	*Ovis aries*	Dorsetshire, UK	0	50.71	-2.44	SRA	B	mEU
134	DSH2-GBR	SRS1709528	*Ovis aries*	Dorsetshire, UK	0	50.71	-2.44	SRA	B	mEU
135	DSH3-GBR	SRS1709527	*Ovis aries*	Dorsetshire, UK	0	50.71	-2.44	SRA	B	mEU
136	DSH4-GBR	SRS1709561	*Ovis aries*	Dorsetshire, UK	0	50.71	-2.44	SRA	B	mEU
137	DWM1-GBR	SRS335709	*Ovis aries*	Dollgellau, Wales	0	52.74	-3.88	SRA	B	mEU
138	OXD1-GBR	KF938359.1	*Ovis aries*	Oxford, UK	0	51.77	-1.28	(30)	B	mEU
139	RMY1-AUS	HM236175.1	*Ovis aries*	Romney Marsh, UK	0	50.99	0.89	(31)	A	mEU
140	RMY2-GBR	SRS335691	*Ovis aries*	Romney Marsh, UK	0	50.99	0.89	SRA	A	mEU
141	TRWM1-GBR	SRS335715	*Ovis aries*	Tregaron, Wales, UK	0	52.23	-3.94	SRA	B	mEU
142	WHSF1-GBR	SRS335714	*Ovis aries*	Central Wales, UK	0	52.38	-3.68	SRA	B	mEU
143	APP1-ITA	KF302450.1	*Ovis aries*	Apennine Mountains, Italy	0	43.61	12.24	(35)	B	mEU
144	APP2-ITA	KF302451.1	*Ovis aries*	Apennine Mountains, Italy	0	43.61	12.24	(35)	B	mEU
145	COM1-ITA	KF302440.1	*Ovis aries*	Comiso, Province of Ragusa, Sicily, Italy	0	36.96	14.61	(35)	A	mEU
146	COM2-ITA	KF302441.1	*Ovis aries*	Comiso, Province of Ragusa, Sicily, Italy	0	36.96	14.61	(35)	A	mEU
147	COM3-ITA	KF302442.1	*Ovis aries*	Comiso, Province of Ragusa, Sicily, Italy	0	36.96	14.61	(35)	A	mEU
148	COM4-ITA	KF302443.1	*Ovis aries*	Comiso, Province of Ragusa, Sicily, Italy	0	36.96	14.61	(35)	A	mEU
149	COM5-ITA	KF302444.1	*Ovis aries*	Comiso, Province of Ragusa, Sicily, Italy	0	36.96	14.61	(35)	A	mEU
150	COM6-ITA	KF302445.1	*Ovis aries*	Comiso, Province of Ragusa, Sicily, Italy	0	36.96	14.61	(35)	A	mEU
151	COM7-ITA	KF302452.1	*Ovis aries*	Comiso, Province of Ragusa, Sicily, Italy	0	36.96	14.61	(35)	B	mEU

152	COM8-ITA	KF302461.1	*Ovis aries*	Comiso, Province of Ragusa, Sicily, Italy	0	36.96	14.61	(35)	B	mEU
153	COM9-ITA	KF302462.1	*Ovis aries*	Comiso, Province of Ragusa, Sicily, Italy	0	36.96	14.61	(35)	B	mEU
154	GEN1-ITA	KF302448.1	*Ovis aries*	Tavoliere delle Puglie, Italy	0	41.47	15.53	(35)	B	mEU
155	GEN2-ITA	KF302455.1	*Ovis aries*	Tavoliere delle Puglie, Italy	0	41.47	15.53	(35)	B	mEU
156	GEN3-ITA	KF302457.1	*Ovis aries*	Tavoliere delle Puglie, Italy	0	41.47	15.53	(35)	B	mEU
157	MNZ1-ITA	KF302446.1	*Ovis aries*	Abruzzo, Italy	0	42.25	13.79	(35)	A	mEU
158	MNZ2-ITA	KF302459.1	*Ovis aries*	Abruzzo, Italy	0	42.25	13.79	(35)	B	mEU
159	SOP1-ITA	KF302449.1	*Ovis aries*	Macerata, Italy	0	43.30	13.45	(35)	B	mEU
160	SOP2-ITA	KF302454.1	*Ovis aries*	Macerata, Italy	0	43.30	13.45	(35)	B	mEU
161	SOP3-ITA	KF302456.1	*Ovis aries*	Macerata, Italy	0	43.30	13.45	(35)	B	mEU
162	SOP4-ITA	KF302458.1	*Ovis aries*	Macerata, Italy	0	43.30	13.45	(35)	B	mEU
163	OARM1-DEU	HM236184.1	*Ovis aries musimon*	Sardinia/Corsica, Italy	0	41.22	9.21	(31)	B	mEU
164	OARM2-DEU	HM236185.1	*Ovis aries musimon*	Sardinia/Corsica, Italy	0	41.22	9.21	(31)	B	mEU
165	TXL1-NLD	KJ954145.1	*Ovis aries*	Texel island, Netherlands	0	53.08	4.81	GenBank	B	mEU
166	TXL2-NLD	24552790***	*Ovis aries*	Texel island, Netherlands	0	53.08	4.81	This study, PCR	B	mEU
167	TXL3-NLD	24552790***	*Ovis aries*	Texel island, Netherlands	0	53.08	4.81	This study, PCR	B	mEU
168	TXL4-NLD	24552790***	*Ovis aries*	Texel island, Netherlands	0	53.08	4.81	This study, PCR	B	mEU
169	TXL5-NLD	24552790***	*Ovis aries*	Texel island, Netherlands	0	53.08	4.81	This study, PCR	B	mEU
170	SWN1-POL	KF938349.1	*Ovis aries*	Łódź Voivodeship, Poland	0	51.73	19.47	(30)	B	mEU
171	WRZ1-POL	KF938350.1	*Ovis aries*	Białystok, Poland	0	53.13	23.15	(30)	B	mEU
172	TZYG1-RUS	24552790***	*Ovis aries*	Carpathian Mountains, Romania	0	47.33	25.55	This study, PCR	B	mEU
173	TZYG2-RUS	24552790***	*Ovis aries*	Carpathian Mountains, Romania	0	47.33	25.55	This study, PCR	B	mEU
174	KUCH1-RUS	24552790***	*Ovis aries*	Kuchugury, Oblast Voronezh, Russia	0	51.60	38.30	This study, PCR	B	mEU
175	KUCH2-RUS	24552790***	*Ovis aries*	Kuchugury, Oblast Voronezh, Russia	0	51.60	38.30	This study, PCR	A	mEU
176	KUIB1-RUS	KF938346.1	*Ovis aries*	Samara, Russia	0	53.23	50.22	(30)	B	mEU
177	KUIB2-RUS	24552790***	*Ovis aries*	Samara, Russia	0	53.23	50.22	This study, PCR	B	mEU
178	KUIB3-RUS	24552790***	*Ovis aries*	Samara, Russia	0	53.23	50.22	This study, PCR	B	mEU
179	OPR1-RUS	KF938352.1	*Ovis aries*	Oparino, Oblast Kirow, Russia	0	59.88	48.29	(30)	B	mEU
180	RMNV1-RUS	24552790***	*Ovis aries*	Upper Volga region, Russia	0	56.68	36.58	This study, PCR	A	mEU
181	RMNV2-RUS	24552790***	*Ovis aries*	Upper Volga region, Russia	0	56.68	36.58	This study, PCR	A	mEU
182	RMNV3-RUS	24552790***	*Ovis aries*	Upper Volga region, Russia	0	56.68	36.58	This study, PCR	B	mEU
183	RMNV4-RUS	24552790***	*Ovis aries*	Upper Volga region, Russia	0	56.68	36.58	This study, PCR	B	mEU
184	RMNV5-RUS	24552790***	*Ovis aries*	Upper Volga region, Russia	0	56.68	36.58	This study, PCR	B	mEU
185	RULH1-RUS	24552790***	*Ovis aries*	Voronezh Oblast, Russia	0	51.05	40.12	This study, PCR	B	mEU
186	RULH2-RUS	24552790***	*Ovis aries*	Voronezh Oblast, Russia	0	51.05	40.12	This study, PCR	B	mEU
187	RUV1-RUS	KF938356.1	*Ovis aries*	Viena Karelia, Russia	0	64.31	32.83	(30)	B	mEU
188	UDM1-RUS	KF938341.1	*Ovis aries*	Udmurtia, Russia	0	57.23	52.80	(30)	B	mEU
189	SBF1-GBR	24552790***	*Ovis aries*	Scottish/English border, UK	0	55.32	-2.71	This study, PCR	B	mEU
190	SBF2-GBR	24552790***	*Ovis aries*	Scottish/English border, UK	0	55.32	-2.71	This study, PCR	B	mEU
191	SBF3-GBR	24552790***	*Ovis aries*	Scottish/English border, UK	0	55.32	-2.71	This study, PCR	B	mEU
192	SBF4-GBR	24552790***	*Ovis aries*	Scottish/English border, UK	0	55.32	-2.71	This study, PCR	B	mEU
193	SOA1-GBR	24552790***	*Ovis aries*	Soay, Scotland, UK	0	57.15	-6.22	This study, PCR	B	mEU
194	SOA2-GBR	24552790***	*Ovis aries*	Soay, Scotland, UK	0	57.15	-6.22	This study, PCR	B	mEU
195	SOA3-GBR	24552790***	*Ovis aries*	Soay, Scotland, UK	0	57.15	-6.22	This study, PCR	B	mEU
196	KRST1-SVN	24552790***	*Ovis aries*	Bovec, Slovenia	0	46.34	13.55	This study, PCR	B	mEU
197	KRST2-SVN	24552790***	*Ovis aries*	Bovec, Slovenia	0	46.34	13.55	This study, PCR	B	mEU
198	KRST3-SVN	24552790***	*Ovis aries*	Bovec, Slovenia	0	46.34	13.55	This study, PCR	B	mEU
199	PSJE1-SRB	24552790***	*Ovis aries*	Sjenica, Serbia	0	43.28	20.00	This study, PCR	A	mEU
200	PSJE2-SRB	24552790***	*Ovis aries*	Sjenica, Serbia	0	43.28	20.00	This study, PCR	A	mEU
201	PSRB1-SRB	KF938347.1	*Ovis aries*	Belgrade, Serbia	0	44.74	20.44	(30)	B	mEU
202	CMF1-UKR	KF938357.1	*Ovis aries*	Zakarpattia, Ukraine	0	48.45	23.24	(30)	B	mEU
203	CCP1-TUR	SRS335708	*Ovis aries*	Aydın Province, Türkiye	0	37.84	27.84	SRA	B	mWAN
204	SKZ1-TUR	SRS335711	*Ovis aries*	Izmir, Türkiye	0	38.42	27.19	SRA	B	mWAN
205	SKZ2-TUR	SRS335675	*Ovis aries*	Izmir, Türkiye	0	38.42	27.19	SRA	B	mWAN
206	OUD1-MAR	ERS154859	*Ovis aries*	Ouled Djellal, Sidi Moussa, Algeria	0	34.6431	-2.1639	SRA	B	mAFR
207	OUD2-MAR	ERS154855	*Ovis aries*	Ouled Djellal, Jerrada, Algeria	0	33.8475	-2.6312	SRA	A	mAFR

208	OUD3-MAR	ERS154849	*Ovis aries*	Ouled Djellal, Algeria	0	34.45	5.11	SRA	B	mAFR
209	OUD4-MAR	ERS154816	*Ovis aries*	Ouled Djellal, Algeria	0	34.45	5.11	SRA	B	mAFR
210	OUD5-MAR	ERS154851	*Ovis aries*	Ouled Djellal, Algeria	0	34.45	5.11	SRA	B	mAFR
211	OUD6-MAR	ERS154703	*Ovis aries*	Ouled Djellal, Algeria	0	34.45	5.11	SRA	B	mAFR
212	OUD7-MAR	ERS154861	*Ovis aries*	Ouled Djellal, Algeria	0	34.45	5.11	SRA	B	mAFR
213	OUD8-MAR	ERS154856	*Ovis aries*	Ouled Djellal, Algeria	0	34.45	5.11	SRA	B	mAFR
214	BGL1-MAR	ERS154854	*Ovis aries*	Beni Guil tribal area, Morocco	0	32.83	-2.08	SRA	B	mAFR
215	BGL2-MAR	ERS154840	*Ovis aries*	Beni Guil tribal area, Morocco	0	32.83	-2.08	SRA	B	mAFR
216	BGL3-MAR	ERS154844	*Ovis aries*	Beni Guil tribal area, Morocco	0	32.83	-2.08	SRA	C	mAFR
217	BGL4-MAR	ERS154700	*Ovis aries*	Beni Guil tribal area, Morocco	0	32.83	-2.08	SRA	B	mAFR
218	BGL5-MAR	ERS154838	*Ovis aries*	Beni Guil tribal area, Morocco	0	32.83	-2.08	SRA	B	mAFR
219	BGL6-MAR	ERS154706	*Ovis aries*	Beni Guil tribal area, Morocco	0	32.83	-2.08	SRA	B	mAFR
220	BJD1-MAR	ERS154762	*Ovis aries*	Middle Atlas, Morocco	0	33.50	-4.50	SRA	B	mAFR
221	DMN01-MAR	ERS154835	*Ovis aries*	Tafilalt, Morocco	0	31.30	-4.30	SRA	B	mAFR
222	DMN02-MAR	ERS154824	*Ovis aries*	Tafilalt, Morocco	0	31.30	-4.30	SRA	B	mAFR
223	DMN03-MAR	ERS154822	*Ovis aries*	Tafilalt, Morocco	0	31.30	-4.30	SRA	B	mAFR
224	DMN04-MAR	ERS154813	*Ovis aries*	Tafilalt, Morocco	0	31.30	-4.30	SRA	B	mAFR
225	DMN05-MAR	ERS154784	*Ovis aries*	Tafilalt, Morocco	0	31.30	-4.30	SRA	B	mAFR
226	DMN06-MAR	ERS154786	*Ovis aries*	Tafilalt, Morocco	0	31.30	-4.30	SRA	B	mAFR
227	DMN07-MAR	ERS154799	*Ovis aries*	Tafilalt, Morocco	0	31.30	-4.30	SRA	B	mAFR
228	DMN08-MAR	ERS154798	*Ovis aries*	Tafilalt, Morocco	0	31.30	-4.30	SRA	B	mAFR
229	DMN09-MAR	ERS154812	*Ovis aries*	Tafilalt, Morocco	0	31.30	-4.30	SRA	B	mAFR
230	DMN10-MAR	ERS154785	*Ovis aries*	Tafilalt, Morocco	0	31.30	-4.30	SRA	B	mAFR
231	DMN11-MAR	ERS154767	*Ovis aries*	Tafilalt, Morocco	0	31.30	-4.30	SRA	B	mAFR
232	DMN12-MAR	ERS154774	*Ovis aries*	Tafilalt, Morocco	0	31.30	-4.30	SRA	B	mAFR
233	DMN13-MAR	ERS154787	*Ovis aries*	Tafilalt, Morocco	0	31.30	-4.30	SRA	B	mAFR
234	DMN14-MAR	ERS154783	*Ovis aries*	Tafilalt, Morocco	0	31.30	-4.30	SRA	B	mAFR
235	DMN15-MAR	ERS154768	*Ovis aries*	Tafilalt, Morocco	0	31.30	-4.30	SRA	B	mAFR
236	DMN16-MAR	ERS154744	*Ovis aries*	Tafilalt, Morocco	0	31.30	-4.30	SRA	A	mAFR
237	DMN17-MAR	ERS154735	*Ovis aries*	Tafilalt, Morocco	0	31.30	-4.30	SRA	B	mAFR
238	DMN18-MAR	ERS154765	*Ovis aries*	Tafilalt, Morocco	0	31.30	-4.30	SRA	B	mAFR
239	DMN19-MAR	ERS154766	*Ovis aries*	Tafilalt, Morocco	0	31.30	-4.30	SRA	B	mAFR
240	DMN20-MAR	ERS154758	*Ovis aries*	Tafilalt, Morocco	0	31.30	-4.30	SRA	B	mAFR
241	DMN21-MAR	ERS154756	*Ovis aries*	Tafilalt, Morocco	0	31.30	-4.30	SRA	B	mAFR
242	DMN22-MAR	ERS154743	*Ovis aries*	Tafilalt, Morocco	0	31.30	-4.30	SRA	B	mAFR
243	DMN23-MAR	ERS154733	*Ovis aries*	Tafilalt, Morocco	0	31.30	-4.30	SRA	B	mAFR
244	DMN24-MAR	ERS154725	*Ovis aries*	Tafilalt, Morocco	0	31.30	-4.30	SRA	B	mAFR
245	DMN25-MAR	ERS154717	*Ovis aries*	Tafilalt, Morocco	0	31.30	-4.30	SRA	B	mAFR
246	DMN26-MAR	ERS154711	*Ovis aries*	Tafilalt, Morocco	0	31.30	-4.30	SRA	B	mAFR
247	DMN27-MAR	ERS154814	*Ovis aries*	Tafilalt, Morocco	0	31.30	-4.30	SRA	B	mAFR
248	DMN28-MAR	ERS154800	*Ovis aries*	Tafilalt, Morocco	0	31.30	-4.30	SRA	B	mAFR
249	DMN29-MAR	ERS154775	*Ovis aries*	Tafilalt, Morocco	0	31.30	-4.30	SRA	B	mAFR
250	DMN30-MAR	ERS154734	*Ovis aries*	Tafilalt, Morocco	0	31.30	-4.30	SRA	B	mAFR
251	SAR01-MAR	ERS154843	*Ovis aries*	Central Morocco, Morocco	0	32.80	-7.40	SRA	B	mAFR
252	SAR02-MAR	ERS154823	*Ovis aries*	Central Morocco, Morocco	0	32.80	-7.40	SRA	B	mAFR
253	SAR03-MAR	ERS154771	*Ovis aries*	Central Morocco, Morocco	0	32.80	-7.40	SRA	B	mAFR
254	SAR04-MAR	ERS154763	*Ovis aries*	Central Morocco, Morocco	0	32.80	-7.40	SRA	B	mAFR
255	SAR05-MAR	ERS154752	*Ovis aries*	Central Morocco, Morocco	0	32.80	-7.40	SRA	B	mAFR
256	SAR06-MAR	ERS154761	*Ovis aries*	Central Morocco, Morocco	0	32.80	-7.40	SRA	B	mAFR
257	SAR07-MAR	ERS154760	*Ovis aries*	Central Morocco, Morocco	0	32.80	-7.40	SRA	B	mAFR
258	SAR08-MAR	ERS154759	*Ovis aries*	Central Morocco, Morocco	0	32.80	-7.40	SRA	B	mAFR
259	SAR09-MAR	ERS154754	*Ovis aries*	Central Morocco, Morocco	0	32.80	-7.40	SRA	B	mAFR
260	SAR10-MAR	ERS154753	*Ovis aries*	Central Morocco, Morocco	0	32.80	-7.40	SRA	B	mAFR
261	SAR11-MAR	ERS154747	*Ovis aries*	Central Morocco, Morocco	0	32.80	-7.40	SRA	B	mAFR
262	SAR12-MAR	ERS154738	*Ovis aries*	Central Morocco, Morocco	0	32.80	-7.40	SRA	B	mAFR
263	SAR13-MAR	ERS154745	*Ovis aries*	Central Morocco, Morocco	0	32.80	-7.40	SRA	B	mAFR
264	SAR14-MAR	ERS154740	*Ovis aries*	Central Morocco, Morocco	0	32.80	-7.40	SRA	B	mAFR
265	SAR15-MAR	ERS154737	*Ovis aries*	Central Morocco, Morocco	0	32.80	-7.40	SRA	B	mAFR

266	SAR16-MAR	ERS154731	*Ovis aries*	Central Morocco, Morocco	0	32.80	-7.40	SRA	B	mAFR
267	SAR17-MAR	ERS154730	*Ovis aries*	Central Morocco, Morocco	0	32.80	-7.40	SRA	B	mAFR
268	SAR18-MAR	ERS154728	*Ovis aries*	Central Morocco, Morocco	0	32.80	-7.40	SRA	B	mAFR
269	SAR19-MAR	ERS154722	*Ovis aries*	Central Morocco, Morocco	0	32.80	-7.40	SRA	B	mAFR
270	SAR20-MAR	ERS154720	*Ovis aries*	Central Morocco, Morocco	0	32.80	-7.40	SRA	B	mAFR
271	SAR21-MAR	ERS154716	*Ovis aries*	Central Morocco, Morocco	0	32.80	-7.40	SRA	C	mAFR
272	SAR22-MAR	ERS154701	*Ovis aries*	Central Morocco, Morocco	0	32.80	-7.40	SRA	B	mAFR
273	SAR23-MAR	ERS154764	*Ovis aries*	Central Morocco, Morocco	0	32.80	-7.40	SRA	B	mAFR
274	SAR24-MAR	ERS154721	*Ovis aries*	Central Morocco, Morocco	0	32.80	-7.40	SRA	B	mAFR
275	SAR25-MAR	ERS154727	*Ovis aries*	Central Morocco, Morocco	0	32.80	-7.40	SRA	B	mAFR
276	SAR26-MAR	ERS154746	*Ovis aries*	Central Morocco, Morocco	0	32.80	-7.40	SRA	B	mAFR
277	SAR27-MAR	ERS154729	*Ovis aries*	Central Morocco, Morocco	0	32.80	-7.40	SRA	B	mAFR
278	TMD01-MAR	ERS154830	*Ovis aries*	Timahdite, Morocco	0	33.24	-5.06	SRA	B	mAFR
279	TMD02-MAR	ERS154829	*Ovis aries*	Timahdite, Morocco	0	33.24	-5.06	SRA	B	mAFR
280	TMD03-MAR	ERS154820	*Ovis aries*	Timahdite, Morocco	0	33.24	-5.06	SRA	B	mAFR
281	TMD04-MAR	ERS154819	*Ovis aries*	Timahdite, Morocco	0	33.24	-5.06	SRA	B	mAFR
282	TMD05-MAR	ERS154808	*Ovis aries*	Timahdite, Morocco	0	33.24	-5.06	SRA	B	mAFR
283	TMD06-MAR	ERS154796	*Ovis aries*	Timahdite, Morocco	0	33.24	-5.06	SRA	B	mAFR
284	TMD07-MAR	ERS154806	*Ovis aries*	Timahdite, Morocco	0	33.24	-5.06	SRA	B	mAFR
285	TMD08-MAR	ERS154794	*Ovis aries*	Timahdite, Morocco	0	33.24	-5.06	SRA	B	mAFR
286	TMD09-MAR	ERS154782	*Ovis aries*	Timahdite, Morocco	0	33.24	-5.06	SRA	B	mAFR
287	TMD10-MAR	ERS154818	*Ovis aries*	Timahdite, Morocco	0	33.24	-5.06	SRA	B	mAFR
288	TMD11-MAR	ERS154797	*Ovis aries*	Timahdite, Morocco	0	33.24	-5.06	SRA	B	mAFR
289	TMD12-MAR	ERS154780	*Ovis aries*	Timahdite, Morocco	0	33.24	-5.06	SRA	B	mAFR
290	TMD13-MAR	ERS154809	*Ovis aries*	Timahdite, Morocco	0	33.24	-5.06	SRA	B	mAFR
291	TMD14-MAR	ERS154770	*Ovis aries*	Timahdite, Morocco	0	33.24	-5.06	SRA	B	mAFR
292	TMD15-MAR	ERS154781	*Ovis aries*	Timahdite, Morocco	0	33.24	-5.06	SRA	B	mAFR
293	TMD16-MAR	ERS154807	*Ovis aries*	Timahdite, Morocco	0	33.24	-5.06	SRA	B	mAFR
294	EMZ1-ETH	SRS335697	*Ovis aries*	Amhara, Ethiopia	0	11.85	38.03	SRA	B	mAFR
295	DJL1-BEN	KF977845.1	*Ovis aries*	West/Central Africa, Benin	0	8.26	2.26	(32)	B	mAFR
296	NQA1-ZAF	SRS335740	*Ovis aries*	Namaqua, South Africa	0	-30.02	17.58	SRA	B	mAFR
297	RDA1-ZAF	SRS335722	*Ovis aries*	Upington, South Africa	0	-28.40	21.24	SRA	A	mAFR
298	RDA2-ZAF	SRS335702	*Ovis aries*	Upington, South Africa	0	-28.40	21.24	SRA	B	mAFR
299	SHL1-ZAF	KF977846.1	*Ovis aries*	Sahel, Central Africa	0	6.81	20.42	(32)	B	mAFR
300	BGE1-BGD	SRS335705	*Ovis aries*	Dhaka, Bangladesh	0	23.80	90.41	SRA	A	mEASIA
301	BGE2-BGD	SRS335686	*Ovis aries*	Dhaka, Bangladesh	0	23.80	90.41	SRA	A	mEASIA
302	ALT1-CHN	KF938320.1	*Ovis aries*	Altay, China	0	47.86	88.14	(30)	C	mEASIA
303	AWG1-CHN	KP998470.1	*Ovis aries*	Gonjo, Qamdo, Tibet, China	0	30.86	98.29	GenBank	A	mEASIA
304	BRK1-CHN	KF938321.1	*Ovis aries*	Bachu County, Xinjiang, China	0	39.75	78.55	(30)	A	mEASIA
305	BSB1-CHN	KF938330.1	*Ovis aries*	Yumin County, Xinjiang, China	0	46.20	82.97	(30)	A	mEASIA
306	BYB1-CHN	KF938331.1	*Ovis aries*	Hejing County, Xinjiang, China	0	42.88	85.25	(30)	A	mEASIA
307	DLG1-CHN	KF938332.1	*Ovis aries*	Shache County, Xingjiang, China	0	38.37	77.23	(30)	A	mEASIA
308	DLG2-CHN	SRS1071053	*Ovis aries*	Shache County, Xingjiang, China	0	38.37	77.23	SRA	B	mEASIA
309	GARZ01-CHN	KU681175	*Ovis aries*	Garze Tibetan aut. pref., China	0	31.64	99.98	(38)	B	mEASIA
310	GARZ02-CHN	KU681176	*Ovis aries*	Garze Tibetan aut. pref., China	0	31.64	99.98	(38)	B	mEASIA
311	GARZ03-CHN	KU681177	*Ovis aries*	Garze Tibetan aut. pref., China	0	31.64	99.98	(38)	A	mEASIA
312	GARZ04-CHN	KU681178	*Ovis aries*	Garze Tibetan aut. pref., China	0	31.64	99.98	(38)	A	mEASIA
313	GARZ05-CHN	KU681179	*Ovis aries*	Garze Tibetan aut. pref., China	0	31.64	99.98	(38)	A	mEASIA
314	GARZ06-CHN	KU681180	*Ovis aries*	Garze Tibetan aut. pref., China	0	31.64	99.98	(38)	B	mEASIA
315	GARZ07-CHN	KU681181	*Ovis aries*	Garze Tibetan aut. pref., China	0	31.64	99.98	(38)	A	mEASIA
316	GARZ08-CHN	KU681182	*Ovis aries*	Garze Tibetan aut. pref., China	0	31.64	99.98	(38)	B	mEASIA
317	GARZ09-CHN	KU681183	*Ovis aries*	Garze Tibetan aut. pref., China	0	31.64	99.98	(38)	B	mEASIA
318	GARZ10-CHN	KU681184	*Ovis aries*	Garze Tibetan aut. pref., China	0	31.64	99.98	(38)	A	mEASIA
319	GARZ11-CHN	KU681185	*Ovis aries*	Garze Tibetan aut. pref., China	0	31.64	99.98	(38)	B	mEASIA
320	GARZ12-CHN	KU681186	*Ovis aries*	Garze Tibetan aut. pref., China	0	31.64	99.98	(38)	B	mEASIA
321	GARZ13-CHN	KU681187	*Ovis aries*	Garze Tibetan aut. pref., China	0	31.64	99.98	(38)	C	mEASIA
322	GARZ14-CHN	KU681188	*Ovis aries*	Garze Tibetan aut. pref., China	0	31.64	99.98	(38)	C	mEASIA
323	GARZ15-CHN	KU681189	*Ovis aries*	Garze Tibetan aut. pref., China	0	31.64	99.98	(38)	C	mEASIA

324	GARZ16-CHN	KU681190	*Ovis aries*	Garze Tibetan aut. pref., China	0	31.64	99.98	(38)	B	mEASIA
325	GARZ17-CHN	KU681191	*Ovis aries*	Garze Tibetan aut. pref., China	0	31.64	99.98	(38)	C	mEASIA
326	GARZ18-CHN	KU681192	*Ovis aries*	Garze Tibetan aut. pref., China	0	31.64	99.98	(38)	B	mEASIA
327	GNJ1-CHN	KP998472.1	*Ovis aries*	Xiahe, Gannan, Gansu, China	0	35.05	102.52	GenBank	A	mEASIA
328	GSTB1-TIB	KP981379.1	*Ovis aries*	Gansu, China	0	37.33	103.33	GenBank	B	mEASIA
329	HLB1-CHN	KF938327.1	*Ovis aries*	Hulun Buir, Inner Mongolia, China	0	49.22	119.77	(30)	C	mEASIA
330	HTN1-CHN	KF938322.1	*Ovis aries*	Hetian, Xinjiang, China	0	37.22	79.92	(30)	A	mEASIA
331	HUB1-CHN	KP998471.1	*Ovis aries*	Zhongba County, China	0	30.58	83.77	GenBank	C	mEASIA
332	HZG1-CHN	KF938329.1	*Ovis aries*	Hanzhong, Shaanxi, China	0	33.08	107.03	(30)	B	mEASIA
333	JZG1-CHN	KF938328.1	*Ovis aries*	Jinzhong, Shanxi, China	0	37.69	112.75	(30)	B	mEASIA
334	KGZ1-KGZ	KF938334.1	*Ovis aries*	Kizilsu Kirghiz (aut. pref.), Xingjiang, China	0	39.97	75.06	(30)	A	mEASIA
335	KZK1-KAZ	KF938333.1	*Ovis aries*	Tekes County, Xinjiang, China	0	42.93	82.02	(30)	B	mEASIA
336	LPN1-CHN	KF938323.1	*Ovis aries*	Lop Nor, Qakilik, Bayingolin, China	0	40.50	90.50	(30)	A	mEASIA
337	LZLT1-CHN	KF938335.1	*Ovis aries*	Lanzhou, Gansu, China	0	36.04	103.86	(30)	B	mEASIA
338	MBF1-CHN	KF938318.1	*Ovis aries*	Minxian, Dingxi, Gansu, China	0	34.46	104.21	(30)	C	mEASIA
339	NGW01-CHN	KU681193	*Ovis aries*	Ngawa, Tibetan Qiang (aut. pref.), China	0	31.90	102.22	(38)	A	mEASIA
340	NGW02-CHN	KU681194	*Ovis aries*	Ngawa, Tibetan Qiang (aut. pref.), China	0	31.90	102.22	(38)	A	mEASIA
341	NGW03-CHN	KU681195	*Ovis aries*	Ngawa, Tibetan Qiang (aut. pref.), China	0	31.90	102.22	(38)	B	mEASIA
342	NGW04-CHN	KU681196	*Ovis aries*	Ngawa, Tibetan Qiang (aut. pref.), China	0	31.90	102.22	(38)	B	mEASIA
343	NGW05-CHN	KU681197	*Ovis aries*	Ngawa, Tibetan Qiang (aut. pref.), China	0	31.90	102.22	(38)	A	mEASIA
344	NGW06-CHN	KU681198	*Ovis aries*	Ngawa, Tibetan Qiang (aut. pref.), China	0	31.90	102.22	(38)	A	mEASIA
345	NGW07-CHN	KU681199	*Ovis aries*	Ngawa, Tibetan Qiang (aut. pref.), China	0	31.90	102.22	(38)	B	mEASIA
346	NGW08-CHN	KU681200	*Ovis aries*	Ngawa, Tibetan Qiang (aut. pref.), China	0	31.90	102.22	(38)	B	mEASIA
347	NGW09-CHN	KU681201	*Ovis aries*	Ngawa, Tibetan Qiang (aut. pref.), China	0	31.90	102.22	(38)	C	mEASIA
348	NGW10-CHN	KU681202	*Ovis aries*	Ngawa, Tibetan Qiang (aut. pref.), China	0	31.90	102.22	(38)	B	mEASIA
349	OULA1-CHN	KU575248	*Ovis aries*	Tibet, China	0	31.47	88.39	GenBank	A	mEASIA
350	OULA2-CHN	KT148968	*Ovis aries*	Tibet (altitude="3800 m"), China	0	31.47	88.39	GenBank	C	mEASIA
351	QHT1-CHN	KF938325.1	*Ovis aries*	Qinghai-tibetan Plateau, Qinghai, China	0	36.63	101.78	(30)	A	mEASIA
352	QLW1-CHN	KP998473.1	*Ovis aries*	Qilian, Haibei, Qinghai, China	0	38.20	99.98	GenBank	A	mEASIA
353	QRB1-CHN	KF938326.1	*Ovis aries*	Qira, Hotan, Xinjiang, China	0	37.35	81.00	(30)	A	mEASIA
354	STHL1-CHN	KP702285.1	*Ovis aries*	Hulun Buir, Inner Mongolia, China	0	49.22	119.77	GenBank	B	mEASIA
355	SUN1-CHN	KF938317.1	*Ovis aries*	Sonid Zuoqi, Inner Mongolia, China	0	44.03	113.30	(30)	A	mEASIA
356	TAN1-CHN	KF938336.1	*Ovis aries*	Yanchi County, Ningxia, China	0	37.69	107.07	(30)	A	mEASIA
357	TFB1-CHN	KF938324.1	*Ovis aries*	Turpan, Xinjiang, China	0	42.57	89.22	(30)	A	mEASIA
358	TKR1-CHN	KF938337.1	*Ovis aries*	Taxkorgan County, Xinjiang, China	0	37.79	75.23	(30)	A	mEASIA
359	UJQ1-CHN	KF938319.1	*Ovis aries*	East Ujimqin, Xilin Gol, China	0	45.82	117.62	(30)	A	mEASIA
360	UJQ2-CHN	KR868678.1	*Ovis aries*	East Ujimqin, Xilin Gol, China	0	45.82	117.62	(40)	B	mEASIA
361	YCH1-CHN	KF938338.1	*Ovis aries*	Yecheng County, Xinjiang, China	0	37.05	77.01	(30)	A	mEASIA
362	GUR1-IDN	SRS335718	*Ovis aries*	Garut, Jawa Barat, Indonesia	0	-7.30	107.85	SRA	B	mEASIA
363	GUR2-IDN	SRS335698	*Ovis aries*	Garut, Jawa Barat, Indonesia	0	-7.30	107.85	SRA	B	mEASIA
364	SUM51-IDN	SRS335713	*Ovis aries*	Sumatra, Indonesia	0	-0.66	100.88	SRA	A	mEASIA
365	SUM52-IDN	SRR501861	*Ovis aries*	Sumatra, Indonesia	0	-0.66	100.88	SRA	B	mEASIA
366	CHA1-IND	SRS335727	*Ovis aries*	Changtang, Ladakh, India	0	34.42	77.56	SRA	C	mEASIA
367	CHA2-IND	SRS335689	*Ovis aries*	Changtang, Ladakh, India	0	34.42	77.56	SRA	A	mEASIA
368	GAR1-IND	SRS335735	*Ovis aries*	Sundarbans, West Bengal, India	0	22.03	88.83	SRA	A	mEASIA
369	GAR2-IND	SRS335736	*Ovis aries*	Sundarbans, West Bengal, India	0	22.03	88.83	SRA	A	mEASIA
370	STHN1-MNG	KF977847.1	*Ovis aries*	Shandong, China	0	36.40	118.19	(32)	A	mEASIA
371	TIB1-TIB	SRS335677	*Ovis aries*	Tibetan Plateau, Chengxi, Xining, Qinghai, China	0	36.63	101.73	SRA	A	mEASIA
372	TIB2-TIB	SRS335695	*Ovis aries*	Tibetan Plateau, Chengxi, Xining, Qinghai, China	0	36.63	101.73	SRA	A	mEASIA
373	EDLB1-RUS	24552790***	*Ovis aries*	Astana, Kazakhstan	0	51.16	71.43	This study, PCR	A	mEASIA
374	EDLB2-RUS	24552790***	*Ovis aries*	Astana, Kazakhstan	0	51.16	71.43	This study, PCR	B	mEASIA
375	ALTM1-RUS	24552790***	*Ovis aries*	Altai Mountains, Mongolia	0	49.01	89.00	This study, PCR	A	mEASIA
376	ALTM2-RUS	24552790***	*Ovis aries*	Altai Mountains, Mongolia	0	49.01	89.00	This study, PCR	B	mEASIA
377	BDK1-RUS	KF938339.1	*Ovis aries*	Buryatia, Russia	0	50.85	105.44	(30)	B	mEASIA
378	BKFF1-RUS	24552790***	*Ovis aries*	Baïkal, Oblast Irkutsk, Russia	0	51.88	104.79	This study, PCR	B	mEASIA
379	BKFF2-RUS	24552790***	*Ovis aries*	Baïkal, Oblast Irkutsk, Russia	0	51.88	104.79	This study, PCR	A	mEASIA

380	BUUB1-RUS	24552790***	*Ovis aries*	Yakutia, Russia	0	66.85	130.25	This study, PCR	A	mEASIA
381	BUUB2-RUS	24552790***	*Ovis aries*	Yakutia, Russia	0	66.85	130.25	This study, PCR	A	mEASIA
382	KLND1-RUS	KF938358.1	*Ovis aries*	Kulunda, Altai Krai, Russia	0	52.56	78.93	(30)	B	mEASIA
383	KLND2-RUS	24552790***	*Ovis aries*	Kulunda, Altai Krai, Russia	0	52.56	78.93	This study, PCR	B	mEASIA
384	KLND3-RUS	24552790***	*Ovis aries*	Kulunda, Altai Krai, Russia	0	52.56	78.93	This study, PCR	B	mEASIA
385	TUVA1-RUS	24552790***	*Ovis aries*	Tuva, Russia	0	51.69	95.28	This study, PCR	A	mEASIA
386	TUVA2-RUS	24552790***	*Ovis aries*	Tuva, Russia	0	51.69	95.28	This study, PCR	A	mEASIA
387	TUSH1-RUS	KF938343.1	*Ovis aries*	Sagarejo district, Georgia	0	41.73	45.34	(30)	B	mCC
388	TUSH2-RUS	24552790***	*Ovis aries*	Sagarejo district, Georgia	0	41.73	45.34	This study, PCR	A	mCC
389	TUSH3-RUS	24552790***	*Ovis aries*	Sagarejo district, Georgia	0	41.73	45.34	This study, PCR	B	mCC
390	ANDB1-RUS	KF938340.1	*Ovis aries*	Andi, Dagestan, Russia	0	42.79	46.26	(30)	B	mCC
391	ANDB2-RUS	24552790***	*Ovis aries*	Andi, Dagestan, Russia	0	42.79	46.26	This study, PCR	B	mCC
392	ANDB3-RUS	24552790***	*Ovis aries*	Andi, Dagestan, Russia	0	42.79	46.26	This study, PCR	A	mCC
393	DAGM1-RUS	24552790***	*Ovis aries*	Dagestan, Russia	0	42.92	47	This study, PCR	B	mCC
394	DAGM2-RUS	24552790***	*Ovis aries*	Dagestan, Russia	0	42.92	47	This study, PCR	B	mCC
395	GRZN1-RUS	24552790***	*Ovis aries*	Grosny, Russia	0	43.32	45.7	This study, PCR	B	mCC
396	GRZN2-RUS	24552790***	*Ovis aries*	Grosny, Russia	0	43.32	45.7	This study, PCR	B	mCC
397	KALM1-RUS	24552790***	*Ovis aries*	Kalmykia, Russia	0	46.81	45.66	This study, PCR	B	mCC
398	KALM2-RUS	24552790***	*Ovis aries*	Kalmykia, Russia	0	46.81	45.66	This study, PCR	B	mCC
399	KRCH1-RUS	KF938351.1	*Ovis aries*	Karachay-Cherkessia, Russia	0	44.22	42.05	(30)	B	mCC
400	KRCH2-RUS	24552790***	*Ovis aries*	Karachay-Cherkessia, Russia	0	44.22	42.05	This study, PCR	A	mCC
401	KRCH3-RUS	24552790***	*Ovis aries*	Karachay-Cherkessia, Russia	0	44.22	42.05	This study, PCR	B	mCC
402	LEZG1-RUS	24552790***	*Ovis aries*	Dagestan, Russia	0	43.04	46.92	This study, PCR	A	mCC
403	LEZG2-RUS	24552790***	*Ovis aries*	Dagestan, Russia	0	43.04	46.92	This study, PCR	A	mCC
404	MANM1-RUS	24552790***	*Ovis aries*	Manychskaya, Oblast Rostow, Russia	0	47.23	40.25	This study, PCR	B	mCC
405	MANM2-RUS	24552790***	*Ovis aries*	Manychskaya, Oblast Rostow, Russia	0	47.23	40.25	This study, PCR	B	mCC
406	NCSN1-RUS	24552790***	*Ovis aries*	Stavropol, Russia	0	45.04	41.98	This study, PCR	B	mCC
407	NCSN2-RUS	24552790***	*Ovis aries*	Stavropol, Russia	0	45.04	41.98	This study, PCR	B	mCC
408	SALS1-RUS	24552790***	*Ovis aries*	Rostov Oblast, Russia	0	47.75	41.11	This study, PCR	B	mCC
409	SALS2-RUS	24552790***	*Ovis aries*	Rostov Oblast, Russia	0	47.75	41.11	This study, PCR	B	mCC
410	SOVM1-RUS	24552790***	*Ovis aries*	Southern Russia	0	45.57	43.08	This study, PCR	B	mCC
411	SOVM2-RUS	24552790***	*Ovis aries*	Southern Russia	0	45.57	43.08	This study, PCR	B	mCC
412	STAV1-RUS	24552790***	*Ovis aries*	Stavropol, Russia	0	45.04	41.98	This study, PCR	B	mCC
413	STAV2-RUS	24552790***	*Ovis aries*	Stavropol, Russia	0	45.04	41.98	This study, PCR	A	mCC
414	VOLG1-RUS	24552790***	*Ovis aries*	Volgograd, Russia	0	48.66	44.46	This study, PCR	A	mCC
415	VOLG2-RUS	24552790***	*Ovis aries*	Volgograd, Russia	0	48.66	44.46	This study, PCR	B	mCC
416	GLA1-AZE	KF938345.1	*Ovis aries*	Absheron region, Azerbaijan	0	40.39	49.29	(30)	A	mCC
417	MZK1-AZE	KF938344.1	*Ovis aries*	Arran, Azerbaijan	0	40.74	47.09	(30)	B	mCC
418	AFS1-IRN	SRS335703	*Ovis aries*	Afshari, Iran	0	35.79	51.47	SRA	B	mCC
419	AFS2-IRN	SRS335685	*Ovis aries*	Afshari, Iran	0	35.79	51.47	SRA	C	mCC
420	OGM02-IRN	ERS154869	*Ovis aries*	Ghare ziadin, Iran	0	38.859	45.061	SRA	B	mCC
421	OGM03-IRN	ERS154866	*Ovis aries*	Ajabshir, Iran	0	37.470	45.877	SRA	C	mCC
422	OGM04-IRN	ERS154867	*Ovis aries*	Ahar, Iran	0	38.523	46.851	SRA	C	mCC
423	OGM05-IRN	ERS154868	*Ovis aries*	Bostan abad, Iran	0	37.914	46.613	SRA	A	mCC
424	OGM06-IRN	ERS154865	*Ovis aries*	Aalmas, Iran	0	38.153	44.839	SRA	A	mCC
425	OGM07-IRN	ERS154864	*Ovis aries*	Ilbolaghi, Iran	0	39.045	44.928	SRA	A	mCC
426	OGM10-IRN	ERS154862	*Ovis aries*	Niaz, Iran	0	38.393	47.431	SRA	A	mCC
427	OGM11-IRN	ERS154863	*Ovis aries*	Urumie, Iran	0	37.973	44.953	SRA	B	mCC
428	HMD01-IRQ	MF004242	*Ovis aries*	Kurdistan region, Iraq	0	36.20	44.01	(37)	A	mCC
429	HMD02-IRQ	MF004243	*Ovis aries*	Kurdistan region, Iraq	0	36.20	44.01	(37)	A	mCC
430	HMD03-IRQ	MF004244	*Ovis aries*	Kurdistan region, Iraq	0	36.20	44.01	(37)	A	mCC
431	KRD01-IRQ	MF004245	*Ovis aries*	Kurdistan region, Iraq	0	36.20	44.01	(37)	B	mCC
432	KRD02-IRQ	MF004246	*Ovis aries*	Kurdistan region, Iraq	0	36.20	44.01	(37)	B	mCC
433	KRS1-TUR	HM236176.1	*Ovis aries*	Van, Eastern Anatolia, Türkiye	0	38.51	43.36	(31)	B	mCC
434	KRS2-TUR	HM236177.1	*Ovis aries*	Van, Eastern Anatolia, Türkiye	0	38.51	43.36	(31)	B	mCC
435	KRS3-TUR	HM236178.1	*Ovis aries*	Van, Eastern Anatolia, Türkiye	0	38.51	43.36	(31)	C	mCC
436	KRS4-TUR	SRS335719	*Ovis aries*	Van, Eastern Anatolia, Türkiye	0	38.51	43.36	SRA	C	mCC
437	MKR1-TUR	HM236179.1	*Ovis aries*	Eastern Anatolia, Türkiye	0	39.95	41.27	(31)	C	mCC

438	MKR2-TUR	HM236180.1	*Ovis aries*	Eastern Anatolia, Türkiye	0	39.95	41.27	(31)	D	mCC
439	MKR3-TUR	HM236181.1	*Ovis aries*	Eastern Anatolia, Türkiye	0	39.95	41.27	(31)	D	mCC
440	NDZ1-TUR	SRS335721	*Ovis aries*	Van, Eastern Anatolia, Türkiye	0	38.51	43.36	SRA	A	mCC
441	NDZ2-TUR	SRS335701	*Ovis aries*	Van, Eastern Anatolia, Türkiye	0	38.51	43.36	SRA	A	mCC
442	TUJ1-TUR	HM236183.1	*Ovis aries*	Çildir, (most Nort-East) Türkiye	0	41.13	43.14	(31)	E	mCC
443	KARA1-UZB	KF938348.1	*Ovis aries*	Qorako'l, Uzbekistan	0	47.28	48.03	(30)	B	mCC
444	KARA2-UZB	24552790***	*Ovis aries*	Qorako'l, Uzbekistan	0	47.28	48.03	This study	A	mCC
445	KARA3-UZB	24552790***	*Ovis aries*	Qorako'l, Uzbekistan	0	47.28	48.03	This study	B	mCC
446	AWS1-ISR	HM236182.1	*Ovis aries*	Syro-Arabian Desert, Syria	0	34.23	38.75	(31)	E	mLEV
447	AWS2-ISR	SRS152492	*Ovis aries*	Syro-Arabian Desert, Syria	0	34.23	38.75	SRA	B	mLEV
448	AWS3-ISR	SRS335723	*Ovis aries*	Syro-Arabian Desert, Syria	0	34.23	38.75	SRA	A	mLEV
449	AWS4-ISR	SRS004311	*Ovis aries*	Syro-Arabian Desert, Syria	0	34.23	38.75	SRA	B	mLEV
450	AWS5-TUR	SRS335726	*Ovis aries*	Syro-Arabian Desert, Syria	0	34.23	38.75	SRA	B	mLEV
451	AWS6-TUR	SRS335704	*Ovis aries*	Syro-Arabian Desert, Syria	0	34.23	38.75	SRA	B	mLEV
452	ASF1-ISR	EF490456.1	*Ovis aries*	Jerusalem, Israel	0	31.77	35.21	(34)	B	mLEV
453	ASF2-ISR	HE577847.1	*Ovis aries*	Jerusalem, Israel	0	31.77	35.21	(39)	A	mLEV
454	ASF3-ISR	HE577848.1	*Ovis aries*	Jerusalem, Israel	0	31.77	35.21	(39)	B	mLEV
455	ASF4-ISR	HE577849.1	*Ovis aries*	Jerusalem, Israel	0	31.77	35.21	(39)	B	mLEV
456	ASF5-ISR	HE577850.1	*Ovis aries*	Jerusalem, Israel	0	31.77	35.21	(39)	C	mLEV
457	aDZHU1-BGR	24552790***	*Ovis aries*	Dzhulyunitsa, Bulgaria	7230	43.09	25.55	This study	B	aEU
458	aDZHU2-BGR	24552790***	*Ovis aries*	Dzhulyunitsa, Bulgaria	7230	43.09	25.55	This study	B	aEU
459	aFRAN1-DEU	24552790***	*Ovis aries*	Frankfurt-Heddern, Germany	1630	50.15	8.64	This study	A	aEU
460	aMAIN4-DEU	24552790***	*Ovis aries*	Meinz, Germany	1730	50.00	8.25	This study	B	aEU
461	aALZE1-DEU	24552790***	*Ovis aries*	Alzey, Germany	1630	49.75	8.11	This study	B	aEU
462	aMAIN1-DEU	24552790***	*Ovis aries*	Meinz, Germany	1730	50.00	8.25	This study	B	aEU
463	aMAIN2-DEU	24552790***	*Ovis aries*	Meinz, Germany	1730	50.00	8.25	This study	B	aEU
464	aMAIN3-DEU	24552790***	*Ovis aries*	Meinz, Germany	1730	50.00	8.25	This study	B	aEU
465	aHERX7-DEU	24552790***	*Ovis aries*	Herxheim, Germany	6930	49.14	8.21	This study	B	aEU
466	aHERX8-DEU	24552790***	*Ovis aries*	Herxheim, Germany	6930	49.14	8.21	This study	B	aEU
467	aSILG1-GBR	24552790***	*Ovis aries*	Silgenach, Scotland	3930	57.10	-7.35	This study	B	aEU
468	OAA73-GBR	ERS426703	*Ovis aries*	York, UK	247	53.95	-1.05	SRA	B	aEU
469	OAA74-GBR	ERS426702	*Ovis aries*	York, UK	247	53.95	-1.05	SRA	B	aEU
470	aORKN2-GBR	24552790***	*Ovis aries*	Orkney, Scotland, UK	930	59.00	-3.11	This study	B	aEU
471	aHUNG1-GBR	24552790***	*Ovis aries*	York, England, UK	730	53.95	-1.08	This study	B	aEU
472	aORKN1-GBR	24552790***	*Ovis aries*	Orkney, Scotland, UK	830	59.00	-3.11	This study	B	aEU
473	aKILP2-GBR	24552790***	*Ovis aries*	Kilpheder, Scotland, UK	830	57.15	-7.40	This study	B	aEU
474	aKILP3-GBR	24552790***	*Ovis aries*	Kilpheder, Scotland, UK	830	57.15	-7.40	This study	B	aEU
475	aBORN1-GBR	24552790***	*Ovis aries*	Bornais, Scotland, UK	930	57.24	-7.40	This study	B	aEU
476	aDANE1-GBR	24552790***	*Ovis aries*	Danebury, England, UK	2430	51.10	-1.53	This study	B	aEU
477	aDANE2-GBR	24552790***	*Ovis aries*	Danebury, England, UK	2430	51.10	-1.53	This study	B	aEU
478	aDANE3-GBR	24552790***	*Ovis aries*	Danebury, England, UK	2430	51.10	-1.53	This study	B	aEU
479	aHALL1-GBR	24552790***	*Ovis aries*	Cladh Hallan, South Uist, Scotland, UK	2930	57.10	-7.41	This study	B	aEU
480	aHALL2-GBR	24552790***	*Ovis aries*	Cladh Hallan, South Uist, Scotland, UK	2870	57.10	-7.41	This study	B	aEU
481	aHALL3-GBR	24552790***	*Ovis aries*	Cladh Hallan, South Uist, Scotland, UK	2730	57.10	-7.41	This study	B	aEU
482	aHALL4-GBR	24552790***	*Ovis aries*	Cladh Hallan, South Uist, Scotland, UK	3190	57.10	-7.41	This study	B	aEU
483	aHALL5-GBR	24552790***	*Ovis aries*	Cladh Hallan, South Uist, Scotland, UK	3190	57.10	-7.41	This study	B	aEU
484	aPOTT2-GBR	24552790***	*Ovis aries*	Potterne, England, UK	2430	51.32	-2.00	This study	B	aEU
485	aBROD1-GBR	24552790***	*Ovis aries*	Ness of Brodgar, Scotland, UK	4456	59.00	-3.22	This study	B	aEU
486	aBROD2-GBR	24552790***	*Ovis aries*	Ness of Brodgar, Scotland, UK	4430	59.00	-3.22	This study	B	aEU
487	aDUBL1-IRL	24552790***	*Ovis aries*	Moynagh Crannog, Meath, Ireland	880	53.35	-6.33	This study	B	aEU
488	aDUBL2-IRL	24552790***	*Ovis aries*	Fishamble Street, Dublin, Ireland	830	53.35	-6.33	This study	B	aEU
489	aDUBL3-IRL	24552790***	*Ovis aries*	Fishamble Street, Dublin, Ireland	830	53.35	-6.33	This study	B	aEU
490	aTARX3-MLT	24552790***	*Ovis aries*	Tarxien, Malta	4930	35.87	14.51	This study	B	aEU
491	aBLG04-SRB	24552790***	*Ovis aries*	Blagotin, Serbia	8155	43.73	21.11	This study	B	aEU
492	aBLG06-SRB	24552790***	*Ovis aries*	Blagotin, Serbia	8155	43.73	21.11	This study	B	aEU
493	aBLG07-SRB	24552790***	*Ovis aries*	Blagotin, Serbia	8155	43.73	21.11	This study	B	aEU
494	aBLG08-SRB	24552790***	*Ovis aries*	Blagotin, Serbia	8155	43.73	21.11	This study	B	aEU
495	aBLG09-SRB	24552790***	*Ovis aries*	Blagotin, Serbia	8155	43.73	21.11	This study	B	aEU

496	aBLG10-SRB	24552790***	*Ovis aries*	Blagotin, Serbia	8155	43.73	21.11	This study	B	aEU
497	aBLG11-SRB	24552790***	*Ovis aries*	Blagotin, Serbia	8155	43.73	21.11	This study	B	aEU
498	aBLG13-SRB	24552790***	*Ovis aries*	Blagotin, Serbia	8155	43.73	21.11	This study	B	aEU
499	aBLG15-SRB	24552790***	*Ovis aries*	Blagotin, Serbia	8155	43.73	21.11	This study	B	aEU
500	aBLG17-SRB	24552790***	*Ovis aries*	Blagotin, Serbia	8155	43.73	21.11	This study	B	aEU
501	aBLG18-SRB	24552790***	*Ovis aries*	Blagotin, Serbia	8155	43.73	21.11	This study	B	aEU
502	aBLG20-SRB	24552790***	*Ovis aries*	Blagotin, Serbia	8155	43.73	21.11	This study	B	aEU
503	aBUBA1-SRB	24552790***	*Ovis aries*	Bubanj-Nevo Selo, Serbia	6296	43.29	21.84	This study	B	aEU
504	aGEO01-GEO	24552790***	*Ovis aries*	Tamara Fort, Kazbegi, Georgia	830	42.746	44.64	This study	B	aCC
505	aGEO02-GEO	24552790***	*Ovis aries*	Tamara Fort, Kazbegi, Georgia	830	42.746	44.64	This study	B	aCC
506	aGEO03-GEO	24552790***	*Ovis aries*	Tamara Fort, Kazbegi, Georgia	830	42.746	44.64	This study	B	aCC
507	aGEO04-GEO	24552790***	*Ovis aries*	Tamara Fort, Kazbegi, Georgia	830	42.746	44.64	This study	B	aCC
508	aGEO05-GEO	24552790***	*Ovis aries*	Tamara Fort, Kazbegi, Georgia	830	42.746	44.64	This study	A	aCC
509	aGEO06-GEO	24552790***	*Ovis aries*	Tamara Fort, Kazbegi, Georgia	830	42.746	44.64	This study	B	aCC
510	aGEO07-GEO	24552790***	*Ovis aries*	Tamara Fort, Kazbegi, Georgia	830	42.746	44.64	This study	A	aCC
511	aGEO08-GEO	24552790***	*Ovis aries*	Tamara Fort, Kazbegi, Georgia	830	42.746	44.64	This study	A	aCC
512	aGEO10-GEO	24552790***	*Ovis aries*	Tamara Fort, Kazbegi, Georgia	830	42.746	44.64	This study	A	aCC
513	aGEO11-GEO	24552790***	*Ovis aries*	Tamara Fort, Kazbegi, Georgia	830	42.746	44.64	This study	B	aCC
514	aGEO13-GEO	24552790***	*Ovis aries*	Tamara Fort, Kazbegi, Georgia	830	42.746	44.64	This study	C	aCC
515	aKZB02-GEO	24552790***	*Ovis aries*	Tamara Fort, Kazbegi, Georgia	830	42.746	44.64	This study	B	aCC
516	aKZB10-GEO	24552790***	*Ovis aries*	Tamara Fort, Kazbegi, Georgia	830	42.746	44.64	This study	A	aCC
517	aKZB11-GEO	24552790***	*Ovis aries*	Tamara Fort, Kazbegi, Georgia	830	42.746	44.64	This study	B	aCC
518	aYOQN1-ISR	24552790***	*Ovis aries*	Tel Yoqne'am, Israel	1130	32.63	35.11	This study	A	aLEV
519	aYOQN4-ISR	24552790***	*Ovis aries*	Tel Yoqne'am, Israel	1112	32.66	35.1	This study	B	aLEV
520	aMIQN1-ISR	24552790***	*Ovis aries*	Miqne, Israel	3330	31.78	34.85	This study	B	aLEV
521	aMIQN2-ISR	24552790***	*Ovis aries*	Miqne, Israel	3330	31.78	34.85	This study	A	aLEV
522	aMIQN3-ISR	24552790***	*Ovis aries*	Miqne, Israel	3330	31.78	34.85	This study	B	aLEV
523	aMIQN6-ISR	24552790***	*Ovis aries*	Miqne, Israel	3330	31.78	34.85	This study	A	aLEV
524	aMASO1-ISR	24552790***	*Ovis aries*	Masos, Israel	3430	31.78	34.85	This study	A	aLEV
525	aYOQN3-ISR	24552790***	*Ovis aries*	Tel Yoqne'am, Israel	1130	32.63	35.11	This study	A	aLEV
526	aSHIQ2-ISR	24552790***	*Ovis aries*	Shiqmim, Israel	3530	31.22	34.51	This study	B	aLEV
527	aSHIQ4-ISR	24552790***	*Ovis aries*	Shiqmim, Israel	3530	31.22	34.51	This study	B	aLEV
528	aMARM4-TUR	24552790***	*Ovis aries*	Yenikapı, Türkiye	7814	41.21	28.94	This study	B	aSWAN
529	aMARM5-TUR	24552790***	*Ovis aries*	Yenikapı, Türkiye	7814	41.21	28.94	This study	B	aSWAN
530	aMARM6-TUR	24552790***	*Ovis aries*	Yenikapı, Türkiye	7814	41.21	28.94	This study	B	aSWAN
531	aMARM7-TUR	24552790***	*Ovis aries*	Yenikapı, Türkiye	7882	41.21	28.94	This study	B	aSWAN
532	aMARM8-TUR	24552790***	*Ovis aries*	Yenikapı, Türkiye	7882	41.21	28.94	This study	B	aSWAN
533	aMNT01-TUR	24552790***	*Ovis aries*	Menteşe, Türkiye	7580	40.30	29.53	This study	B	aSWAN
534	aMNT02-TUR	24552790***	*Ovis aries*	Menteşe, Türkiye	7580	40.30	29.53	This study	B	aSWAN
535	aMNT04-TUR	24552790***	*Ovis aries*	Menteşe, Türkiye	7580	40.30	29.53	This study	B	aSWAN
536	aMNT05-TUR	24552790***	*Ovis aries*	Menteşe, Türkiye	7580	40.30	29.53	This study	B	aSWAN
537	aMNT06-TUR	24552790***	*Ovis aries*	Menteşe, Türkiye	7580	40.30	29.53	This study	B	aSWAN
538	aMNT07-TUR	24552790***	*Ovis aries*	Menteşe, Türkiye	7580	40.30	29.53	This study	B	aSWAN
539	aMNT09-TUR	24552790***	*Ovis aries*	Menteşe, Türkiye	7580	40.30	29.53	This study	B	aSWAN
540	aMNT11-TUR	24552790***	*Ovis aries*	Menteşe, Türkiye	7580	40.30	29.53	This study	B	aSWAN
541	aMARM1-TUR	24552790***	*Ovis aries*	Yenikapı, Türkiye	7882	41.21	28.94	This study	B	aSWAN
542	aMARM3-TUR	24552790***	*Ovis aries*	Yenikapı, Türkiye	7882	41.21	28.94	This study	B	aSWAN
543	aSUBE1-TUR	24552790***	*Ovis aries*	Suberde, Türkiye	7430	37.41	31.88	This study	B	aSWAN
544	aSUBE3-TUR	24552790***	*Ovis aries*	Suberde, Türkiye	7430	37.41	31.88	This study	B	aSWAN
545	aCUKU1-TUR	24552790***	*Ovis aries*	Çukuriçi Höyük, Türkiye	8430	37.9	27.35	This study	B	aSWAN
546	aGUV02-TUR	24552790***	*Ovis aries*	Güvercinkayası, Türkiye	6925	38.35	34.23	This study, WGS*	A	aGK
547	aGUV11-TUR	24552790***	*Ovis aries*	Güvercinkayası, Türkiye	6925	38.35	34.23	This study, WGS*	B	aGK
548	aGUV12-TUR	24552790***	*Ovis aries*	Güvercinkayası, Türkiye	6925	38.35	34.23	This study, WGS*	A	aGK
549	aGUV13-TUR	24552790***	*Ovis aries*	Güvercinkayası, Türkiye	6925	38.35	34.23	This study, WGS*	A	aGK
550	aGUV14-TUR	24552790***	*Ovis aries*	Güvercinkayası, Türkiye	6925	38.35	34.23	This study, WGS*	E	aGK
551	aGUV15-TUR	24552790***	*Ovis aries*	Güvercinkayası, Türkiye	6925	38.35	34.23	This study, WGS*	B	aGK
552	aGUV16-TUR	24552790***	*Ovis aries*	Güvercinkayası, Türkiye	6925	38.35	34.23	This study, WGS*	B	aGK
553	aGUV178-TUR	24552790***	*Ovis aries*	Güvercinkayası, Türkiye	6925	38.35	34.23	This study, WGS*	B	aGK

554	aGUV179-TUR	24552790***	*Ovis aries*	Güvercinkayası, Türkiye	6925	38.35	34.23	This study, WGS*	B	aGK
555	aGUV17-TUR	24552790***	*Ovis aries*	Güvercinkayası, Türkiye	6925	38.35	34.23	This study, WGS*	B	aGK
556	aGUV180-TUR	24552790***	*Ovis aries*	Güvercinkayası, Türkiye	6925	38.35	34.23	This study, WGS*	B	aGK
557	aGUV182-TUR	24552790***	*Ovis aries*	Güvercinkayası, Türkiye	6925	38.35	34.23	This study, WGS*	B	aGK
558	aGUV18-TUR	24552790***	*Ovis aries*	Güvercinkayası, Türkiye	6925	38.35	34.23	This study, WGS*	A	aGK
559	aGUV19-TUR	24552790***	*Ovis aries*	Güvercinkayası, Türkiye	6925	38.35	34.23	This study, WGS*	Z	aGK
560	aGUV20-TUR	24552790***	*Ovis aries*	Güvercinkayası, Türkiye	6925	38.35	34.23	This study, WGS*	B	aGK
561	aGUV21-TUR	24552790***	*Ovis aries*	Güvercinkayası, Türkiye	6925	38.35	34.23	This study, WGS*	B	aGK
562	aGUV22-TUR	24552790***	*Ovis aries*	Güvercinkayası, Türkiye	6925	38.35	34.23	This study, WGS*	B	aGK
563	aGUV23-TUR	24552790***	*Ovis aries*	Güvercinkayası, Türkiye	6925	38.35	34.23	This study, WGS*	B	aGK
564	aGUV24-TUR	24552790***	*Ovis aries*	Güvercinkayası, Türkiye	6925	38.35	34.23	This study, WGS*	A	aGK
565	aGUV25-TUR	24552790***	*Ovis aries*	Güvercinkayası, Türkiye	6925	38.35	34.23	This study, WGS*	B	aGK
566	OARG004-2B	24552790***	*Ovis gmelini/aries*	Asikli Höyük, Türkiye	9425	38.35	34.23	This study, capture	D	aAH1
567	OARG006-2B	24552790***	*Ovis gmelini/aries*	Asikli Höyük, Türkiye	9425	38.35	34.23	This study, capture	D	aAH1
568	OARG008-2B	24552790***	*Ovis gmelini/aries*	Asikli Höyük, Türkiye	9425	38.35	34.23	This study, capture	E	aAH1
569	OARG010-2B	24552790***	*Ovis gmelini/aries*	Asikli Höyük, Türkiye	9425	38.35	34.23	This study, capture	D	aAH1
570	OARG011-2B	24552790***	*Ovis gmelini/aries*	Asikli Höyük, Türkiye	9425	38.35	34.23	This study, capture	B	aAH1
571	OARG012-2B	24552790***	*Ovis gmelini/aries*	Asikli Höyük, Türkiye	9425	38.35	34.23	This study, capture	Z	aAH1
572	OARG045-2B	24552790***	*Ovis gmelini/aries*	Asikli Höyük, Türkiye	9425	38.35	34.23	This study, capture	D	aAH1
573	OARG066-2B	24552790***	*Ovis gmelini/aries*	Asikli Höyük, Türkiye	9425	38.35	34.23	This study, capture	B	aAH1
574	OARG067-2B	24552790***	*Ovis gmelini/aries*	Asikli Höyük, Türkiye	9425	38.35	34.23	This study, capture	D	aAH1
575	OARG072-2B	24552790***	*Ovis gmelini/aries*	Asikli Höyük, Türkiye	9425	38.35	34.23	This study, capture	B	aAH1
576	OARG088-2B	24552790***	*Ovis gmelini/aries*	Asikli Höyük, Türkiye	9425	38.35	34.23	This study, capture	B	aAH1
577	OARG019-2J	24552790***	*Ovis gmelini/aries*	Asikli Höyük, Türkiye	9625	38.35	34.23	This study, capture	E	aAH2
578	OARG021-2J	24552790***	*Ovis gmelini/aries*	Asikli Höyük, Türkiye	9625	38.35	34.23	This study, capture	D	aAH2
579	OARG022-2J	24552790***	*Ovis gmelini/aries*	Asikli Höyük, Türkiye	9625	38.35	34.23	This study, capture	Z	aAH2
580	OARG028-2J	24552790***	*Ovis gmelini/aries*	Asikli Höyük, Türkiye	9625	38.35	34.23	This study, capture	B	aAH2
581	OARG049-2E	24552790***	*Ovis gmelini/aries*	Asikli Höyük, Türkiye	9625	38.35	34.23	This study, capture	D	aAH2
582	OARG053-2D	24552790***	*Ovis gmelini/aries*	Asikli Höyük, Türkiye	9625	38.35	34.23	This study, capture	B	aAH2
583	OARG064-2J	24552790***	*Ovis gmelini/aries*	Asikli Höyük, Türkiye	9625	38.35	34.23	This study, capture	D	aAH2
584	OARG082-2J	24552790***	*Ovis gmelini/aries*	Asikli Höyük, Türkiye	9625	38.35	34.23	This study, capture	B	aAH2
585	OARG083-2F	24552790***	*Ovis gmelini/aries*	Asikli Höyük, Türkiye	9625	38.35	34.23	This study, capture	Z	aAH2
586	OARG085-2J	24552790***	*Ovis gmelini/aries*	Asikli Höyük, Türkiye	9625	38.35	34.23	This study, capture	B	aAH2
587	OARG089-2J	24552790***	*Ovis gmelini/aries*	Asikli Höyük, Türkiye	9625	38.35	34.23	This study, capture	B	aAH2
588	OARG095-2J	24552790***	*Ovis gmelini/aries*	Asikli Höyük, Türkiye	9625	38.35	34.23	This study, capture	B	aAH2
589	OARG097-2J	24552790***	*Ovis gmelini/aries*	Asikli Höyük, Türkiye	9625	38.35	34.23	This study, capture	B	aAH2
590	OARG098-2F	24552790***	*Ovis gmelini/aries*	Asikli Höyük, Türkiye	9625	38.35	34.23	This study, capture	A	aAH2
591	OARG138-2J	24552790***	*Ovis gmelini/aries*	Asikli Höyük, Türkiye	9625	38.35	34.23	This study, capture	B	aAH2
592	OARG105-3C	24552790***	*Ovis gmelini/aries*	Asikli Höyük, Türkiye	9850	38.35	34.23	This study, capture	B	aAH3
593	OARG106-3C	24552790***	*Ovis gmelini/aries*	Asikli Höyük, Türkiye	9850	38.35	34.23	This study, capture	D	aAH3
594	OARG107-3C	24552790***	*Ovis gmelini/aries*	Asikli Höyük, Türkiye	9850	38.35	34.23	This study, capture	A	aAH3
595	OARG111-3A	24552790***	*Ovis gmelini/aries*	Asikli Höyük, Türkiye	9850	38.35	34.23	This study, capture	D	aAH3
596	OARG112-3C	24552790***	*Ovis gmelini/aries*	Asikli Höyük, Türkiye	9850	38.35	34.23	This study, capture	D	aAH3
597	OARG129-3D	24552790***	*Ovis gmelini/aries*	Asikli Höyük, Türkiye	9850	38.35	34.23	This study, capture	A	aAH3
598	OARG130-3C	24552790***	*Ovis gmelini/aries*	Asikli Höyük, Türkiye	9850	38.35	34.23	This study, capture	Z	aAH3
599	OARG152-3D	24552790***	*Ovis gmelini/aries*	Asikli Höyük, Türkiye	9850	38.35	34.23	This study, capture	E	aAH3
600	OARG153-3D	24552790***	*Ovis gmelini/aries*	Asikli Höyük, Türkiye	9850	38.35	34.23	This study, capture	B	aAH3
601	OARG162-3D	24552790***	*Ovis gmelini/aries*	Asikli Höyük, Türkiye	9850	38.35	34.23	This study, capture	A	aAH3
602	OARG165-3D	24552790***	*Ovis gmelini/aries*	Asikli Höyük, Türkiye	9850	38.35	34.23	This study, capture	A	aAH3
603	OARG175-3D	24552790***	*Ovis gmelini/aries*	Asikli Höyük, Türkiye	9850	38.35	34.23	This study, WGS*	B	aAH3
604	OARG6004-3	24552790***	*Ovis gmelini/aries*	Asikli Höyük, Türkiye	9850	38.35	34.23	This study, WGS*	B	aAH3
605	OARG6134-3A	24552790***	*Ovis gmelini/aries*	Asikli Höyük, Türkiye	9850	38.35	34.23	This study, WGS*	B	aAH3
606	OARG040-4	24552790***	*Ovis gmelini/aries*	Asikli Höyük, Türkiye	10150	38.35	34.23	This study, capture	A	aAH4
607	OARG041-4	24552790***	*Ovis gmelini/aries*	Asikli Höyük, Türkiye	10150	38.35	34.23	This study, capture	D	aAH4
608	OARG075-4	24552790***	*Ovis gmelini/aries*	Asikli Höyük, Türkiye	10150	38.35	34.23	This study, capture	Z	aAH4
609	OARG077-4	24552790***	*Ovis gmelini/aries*	Asikli Höyük, Türkiye	10150	38.35	34.23	This study, capture	D	aAH4
610	OARG114-4	24552790***	*Ovis gmelini/aries*	Asikli Höyük, Türkiye	10150	38.35	34.23	This study, capture	B	aAH4
611	OARG117-4	24552790***	*Ovis gmelini/aries*	Asikli Höyük, Türkiye	10150	38.35	34.23	This study, capture	D	aAH4

612	OARG118-4	24552790***	*Ovis gmelini/aries*	Asikli Höyük, Türkiye	10150	38.35	34.23	This study, capture	A	aAH4
613	OARG120-4	24552790***	*Ovis gmelini/aries*	Asikli Höyük, Türkiye	10150	38.35	34.23	This study, capture	D	aAH4
614	OARG121-4	24552790***	*Ovis gmelini/aries*	Asikli Höyük, Türkiye	10150	38.35	34.23	This study, capture	A	aAH4
615	OARG125-4	24552790***	*Ovis gmelini/aries*	Asikli Höyük, Türkiye	10150	38.35	34.23	This study, capture	Z	aAH4
616	OARG126-4	24552790***	*Ovis gmelini/aries*	Asikli Höyük, Türkiye	10150	38.35	34.23	This study, capture	B	aAH4
617	OARG127-4	24552790***	*Ovis gmelini/aries*	Asikli Höyük, Türkiye	10150	38.35	34.23	This study, capture	A	aAH4
618	OARG132-4	24552790***	*Ovis gmelini/aries*	Asikli Höyük, Türkiye	10150	38.35	34.23	This study, capture	B	aAH4
619	OARG134-4	24552790***	*Ovis gmelini/aries*	Asikli Höyük, Türkiye	10150	38.35	34.23	This study, capture	D	aAH4
620	OARG145-5	24552790***	*Ovis gmelini/aries*	Asikli Höyük, Türkiye	10150	38.35	34.23	This study, capture	E	aAH4**
621	OARG149-4	24552790***	*Ovis gmelini/aries*	Asikli Höyük, Türkiye	10150	38.35	34.23	This study, capture	B	aAH4
622	OARG151-4	24552790***	*Ovis gmelini/aries*	Asikli Höyük, Türkiye	10150	38.35	34.23	This study, capture	D	aAH4
623	OARG156-4	24552790***	*Ovis gmelini/aries*	Asikli Höyük, Türkiye	10150	38.35	34.23	This study, capture	D	aAH4
624	OARG157-4	24552790***	*Ovis gmelini/aries*	Asikli Höyük, Türkiye	10150	38.35	34.23	This study, capture	Z	aAH4
625	OARG164-4	24552790***	*Ovis gmelini/aries*	Asikli Höyük, Türkiye	10150	38.35	34.23	This study, capture	B	aAH4
626	OARG167-4	24552790***	*Ovis gmelini/aries*	Asikli Höyük, Türkiye	10150	38.35	34.23	This study, capture	D	aAH4
627	OARGM501-5	24552790***	*Ovis gmelini/aries*	Asikli Höyük, Türkiye	10150	38.35	34.23	This study, WGS*	A	aAH4**
628	aKORT1-TUR	24552790***	*Ovis gmelini*	Körtik Tepe, Türkiye	10730	37.81	40.99	This study	B	aKT
629	aKORT3-TUR	24552790***	*Ovis gmelini*	Körtik Tepe, Türkiye	10730	37.81	40.99	This study	R	aKT

aut. pref. = autonomous prefecture
* WGS = whole genome sequencing
** These organisms are technically from layer 5 but the stratigraphic dating doesn’t allow to separate them from layer 4.
*** Sequences jointly provided in a common alignment at publicly available at FigShare.

Table S6. Temporal tests of haplogroup frequencies. The displayed values correspond to P-values of the Bayesian, Waples, and chi-squared tests, as well as to estimated values of two statistics: ordinary F_{ST} and temporal F_{STT} (67). The values in the last column are equal to 1.0 minus the proportion of simulated F_{STT}-values that were positive, which is a measure of statistical significance equivalent to a P-value. Acronyms correspond to: mEU = modern Europe; aEU = ancient Europe; mAFR = modern Africa; aSWAN = Neolithic southwestern Anatolia including Suberde, Çukuriçi, Menteşe and Marmara regions; aGK = Güvercinkayası; aAH = Aşıklı Höyük; aAH1 = Aşıklı Höyük, layers 2A-C; aAH2 = layers 2D-J of aAH: aAH3 = layers 3A-E of aAH; aAH4 = layer 4 A-C of aAH; mLEV = modern Levant; aLEV = ancient Levant; mCC = modern Caucasus; aCC = ancient Caucasus; mEASIA = modern eastern Asia. Color scale indicates significance (scale is shown under the table).

Comparison	t_1-t_0	N_e	Bayesian Test	Waples Test	Chi Test	F_{ST}	F_{STT}	1-Positive F_{STT}
aAH4-aAH3	100	10^2	0.977288	0.990992	0.792626	0.015803	-0.199897	0.993615
		10^3	0.747533	0.916390	0.792626	0.015803	-0.038577	0.851157
		10^4	0.584055	0.815503	0.792626	0.015803	-0.016782	0.704389
		10^5	0.558478	0.795114	0.792626	0.015803	-0.014553	0.678354
		10^6	0.55565	0.792880	0.792626	0.015803	-0.014357	0.675983
aAH3-aAH2	75	10^2	0.927992	0.972753	0.592786	0.029150	-0.156416	0.952526
		10^3	0.637660	0.792709	0.592786	0.029150	-0.024773	0.660932
		10^4	0.508821	0.624751	0.592786	0.029150	-0.008439	0.504183
		10^5	0.491944	0.596178	0.592786	0.029150	-0.006804	0.483406
		10^6	0.489673	0.593131	0.592786	0.029150	-0.006592	0.480552
aAH2-aAH1	67	10^2	0.746034	0.946317	0.624499	0.037367	-0.135625	0.890604
		10^3	0.396955	0.752104	0.624499	0.037367	-0.019663	0.550036
		10^4	0.297941	0.642235	0.624499	0.037367	-0.005295	0.424838
		10^5	0.285896	0.626347	0.624499	0.037367	-0.003767	0.408929
		10^6	0.284582	0.624687	0.624499	0.037367	-0.003646	0.407423
aAH1-aGK	833	10^2	0.470680	0.463031	0.010028	0.133071	-0.201935	0.908897
		10^3	0.353487	0.419814	0.010028	0.133071	-0.065403	0.608901
		10^4	0.035997	0.118342	0.010028	0.133071	0.076156	0.075778
		10^5	0.008086	0.017598	0.010028	0.133071	0.094441	0.021874
		10^6	0.006421	0.010704	0.010028	0.133071	0.096238	0.018196
aAH1-aSWAN	569	10^2	0.319386	0.671164	0.001735	0.327586	-0.005822	0.458854
		10^3	0.087892	0.402698	0.001735	0.327586	0.181317	0.097279
		10^4	0.001894	0.029031	0.001735	0.327586	0.276968	0.002386
		10^5	0.000319	0.002867	0.001735	0.327586	0.288324	0.000454
		10^6	0.000293	0.001833	0.001735	0.327586	0.289519	0.000356
aAH-aGK	967	10^2	0.998752	0.958942	0.032364	0.071838	-0.258883	0.999985
		10^3	0.796474	0.912667	0.032364	0.071838	-0.128584	0.858674
		10^4	0.138660	0.371590	0.032364	0.071838	0.031320	0.141076
		10^5	0.015945	0.058564	0.032364	0.071838	0.052458	0.012847
		10^6	0.009609	0.034700	0.032364	0.071838	0.054609	0.007313
aAH-aSWAN	703	10^2	0.586906	0.773815	0.000115	0.272774	-0.057741	0.754597
		10^3	0.184308	0.517110	0.000115	0.272774	0.110316	0.158855
		10^4	0.000901	0.013971	0.000115	0.272774	0.237278	0.000153
		10^5	0.000007	0.000280	0.000115	0.272774	0.252582	0.000001
		10^6	0.000003	0.000127	0.000115	0.272774	0.254160	0.000001
		10^2	0.036321	0.070304	0.052107	0.157025	0.112455	0.039291
		10^3	0.105660	0.080032	0.052107	0.157025	0.088619	0.097620

aGK-aSWAN	264	10^4	0.018691	0.059880	0.052107	0.157025	0.124508	0.011278
		10^5	0.009680	0.053038	0.052107	0.157025	0.129516	0.005343
		10^6	0.009073	0.052203	0.052107	0.157025	0.130005	0.004828
aAH1-aCC	2865	10^2	0.335254	0.333042	0.014521	0.144040	-0.191559	0.894623
		10^3	0.337385	0.35558	0.014521	0.144040	-0.176420	0.860738
		10^4	0.109875	0.214366	0.014521	0.144040	0.035237	0.262645
		10^5	0.010726	0.038793	0.014521	0.144040	0.094625	0.038349
		10^6	0.005702	0.016611	0.014521	0.144040	0.101059	0.023348
aAH-aCC	2999	10^2	0.946000	0.201441	0.011347	0.072967	-0.258090	1.000000
		10^3	0.905916	0.363052	0.011347	0.072967	-0.242131	0.986614
		10^4	0.293602	0.215599	0.011347	0.072967	-0.018764	0.504295
		10^5	0.021394	0.032810	0.011347	0.072967	0.043015	0.059388
		10^6	0.007769	0.013109	0.011347	0.072967	0.049582	0.025314
aAH1-aLEV	2236	10^2	0.326469	0.164438	0.016688	0.176820	-0.157846	0.811705
		10^3	0.335496	0.178880	0.016688	0.176820	-0.129888	0.754166
		10^4	0.088457	0.095335	0.016688	0.176820	0.074376	0.164194
		10^5	0.015632	0.026390	0.016688	0.176820	0.121193	0.030709
		10^6	0.010567	0.017637	0.016688	0.176820	0.126116	0.021689
aAH-aLEV	2369	10^2	0.944467	0.872142	0.039355	0.090631	-0.240145	1.000000
		10^3	0.877202	0.856277	0.039355	0.090631	-0.210529	0.962259
		10^4	0.249853	0.412315	0.039355	0.090631	0.005328	0.356535
		10^5	0.035418	0.072899	0.039355	0.090631	0.054588	0.054865
		10^6	0.02092	0.042379	0.039355	0.090631	0.059701	0.032421
aGK-aCC	2032	10^2	0.913473	0.783379	0.519728	0.013034	-0.274736	0.998123
		10^3	0.889526	0.805982	0.519728	0.013034	-0.242027	0.982299
		10^4	0.687000	0.731600	0.519728	0.013034	-0.064884	0.830024
		10^5	0.51059	0.575831	0.519728	0.013034	-0.023334	0.678761
		10^6	0.474942	0.526499	0.519728	0.013034	-0.018862	0.641894
aGK-aLEV	1402	10^2	0.807821	0.947467	0.475291	0.042654	-0.243745	0.977658
		10^3	0.758741	0.928462	0.475291	0.042654	-0.187414	0.903367
		10^4	0.413835	0.709851	0.475291	0.042654	-0.029281	0.515479
		10^5	0.266304	0.513654	0.475291	0.042654	-3.46E-05	0.338944
		10^6	0.246397	0.479430	0.475291	0.042654	0.003066	0.314173
aSWAN-aCC	2296	10^2	0.009854	0.013191	0.008676	0.226277	0.211623	0.009854
		10^3	0.028632	0.018498	0.008676	0.226277	0.189011	0.030089
		10^4	0.045553	0.015401	0.008676	0.226277	0.158900	0.041681
		10^5	0.006310	0.010088	0.008676	0.226277	0.189285	0.004266
		10^6	0.003427	0.008835	0.008676	0.226277	0.193491	0.002216
aSWAN-aLEV	1666	10^2	0.004957	0.001456	0.000933	0.333333	0.325876	0.004957
		10^3	0.040184	0.002202	0.000933	0.333333	0.281854	0.039643
		10^4	0.019075	0.001610	0.000933	0.333333	0.270106	0.015866
		10^5	0.002677	0.001042	0.000933	0.333333	0.291092	0.002146
		10^6	0.001673	0.000945	0.000933	0.333333	0.293938	0.001332
aLEV-aCC	629	10^2	0.936086	0.670453	0.590095	0.014493	-0.304783	0.985459
		10^3	0.831622	0.668553	0.590095	0.014493	-0.158929	0.849264
		10^4	0.649254	0.618328	0.590095	0.014493	-0.045676	0.681156
		10^5	0.596209	0.593890	0.590095	0.014493	-0.031971	0.631008
		10^6	0.588899	0.590492	0.590095	0.014493	-0.030462	0.623672
aAH1-aEU	1772	10^2	0.326209	0.784224	1.33E-06	0.304853	-0.029480	0.538837
		10^3	0.226365	0.742120	1.33E-06	0.304853	0.047062	0.328066
		10^4	0.004819	0.183634	1.33E-06	0.304853	0.244236	0.007574
		10^5	0.000032	0.000408	1.33E-06	0.304853	0.272489	0.000051

		10^6	0.000019	2.84E-06	1.33E-06	0.304853	0.275872	0.000013
aAH-aEU	1906	10^2	0.663850	0.794760	7.20E-07	0.250141	-0.080813	0.860967
		10^3	0.468419	0.748442	7.20E-07	0.250141	-0.025705	0.574952
		10^4	0.01308	0.098409	7.20E-07	0.250141	0.195540	0.005145
		10^5	0.000000	1.27E-05	7.20E-07	0.250141	0.000000	1.000000
		10^6	0.000000	7.00E-07	7.20E-07	0.250141	0.000000	1.000000
aGK-aEU	939	10^2	0.647277	0.926195	0.002175	0.130352	-0.156280	0.760795
		10^3	0.243480	0.863911	0.002175	0.130352	-0.007463	0.307073
		10^4	0.025516	0.299533	0.002175	0.130352	0.094002	0.030355
		10^5	0.001687	0.012327	0.002175	0.130352	0.110150	0.002360
		10^6	0.001051	0.002752	0.002175	0.130352	0.111971	0.001193
aSWAN-aEU	1202	10^2	0.095088	0.516295	0.532846	0.010753	0.003143	0.095088
		10^3	0.138068	0.533853	0.532846	0.010753	-0.000607	0.137406
		10^4	0.271066	0.534541	0.532846	0.010753	-0.000508	0.249173
		10^5	0.287265	0.533250	0.532846	0.010753	0.001587	0.228659
		10^6	0.277245	0.532892	0.532846	0.010753	0.001958	0.210484
aCC-aEU	1093	10^2	0.026978	0.012666	0.000141	0.195894	0.158676	0.026978
		10^3	0.055692	0.012328	0.000141	0.195894	0.136505	0.057816
		10^4	0.013899	0.000737	0.000141	0.195894	0.156515	0.011085
		10^5	0.001012	0.000180	0.000141	0.195894	0.170393	0.000630
		10^6	0.000516	0.000144	0.000141	0.195894	0.172128	0.000305
aLEV-aEU	464	10^2	0.022342	0.004419	7.96E-06	0.297313	0.265737	0.022328
		10^3	0.036821	0.000476	7.96E-06	0.297313	0.239401	0.031808
		10^4	0.001832	1.89E-05	7.96E-06	0.297313	0.261199	0.001090
		10^5	0.000288	8.69E-06	7.96E-06	0.297313	0.266580	0.000165
		10^6	0.000237	8.03E-06	7.96E-06	0.297313	0.267191	0.000112
aAH1-mEU	3142	10^2	0.330874	0.602306	0.497187	0.221847	-0.112826	0.753474
		10^3	0.329992	0.608551	0.497187	0.221847	-0.084752	0.621747
		10^4	0.020594	0.268283	0.497187	0.221847	0.141577	0.061935
		10^5	0.000049	0.000234	0.497187	0.221847	0.189269	0.000866
		10^6	0.00002	0.014579	0.497187	0.221847	0.195550	0.000200
aAH-mEU	3276	10^2	0.891920	0.874108	0.422497	0.163739	-0.167134	0.998010
		10^3	0.835330	0.868614	0.422497	0.163739	-0.151982	0.947743
		10^4	0.088420	0.399955	0.422497	0.163739	0.087575	0.085454
		10^5	0.000010	0.000151	0.422497	0.163739	0.150241	0.000012
		10^6	0.000000	0.002584	0.422497	0.163739	0.000000	1.000000
aGK-mEU	2308	10^2	0.883917	0.972997	0.000131	0.044049	-0.242748	0.977847
		10^3	0.706501	0.969088	0.000131	0.044049	-0.177010	0.904047
		10^4	0.280232	0.804360	0.000131	0.044049	-0.015420	0.484236
		10^5	0.053496	0.146716	0.000131	0.044049	0.024165	0.135949
		10^6	0.023905	0.001626	0.000131	0.044049	0.028859	0.087156
aSWAN-mEU	2572	10^2	0.005099	0.107774	0.103436	0.069486	0.061880	0.005099
		10^3	0.158595	0.123086	0.103436	0.069486	-0.001492	0.156976
		10^4	0.372466	0.121218	0.103436	0.069486	0.011815	0.283566
		10^5	0.169280	0.110139	0.103436	0.069486	0.047683	0.071965
		10^6	0.107873	0.104370	0.103436	0.069486	0.052549	0.042558
aCC-mEU	277	10^2	0.622206	0.827680	9.31E-05	0.086857	-0.173453	0.804994
		10^3	0.174694	0.531602	9.31E-05	0.086857	0.016158	0.283068
		10^4	0.022850	0.056602	9.31E-05	0.086857	0.060168	0.066822
		10^5	0.010224	0.000894	9.31E-05	0.086857	0.065485	0.039525
		10^6	0.009308	0.000128	9.31E-05	0.086857	0.066039	0.036105
aLEV-mEU	906	10^2	0.677555	0.477447	0.001420	0.158670	-0.160135	0.828819

		10^3	0.202507	0.372778	0.001420	0.158670	0.017789	0.305784
		10^4	0.022270	0.066901	0.001420	0.158670	0.112022	0.052079
		10^5	0.004352	0.004199	0.001420	0.158670	0.127861	0.006564
		10^6	0.003750	0.001626	0.001420	0.158670	0.129428	0.003887
aEU-mEU	1370	10^2	0.031482	0.471236	0.032267	0.042238	0.011383	0.071660
		10^3	0.249084	0.419808	0.032267	0.042238	-0.039635	0.480456
		10^4	0.307139	0.138622	0.032267	0.042238	0.008062	0.324812
		10^5	0.080757	0.044465	0.032267	0.042238	0.032162	0.037775
		10^6	0.037468	0.033558	0.032267	0.042238	0.034995	0.015239
aAH1-mAFR	3142	10^2	0.334869	0.855281	7.91E-05	0.281298	-0.054227	0.600404
		10^3	0.334762	0.855281	7.91E-05	0.281298	-0.054386	0.600316
		10^4	0.333823	0.855281	7.91E-05	0.281298	-0.054022	0.599089
		10^5	0.334721	0.855281	7.91E-05	0.281298	-0.054051	0.599821
		10^6	0.334418	0.855281	7.91E-05	0.281298	-0.054229	0.600187
aAH-mAFR	3276	10^2	0.678118	0.602796	0.003069	0.229026	-0.101866	0.934657
		10^3	0.596076	0.686153	0.003069	0.229026	-0.088058	0.838479
		10^4	0.048778	0.254546	0.003069	0.229026	0.150167	0.037931
		10^5	0.000001	5.99E-05	0.003069	0.229026	0.214047	0.000000
		10^6	0.000000	4.47E-05	0.003069	0.229026	0.000000	1.000000
aGK-mAFR	2308	10^2	0.659855	0.917749	8.31E-05	0.111418	-0.176622	0.821122
		10^3	0.413085	0.918063	8.31E-05	0.111418	-0.111285	0.583799
		10^4	0.070127	0.670748	8.31E-05	0.111418	0.059346	0.097250
		10^5	0.001346	0.038819	8.31E-05	0.111418	0.090787	0.003492
		10^6	0.000529	0.000356	8.31E-05	0.111418	0.095242	0.000529
aSWAN-mAFR	2572	10^2	0.098344	0.637284	0.653683	0.017341	0.002516	0.179729
		10^3	0.113739	0.661091	0.653683	0.017341	0.000363	0.171230
		10^4	0.380829	0.661636	0.653683	0.017341	-0.010247	0.491491
		10^5	0.373541	0.656609	0.653683	0.017341	0.000142	0.405179
		10^6	0.342202	0.654083	0.653683	0.017341	0.002814	0.329213
aCC-mAFR	277	10^2	0.445435	0.735510	1.26E-05	0.171900	-0.09522	0.562753
		10^3	0.038705	0.359142	1.26E-05	0.171900	0.111404	0.059109
		10^4	0.000350	0.008747	1.26E-05	0.171900	0.145285	0.000894
		10^5	0.000185	5.53E-05	1.26E-05	0.171900	0.150225	0.000170
		10^6	0.000189	1.53E-05	1.26E-05	0.171900	0.150724	0.000157
aLEV-mAFR	906	10^2	0.501641	0.592598	1.12E-06	0.270045	-0.049791	0.501888
		10^3	0.099049	0.471216	1.12E-06	0.270045	0.148160	0.104064
		10^4	0.000939	0.046157	1.12E-06	0.270045	0.228161	0.001518
		10^5	0.000086	3.53E-05	1.12E-06	0.270045	0.240797	0.000054
		10^6	0.000086	1.15E-06	1.12E-06	0.270045	0.242335	0.000060
aEU-mAFR	1370	10^2	0.566425	0.497332	0.579934	0.004243	-0.032658	0.669383
		10^3	0.596336	0.578454	0.579934	0.004243	-0.030136	0.666617
		10^4	0.703526	0.584184	0.579934	0.004243	-0.016708	0.771546
		10^5	0.668180	0.581695	0.579934	0.004243	-0.006029	0.673814
		10^6	0.646175	0.580180	0.579934	0.004243	-0.003939	0.614856
aAH1-mCC	3142	10^2	0.478616	0.374354	2.85E-05	0.115731	-0.219699	0.945627
		10^3	0.551112	0.398985	2.85E-05	0.115731	-0.212710	0.935977
		10^4	0.182752	0.255549	2.85E-05	0.115731	0.013531	0.330918
		10^5	0.007781	0.010387	2.85E-05	0.115731	0.079803	0.029276
		10^6	0.002340	0.000103	2.85E-05	0.115731	0.086906	0.013313
aAH-mCC	3276	10^2	0.995744	0.082519	5.27E-06	0.054903	-0.276075	1.000000
		10^3	0.972375	0.210325	5.27E-06	0.054903	-0.263572	0.994099
		10^4	0.424749	0.137062	5.27E-06	0.054903	-0.030500	0.603735

		10^5	0.007215	0.004378	5.27E-06	0.054903	0.038161	0.021178
		10^6	0.000149	2.32E-05	5.27E-06	0.054903	0.045522	0.001048
aGK-mCC	2308	10^2	0.919439	0.682435	0.238651	0.013191	-0.275527	0.998430
		10^3	0.920239	0.723100	0.238651	0.013191	-0.254517	0.987026
		10^4	0.769819	0.677459	0.238651	0.013191	-0.058953	0.857860
		10^5	0.475893	0.452375	0.238651	0.013191	-0.009676	0.564356
		10^6	0.385074	0.273005	0.238651	0.013191	-0.004568	0.463164
aSWAN-mCC	2572	10^2	0.019847	0.054992	0.012921	0.207275	0.178630	0.019847
		10^3	0.089899	0.073938	0.012921	0.207275	0.102067	0.094538
		10^4	0.104172	0.060592	0.012921	0.207275	0.130294	0.061365
		10^5	0.002991	0.024994	0.012921	0.207275	0.182422	0.000569
		10^6	0.000733	0.014263	0.012921	0.207275	0.188006	0.000085
aCC-mCC	277	10^2	0.982824	0.940177	0.919529	0.002580	-0.297094	0.995907
		10^3	0.982984	0.940177	0.919529	0.002580	-0.297367	0.995951
		10^4	0.982668	0.940177	0.919529	0.002580	-0.297290	0.995816
		10^5	0.982859	0.940177	0.919529	0.002580	-0.297213	0.995889
		10^6	0.982800	0.940177	0.919529	0.002580	-0.297089	0.995903
aLEV-mCC	906	10^2	0.939951	0.794139	0.639517	0.022882	-0.298779	0.987936
		10^3	0.867331	0.799285	0.639517	0.022882	-0.183474	0.906128
		10^4	0.617755	0.734056	0.639517	0.022882	-0.030156	0.651658
		10^5	0.477699	0.657707	0.639517	0.022882	-0.010129	0.480103
		10^6	0.456084	0.641542	0.639517	0.022882	-0.008038	0.453931
aEU-mCC	1370	10^2	0.036986	0.013134	3.72E-05	0.180313	0.131317	0.036988
		10^3	0.118855	0.024297	3.72E-05	0.180313	0.063871	0.123160
		10^4	0.022734	0.002012	3.72E-05	0.180313	0.139223	0.012373
		10^5	0.000028	0.000123	3.72E-05	0.180313	0.167318	0.000011
		10^6	0.000001	4.35E-05	3.72E-05	0.180313	0.170273	0.000001
aAH1-mEASIA	3142	10^2	0.334447	0.156327	0.000172	0.166637	-0.169084	0.857416
		10^3	0.497643	0.176145	0.000172	0.166637	-0.167008	0.882101
		10^4	0.106302	0.099614	0.000172	0.166637	0.064364	0.189362
		10^5	0.002103	0.000509	0.000172	0.166637	0.131861	0.008525
		10^6	0.000315	6.40E-07	0.000172	0.166637	0.139072	0.003249
aAH-mEASIA	3276	10^2	0.946164	0.027848	0.000141	0.084524	-0.246514	1.000000
		10^3	0.920585	0.096936	0.000141	0.084524	-0.233994	0.985741
		10^4	0.324041	0.038782	0.000141	0.084524	-0.000177	0.395917
		10^5	0.001036	6.19E-05	0.000141	0.084524	0.069098	0.001995
		10^6	0.000000	1.44E-06	0.000141	0.084524	0.000000	1.000000
aGK-mEASIA	2308	10^2	0.567424	0.513515	0.002281	0.069994	-0.217600	0.939035
		10^3	0.703854	0.548116	0.002281	0.069994	-0.214118	0.934235
		10^4	0.274435	0.363645	0.002281	0.069994	-0.002571	0.376077
		10^5	0.023119	0.052637	0.002281	0.069994	0.048164	0.047678
		10^6	0.007720	0.004786	0.002281	0.069994	0.053450	0.021236
aSWAN-mEASIA	2572	10^2	0.004941	0.001582	6.88E-06	0.361502	0.346747	0.009926
		10^3	0.119767	0.002599	6.88E-06	0.361502	0.187185	0.151100
		10^4	0.015838	0.001542	6.88E-06	0.361502	0.281531	0.011277
		10^5	0.000036	0.000102	6.88E-06	0.361502	0.337787	0.000007
		10^6	0.000001	1.08E-05	6.88E-06	0.361502	0.343549	0.000001
aCC-mEASIA	277	10^2	0.946425	0.916346	0.346724	0.028520	-0.279528	0.980562
		10^3	0.573377	0.765592	0.346724	0.028520	-0.060193	0.708534
		10^4	0.259225	0.458453	0.346724	0.028520	-4.04E-05	0.348282
		10^5	0.200424	0.360183	0.346724	0.028520	0.006234	0.263517
		10^6	0.192803	0.348114	0.346724	0.028520	0.006895	0.253428

		10^2	0.985044	0.505658	0.432619	0.015091	-0.305050	0.986021
		10^3	0.946351	0.516151	0.432619	0.015091	-0.193215	0.923904
aLEV-mEASIA	906	10^4	0.821830	0.479467	0.432619	0.015091	-0.036883	0.719174
		10^5	0.741950	0.441132	0.432619	0.015091	-0.016622	0.587439
		10^6	0.727870	0.433557	0.432619	0.015091	-0.014586	0.566594
		10^2	0.479855	0.563700	0.080555	0.121951	-0.213723	0.912532
		10^3	0.467355	0.583356	0.080555	0.121951	-0.201989	0.887512
aAH1-mLEV	3142	10^4	0.221358	0.411244	0.080555	0.121951	0.003313	0.374138
		10^5	0.048115	0.139617	0.080555	0.121951	0.066777	0.085020
		10^6	0.031513	0.086617	0.080555	0.121951	0.073586	0.058117
		10^2	0.997321	0.116317	0.031742	0.070742	-0.260339	1.000000
		10^3	0.977088	0.261446	0.031742	0.070742	-0.248796	0.990589
aAH-mLEV	3276	10^4	0.500210	0.185761	0.031742	0.070742	-0.031462	0.573373
		10^5	0.108795	0.057278	0.031742	0.070742	0.034992	0.105613
		10^6	0.062594	0.034305	0.031742	0.070742	0.042108	0.055340
		10^2	0.962358	0.713717	0.609141	0.008116	-0.280020	0.998142
		10^3	0.947376	0.745873	0.609141	0.008116	-0.253676	0.990445
aGK-mLEV	2308	10^4	0.866015	0.708127	0.609141	0.008116	-0.078563	0.924135
		10^5	0.780602	0.634153	0.609141	0.008116	-0.033936	0.853323
		10^6	0.761868	0.612134	0.609141	0.008116	-0.029027	0.836184
		10^2	0.014940	0.062326	0.055223	0.142857	0.121035	0.014940
		10^3	0.016842	0.080596	0.055223	0.142857	0.121838	0.019188
aSWAN-mLEV	2572	10^4	0.07106	0.073536	0.055223	0.142857	0.083059	0.094857
		10^5	0.019231	0.059351	0.055223	0.142857	0.103793	0.024405
		10^6	0.012540	0.055699	0.055223	0.142857	0.107075	0.016251
		10^2	0.968103	0.689329	0.567959	0.019674	-0.277604	0.982964
		10^3	0.817921	0.667979	0.567959	0.019674	-0.083821	0.820023
aCC-mLEV	277	10^4	0.679465	0.592730	0.567959	0.019674	-0.029217	0.657525
		10^5	0.650599	0.570858	0.567959	0.019674	-0.023283	0.620683
		10^6	0.647907	0.568257	0.567959	0.019674	-0.022775	0.616699
		10^2	0.837098	0.543085	0.310591	0.061224	-0.259610	0.939662
		10^3	0.650889	0.536051	0.310591	0.061224	-0.149865	0.758152
aLEV-mLEV	906	10^4	0.312554	0.402944	0.310591	0.061224	-0.010495	0.402029
		10^5	0.226095	0.323742	0.310591	0.061224	0.008892	0.293303
		10^6	0.216339	0.311979	0.310591	0.061224	0.010911	0.280331

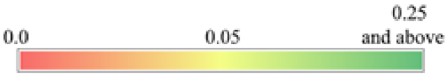

Table S7. Summary statistics and parameters of the ABC analyses. Names and descriptions of all summary statistics and parameters employed in the statistical inference by ABC. The number and letter codes in the second column are used to identify the exact sets of summary statistics and parameters used in every phase of analysis (shown in Tables S8, S10 and S12).

Type		Code	Short name	Description
Summary Statistics	Single-population	1	HapTypes	Number of different haplotypes in the sample
		2	PrivHaps	Number of haplotypes exclusive of the sample
		3	SegSites	Number of segregating sites in the sample alignment
		4	PairDiff	Average number of differences between all sequences' pairs of the sample
		5	NucDiver	Sample's nucleotide diversity
		6	GenDiver	Gene diversity
		7	TajimasD	Statistic *D* of Tajima
		8	FusFs	Statistic *Fs* of Fu
	Two-population	9	PairDifG	Average number of differences between sequences pairs of different samples
		10	Fst	Statistic of population divergence F_{ST}
		11	SharedHap	Number of haplotypes shared between 2 samples
		12	ShareFreq	Sum of minimum frequencies of haplotypes shared between 2 samples
Parameters		A	Ne	Effective population size of a terminal population
		B	Ev-Ne	Effective population size of an ancestral population
		C	Time	Time to an event (or most recent population delimiter)
		D	Mut_rate	Mutation rate in substitutions per site per year

Table S8. Summary statistics and parameters of ABC analyses, phase 1. The table below shows the combination of summary statistics and parameters of each model employed in the statistical inference by ABC, phase 1. Notice that all models require the same set of summary statistics to perform model choice but they differ in the number of parameters. The cells contain numbers and letters that correspond to the codes identifying summary statistics and parameters (brief guide under the table, full description in Table S7). The columns marked as 'large set', 'mid set', and 'small set' correspond to replicates of the analysis with different sets of summary statistics. The 'population/sample' labels serve to identify the summary statistics or parameters associated to statistical groups or 'populations' respectively. The actual number of summary statistics/parameters correspond to the sum of all combinations of a given column. For instance, the 'small set' of summary statistics included "PairDiff" of Güvercinkayası, "Haptypes" and "PairDiff" of the each of the four levels of Asikli Höyük, "PairDiff" of Körtik Tepe as well as "PairDifG" of the pairs aGK/aAH1, aAH1/aAH2, aAH2/aAH3, aAH3/aAH4, and aAH4/aKT giving an overall of 15 summary statistics, while the set of parameters of e.g. model 2 included "Ne" of the Güvercinkayası population, "time" and "Ne" of the population of Asikli Höyük 1-2, "time" and "Ne" of Asikli Höyük 3-4, "time" and "Ne" of Körtik Tepe, and "Mut_rate". Red text indicates the best supported model.

ABC phase 1		Summary Statistics*			Parameters**		
	Population/Sample	Large set	Mid set	Small set	Model 1	Model 2	Model 3
Single-population	Güvercinkayası	1,3,4,7,8	1,4,8	4	A	A	A
	Asikli Höyük 1	1,3,4,7,8	1,3,4,8	1,4	B,C	B,C	B,C
	Asikli Höyük 2	1,3,4,7,8	1,3,4,8	1,4			B,C
	Asikli Höyük 3	1,3,4,7,8	1,3,4,8	1,4		B,C	B,C
	Asikli Höyük 4	1,3,4,7,8	1,3,4,8	1,4			B,C
	Körtik Tepe	4	4	4	B,C	B,C	B,C
Two-population**	aGK/aAH1	9	9	9			
	aAH1/aAH2	9,10,11,12	9,10	9			
	aAH2/aAH3	9,10,11,12	9,11	9			
	aAH3/aAH4	9,10,11,12	9,12	9			
	aAH4/aKT	9	9	9			
Other					D	D	D

* 1=HapTypes; 2=PrivHaps; 3=SegSites; 4=PairDiff; 5=NucDiver; 6=GenDiver; 7=TajimasD; 8=FusFs; 9=PairDifG; 10=Fst; 11=SharedHap; 12=ShareFreq.
** A=Ne; B=Ev-Ne; C=Time; D=Mut_rate.
*** aGK=Güvercinkayası; aAH1=Asikli Höyük 1, aAH2=Asikli Höyük 2, etc.; aKT= Körtik Tepe.

Table S9. Model likelihoods of the ABC model choice, phase 1. The displayed model likelihoods correspond to rejection ratios adjusted by the Euclidean distance to the empirical data. The replicates of the post-simulation analysis used different sets of summary statistics as shown in Table S8. The column of random forests shows the number of forests selecting each model and the model likelihood of the selected model (recall that random forests-ABC does not estimate likelihoods for non-selected models). The range of Bayes factors considers the selected model (in red) as a reference.

Model	Brief description	Model likelihoods			Random forests
		Large set	Mid set	Small set	
1	No-change at Asikli Höyük	0.2045	0.1427	0.1591	234
2	Two-stages during Asikli Höyük's occupation	0.4024	0.5407	0.4565	405
3	Four-stages during Asikli Höyük's occupation	0.3931	0.3166	0.3843	361
	Bayes Factor range/RF-model likelihood	1.0-2.0	1.7-3.8	1.2-2.9	0.5854

Table S10. Summary statistics and parameters of ABC analyses, phase 2. The table below shows the combination of summary statistics and parameters of each model employed in the statistical inference by ABC, phase 2. Notice that all models require the same set of summary statistics to perform model choice but they differ in the number of parameters. The cells contain numbers and letters that correspond to the codes identifying summary statistics and parameters (brief guide under the table, full description in Table S7). The columns marked as '27-set', '24-set', etc. correspond to replicates of the analysis with different sets of summary statistics. The 'population/sample' labels serve to identify the summary statistics or parameters associated to statistical groups or 'populations' respectively. The actual number of summary statistics/parameters correspond to the sum of all combinations of a given column. Red text indicates the best supported models.

<table>
<tr><th colspan="2">ABC phase 2</th><th colspan="5">Summary Statistics*</th><th colspan="8">Parameters**</th></tr>
<tr><th></th><th>Population/Sample</th><th>27-set</th><th>24-set</th><th>21-set</th><th>15-set-T</th><th>15-set-F</th><th>Model A</th><th>Model B</th><th>Model C</th><th>Model D</th><th>Model E</th><th>Model F</th><th>Model G</th><th>Model H</th></tr>
<tr><td rowspan="6">Single population</td><td>Güvercinkayası</td><td>1,3,4,7,8</td><td>1,3,4,8</td><td>1,4,8</td><td>1,4,7</td><td>1,4,8</td><td>A</td><td>A</td><td>A</td><td>A</td><td>A</td><td>A</td><td>A</td><td>A</td></tr>
<tr><td>Neolithic southwestern Anatolia</td><td>1,3,4,7,8</td><td>1,3,4,8</td><td>1,4,8</td><td>1,4,7</td><td>1,4,8</td><td>B,C</td><td>A</td><td>A</td><td>A</td><td>B,C</td><td>A</td><td>A</td><td>A</td></tr>
<tr><td>Asikli Höyük</td><td>1,3,4,7,8</td><td>1,3,4,8</td><td>1,4,8</td><td>1,4,7</td><td>1,4,8</td><td>B,C</td><td>A</td><td>A</td><td rowspan="2">A</td><td rowspan="2">A</td><td rowspan="2">B,C</td><td rowspan="2">B,C</td><td rowspan="2">B,C</td></tr>
<tr><td>Körtik Tepe</td><td></td><td></td><td></td><td></td><td></td><td>B,C</td><td>B,C</td><td>B,C</td></tr>
<tr><td>Ancestor aGK/aSWAN</td><td></td><td></td><td></td><td></td><td></td><td></td><td></td><td></td><td>B,C</td><td></td><td></td><td></td><td></td></tr>
<tr><td>Ancestor population</td><td></td><td></td><td></td><td></td><td></td><td></td><td>B,C</td><td>B,C</td><td>B,C</td><td>B,C</td><td>B,C</td><td>B,C</td><td>B,C</td></tr>
<tr><td rowspan="6">Two-population</td><td>aGK/aSWAN</td><td>9,10</td><td>9,10</td><td>9,10</td><td>9</td><td>9</td><td></td><td></td><td></td><td></td><td></td><td></td><td></td><td></td></tr>
<tr><td>aGK/aAH</td><td>9,10</td><td>9,10</td><td>9,10</td><td>9</td><td>9</td><td></td><td></td><td></td><td></td><td></td><td></td><td></td><td></td></tr>
<tr><td>aGK/aKT</td><td>9,10</td><td>9,10</td><td>9,10</td><td>9</td><td>9</td><td></td><td></td><td></td><td></td><td></td><td></td><td></td><td></td></tr>
<tr><td>aSWAN/aAH</td><td>9,10</td><td>9,10</td><td>9,10</td><td>9</td><td>9</td><td></td><td></td><td></td><td></td><td></td><td></td><td></td><td></td></tr>
<tr><td>aSWAN/aKT</td><td>9,10</td><td>9,10</td><td>9,10</td><td>9</td><td>9</td><td></td><td></td><td></td><td></td><td></td><td></td><td></td><td></td></tr>
<tr><td>aAH/aKT</td><td>9,10</td><td>9,10</td><td>9,10</td><td>9</td><td>9</td><td></td><td></td><td></td><td></td><td></td><td></td><td></td><td></td></tr>
<tr><td colspan="2">Other</td><td></td><td></td><td></td><td></td><td></td><td>D</td><td>D</td><td>D</td><td>D</td><td>D</td><td>D</td><td>D</td><td>D</td></tr>
</table>

* 1=HapTypes; 2=PrivHaps; 3=SegSites; 4=PairDiff; 5=NucDiver; 6=GenDiver; 7=TajimasD; 8=FusFs; 9=PairDifG; 10=Fst; 11=SharedHap; 12=ShareFreq.

** A=Ne; B=Ev-Ne; C=Time; D=Mut_rate.

*** aGK=Güvercinkayası; aSWAN=Neolithic southwestern Anatolia; aAH=Asikli Höyük; aKT= Körtik Tepe.

Table S11. Model likelihoods of the ABC model choice, phase 2. The displayed model likelihoods correspond to rejection ratios adjusted by the Euclidean distance to the empirical data. The replicates of the post-simulation analysis used different sets of summary statistics as shown in Table S10. The column of random forests shows the number of forests selecting each model and the model likelihood of the selected model (recall that random forests-ABC does not estimate likelihoods for non-selected models). The range of Bayes factors considers the selected model (in red) as a reference. Parentheses in the second column summarize a genetic structure in Newick format while a '/' indicates belonging to the same continuous population e.g. 'aAH/aGK' indicates that Güvercinkayası descend from the Asikli Höyük population.

Model	Brief description	Model likelihoods					Random forests
		27-set	24-set	21-set	15-set-T	15-set-F	
A	Single continuous population	0.1563	0.1451	0.1291	0.1423	0.1645	158
B	((aAH,aSWAN),aGK)*	0.1532	0.1631	0.1591	0.1215	0.1504	38
C	((aAH,aGK),aSWAN)*	0.0541	0.0555	0.0551	0.0340	0.1104	6
D	(aAH,(aSWAN,aGK))*	0.1883	0.1934	0.2044	0.2060	0.159	243
E	(aAH,aSWAN/aGK)*	0.1556	0.0815	0.1401	0.1128	0.096	30
F	(aAH/aSWAN,aGK)*	0.1194	0.1113	0.1414	0.1017	0.1469	154
G	(aAH/aGK,aSWAN)*	0.0567	0.0914	0.0870	0.1349	0.0626	7
H	aAH/(aGK,aSWAN)*	0.1161	0.1711	0.1501	0.1465	0.1098	364
	Bayes Factor range/RF-model likelihood	1.2-3.5	1.0-4.5	1.1-5.4	1.4-6.0	1.0-2.6	0.6089

aGK=Güvercinkayası; aAH=Asikli Höyük, aSWAN=Neolithic southwestern Anatolia including Suberde, Çukuriçi, Menteşe and Marmara regions.

Table S12. Summary statistics and parameters of ABC analyses, phases 3-4. The cells contain numbers and letters indicating the summary statistics and parameters employed in the statistical inference by ABC, phases 3 and 4. The columns marked as ’47-set’, ’36-set’, etc. correspond to replicates of the analysis with the same simulations but different sets of summary statistics. The ‘population/sample’ labels serve to identify the summary statistics or parameters associated to statistical groups or ‘populations’ respectively. The actual number of summary statistics/parameters is the sum of all combinations of a given column. Red letters indicate the best supported model.

ABC phase 3		Summary Statistics*						Parameters**				
	Population/Sample	47-set	36-set	31-set	26-set-T	26-set-F	21-set	Model A	Model B	Model C	Model D	Model E
Single population	Modern Europe	1,3,4,7,8	1,3,4,7,8	1,4,7,8	1,4,7	1,4,8	1,4	A	A	A	A	A
	Ancient Europe	1,3,4,7,8	1,3,4,7,8	1,4,7,8	1,4,7	1,4,8	1,4					
	Güvercinkayası	1,3,4,7,8	1,3,4,7,8	1,4,7,8	1,4,7	1,4,8	1,4	A	A	A	B,C	A
	Neolithic southwestern Anatolia	1,3,4,7,8	1,3,4,7,8	1,4,7,8	1,4,7	1,4,8	1,4	A	A	B,C	A	A
	Asikli Höyük	1,3,4,7,8	1,3,4,7,8	1,4,7,8	1,4,7	1,4,8	1,4	A	A	A	A	A
	Körtik Tepe							B,C	B,C	B,C	B,C	B,C
	Ancestor Europe							B,C	B,C	B,C	B,C	B,C
	Ancestor aGK/aSWAN							B,C	B,C	B,C	B,C	B,C
	Ancestor population							B,C	B,C	B,C	B,C	B,C
Two-population	mEU/aEU	9,10	9	9	9	9	9					
	aEU/aGK	9,10	9	9	9	9	9					
	aEU/aSWAN	9,10	9	9	9	9	9					
	aEU/aAH	9,10	9	9	9	9	9					
	aEU/aKT	9,10	9	9	9	9	9					
	aGK/aSWAN	9,10	9	9	9	9	9					
	aGK/aAH	9,10	9	9	9	9	9					
	aGK/aKT	9,10	9	9	9	9	9					
	aSWAN/aAH	9,10	9	9	9	9	9					
	aSWAN/aKT	9,10	9	9	9	9	9					
	aAH/aKT	9,10	9	9	9	9	9					
Other								D	D	D	D	D

* Definition in Table S7 (above): 1=HapTypes; 2=PrivHaps; 3=SegSites; 4=PairDiffs; 5=NucDiver; 6=GenDiver; 7=TajimasD; 8=FusFs; 9=PairDiffs; 10=Fst; 11=SharedHap; 2=ShareFreq.

** Definition in Table S7 (above): A=Ne; B=Ev-Ne; C=Time; D=Mut_rate.

*** mEU=Modern Europe; aEU=ancient Europe; aGK=Güvercinkayası; aSWAN=Neolithic southwestern Anatolia; aAH=Asikli Höyük; aKT= Körtik Tepe.

Supplementary Table S13. Model likelihoods of the ABC model choice, phase 3. The displayed model likelihoods correspond to rejection ratios adjusted by the Euclidean distance to the empirical data. The replicates of the post-simulation analysis used different sets of summary statistics as shown in Table S12. The column of random forests shows the number of forests selecting each model and the model likelihood of the selected model (recall that random forests-ABC does not estimate likelihoods for non-selected models). The range of Bayes factors considers the selected model (in red) as a reference.

Model	Brief description	Model likelihoods						Random forests
		47-set	36-set	31-set	26-set-T	26-set-F	21-set	
A	EU from independent branch	0.0117	0.0112	0.0054	0.0041	0.0013	0.0003	240
B	EU descends from aAH	0.0142	0.0142	0.0086	0.0186	0.0055	0.0022	115
C	EU descends from aSWAN	0.3997	0.3962	0.3557	0.4177	0.4595	0.4321	38
D	EU descends from aGK	0.2954	0.3021	0.3192	0.2904	0.2844	0.2197	259
E	EU branch is sister to aGK & aSWAN	0.2787	0.2760	0.3108	0.2690	0.2491	0.3456	348
	Bayes Factor range/RF-model likelihood	1.4-34	1.3-29	1.1-65	1.4-102	1.6-342	1.3-1271	0.5054

EU=Europe, including both ancient and modern European samples; aGK=Güvercinkayası; aAH=Asikli Höyük; aSWAN=Neolithic southwestern Anatolia including Suberde, Çukuriçi, Menteşe and Marmara regions.

Supplementary Table S14. Contact information of new sheep samples. Inquiries regarding all new samples reported in this study, both modern and ancient ones, can be directed to the authors listed below. The samples they provided are indicated regarding their list number on Table S5.

Responsibe	Email	Samples (Table S5)
Andrea Zeeb-Lanz	zeeblanz@outlook.de	465-466
Barbara Horejs	Barbara.Horejs@oeaw.ac.at	545
Benjamin S Arbuckle	bsarbu@email.unc.edu	543-544
Canan Çakirlar	c.cakirlar@rug.nl	528-532, 541-542
Daniel Bradley	dbradley@tcd.ie	487-489
David Allen<br>Hampshire Cultural Trust	dave_allen99@hotmail.com	476-478
David Griffiths	david.griffiths@arch.ox.ac.uk	470-471, 472
David Orton	david.orton@york.ac.uk	491-502
Eberhard Sauer	esauer@exseed.ed.ac.uk	504-517
Fatmasevil Gülçur	sgulcur@istanbul.edu.tr	546-565
Gesine Lühken	gesine.luehken@agrar.uni-giessen.de	26-28, 40-42, 52-55, 82-92, 108-113, 117-120, 180-182, 189-198
Helmkut Hemmer	H.Hemmer-Mainz@gmx.de	461
Ivica Medugorac	ivica.medjugorac@gen.vetmed.uni-muenchen.de	29-38, 43-51, 56-75, 77-80, 93-96, 166-169, 199-200
Jelena Bulatović	jelena.bulatovic@gu.se	503
Joachim Burger	jburger@uni-mainz.de	459-460, 462-464
Lionel Gourichon	Lionel.GOURICHON@univ-cotedazur.fr	533-540
Liora Kolska Horwitz	lix1000@gmail.com	518-527
Lisa Brown<br>Wiltshire Museum	lisa.brown@wiltshiremuseum.org.uk	484
Mihriban Özbaşaran	ozbasaranmihriban@gmail.com	566-627
Mike Parker Pearson	m.parker-pearson@ucl.ac.uk	473-474, 479-483
Natalia Zinovieva	n_zinovieva@mail.ru	172-175, 177-178, 183-186, 373-376, 378-381, 383-386, 388-389, 391-398, 400-415, 444-445
Niall Sharples	sharples@cardiff.ac.uk	467, 475
Nick Card	nick.card@uhi.ac.uk	485-486
Petar Zidarov	petar.zidarov@gmail.com	457-458
Simon Stoddart	ss16@cam.ac.uk	490
Vecihi Özkaya	vozkaya@dicle.edu.tr	628-629

REFERENCES AND NOTES

1. H. Hongo, R. H. Meadow, Pig exploitation at Neolithic Çayönü Tepesi southeastern Anatolia, in *Ancestors for the Pigs: Pigs in Prehistory*, M. Nelson, Ed. (MASCA Research Papers in Science and Archaeology 15, Museum of Archaeology and Anthropology, Univ. of Pennsylvania, Philadelphia 1998), pp. 77–89.

2. J. Peters, A. von den Driesch, D. Helmer, M. S. Segui, Early animal husbandry in the Northern Levant. *Paléorient* **25**, 27–48 (1999).

3. J. Peters, A. von den Driesch, D. Helmer, in The Upper Euphrates-Tigris Basin: Cradle of agro-pastoralism?, in *The First Steps of Animal Domestication*, J.-D.Vigne, J. Peters, D. Helmer, Eds. (Oxbow Books, 2005), pp. 96–124.

4. K.-I. Tanno, G. Willcox, How fast was wild wheat domesticated? *Science* **311**, 1886 (2006).

5. E. Asouti, D. Q. Fuller, A contextual approach to the emergence of agriculture in Southwest Asia: Reconstructing early neolithic plant-food production. *Curr. Anthropol.* **54**, 299–345 (2013).

6. C. Kabukcu, E. Asouti, N. Pöllath, J. Peters, N. Karul, Pathways to plant domestication in Southeast Anatolia based on new data from aceramic Neolithic Gusir Höyük. *Sci. Rep.* **11**, 2112 (2021).

7. D. Baird, A. Fairbairn, E. Jenkins, L. Martin, C. Middleton, J. Pearson, E. Asouti, Y. Edwards, C. Kabukcu, G. Mustafaoğlu, N. Russell, O. Bar-Yosef, G. Jacobsen, X. Wu, A. Baker, S. Elliott, Agricultural origins on the Anatolian plateau. *Proc. Natl. Acad. Sci. U.S.A* **115**, E3077–E3086 (2018).

8. M. C. Stiner, N. D. Munro, H. Buitenhuis, G. Duru, M. Özbaşaran, An endemic pathway to sheep and goat domestication at Aşıklı Höyük (Central Anatolia, Turkey). *Proc. Natl. Acad. Sci. U.S.A* **119**, e2110930119 (2022).

9. S. Riehl, M. Zeidi, N. J. Conard, Emergence of agriculture in the foothills of the Zagros mountains of Iran. *Science* **341**, 65–67 (2013).

10. J. Conolly, S. Colledge, K. Dobney, J.-D. Vigne, J. Peters, B. Stopp, K. Manning, S. Shennan, Meta-analysis of zooarchaeological data from SW Asia and SE Europe provides insight into the origins and spread of animal husbandry. *J. Archaeol. Sci.* **38**, 538–545 (2011).

11. B. S. Arbuckle, S. W. Kansa, E. Kansa, D. Orton, C. Çakırlar, L. Gourichon, L. Atici, A. Galik, A. Marciniak, J. Mulville, H. Buitenhuis, D. Carruthers, B. De Cupere, A. Demirergi, S. Frame, D. Helmer, L. Martin, J. Peters, N. Pöllath, K. Pawłowska, N. Russell, K. Twiss, D. Würtenberger, Data sharing reveals complexity in the westward spread of domestic animals across Neolithic Turkey. *PLOS ONE* **9**, e99845 (2014).

12. J.-D. Vigne, I. Carrère, F. Briois, J. Guilaine, The early process of mammal domestication in the Near East: New evidence from the Pre-Neolithic and Pre-Pottery Neolithic in Cyprus. *Curr. Anthropol.* **52**, S255–S271 (2011).

13. J.-D. Vigne, L. Gourichon, D. Helmer, L. Martin, J. Peters, in *Quaternary of the Levant. Environments, Climate Change, and Humans*, E. Yehouda, B.-Y. Ofer, Eds. (Cambridge Univ. Press, 2017), pp. 753–760.

14. L. Gourichon, L. K. Horwitz, An inter-regional comparison of animal domestication in the northern and southern Levant. *Food Hist.* **19**, 53–78 (2021).

15. D. Q. Fuller, J. Van Etten, K. Manning, C. Castillo, E. Kingwell-Banham, A. Weisskopf, L. Qin, Y.-I Sato, R. J. Hijmans, The contribution of rice agriculture and livestock pastoralism to prehistoric methane levels:An archaeological assessment. *Holocene* **21**, 743–759 (2011).

16. H.-P. Uerpmann, *Probleme der Neolithisierung des Mittelmeeraums* (Beihefte zum Tübinger Atlas des Vordern Orients; Ludwing Reichert, 1979).

17. A. Ervynck, K. Dobney, H. Hongo, R. Meadow, Born Free? New evidence for the status of "*Sus scrofa*" at Neolithic Çayönü Tepesi (Southeastern Anatolia, Turkey). *Paléorient* **27**, 47–73 (2001).

18. J. Peters, F. Neuberger, I. Wiechmann, M. Zimmermann, M. Balasse, N. Pöllath, Shaping the sheep: Human management and decision-making at Aşıklı Höyük, Central Anatolia, in *The Early Settlement at Aşıklı Höyük. Essays in Honor of Ufuk Esin*, M. Özbaşaran, G. Duru, M. Stiner, Eds. (Ege Yayınları, 2018), pp. 325–344.

19. J. Peters, B. S. Arbuckle, N. Pöllath, Subsistence and beyond: Animals in Neolithic Anatolia, in *The Neolithic in Turkey. 10500–5200 BC: Environment, Settlement, Flora, Fauna, Dating, Symbols of belief, with Views from North, South, East, and West*, M. Özdoğan, N. Başgelen, P. Kuniholm, Eds. (Archaeology & Art Publications, 2014), pp. 135–203.

20. D. Helmer, Révision de la faune de Cafer Höyük (Malatya, Turquie). Apports des méthodes de l'analyse de mélanges et the l'analyse de Kernel à la mise en évidence de la domestication, in *Archaeozoology of the Near East VIII*, E. Vila, L. Gourichon, A. M. Choyke, H. Buitenhuis, Eds. (Travaux de la Maison de l'Orient et de la Méditerranée 49, 2008), pp. 169–196.

21. M. Özbaşaran, Re-starting at Aşıklı. *Anatolia antiqua. Eski Anadolu* **19**, 27–37 (2011).

22. M. Özbaşaran, Asikli Höyük, in *The Neolithic in Turkey. New Excavations and New Research: Central Turkey*, M. Özdoğan, N. Başgelen, P. Kuniholm, Eds. (Archaeology & Art Publications, 2012), pp. 135–158.

23. M. C. Stiner, H. Buitenhuis, G. Duru, S. L. Kuhn, S. M. Mentzer, N. D. Munro, N. Pöllath, J. Quade, G. Tsartsidou, M. Özbaşaran, A forager–herder trade-off, from broad-spectrum hunting to sheep management at Aşıklı Höyük, Turkey. *Proc. Natl. Acad. Sci. U.S.A* **111**, 8404–8409 (2014).

24. H. Buitenhuis, J. Peters, N. Pollath, M. C. Stiner, N. D. Munro, O. Sarıtaş, The faunal remains from levels 3 and 2 of Aşıklı Höyük: Evidence for emerging management

practices, in: *The Early Settlement at Aşıklı Höyük. Essays in Honor of Ufuk Esin*, M. Özbaşaran, G. Duru, M. C. Stiner Eds. (Ege Yayınları, 2018), pp. 281–323.

25. M. I. Zimmermann, N. Pöllath, M. Özbaşaran, J. Peters, Joint health in free-ranging and confined small bovids—Implications for early stage caprine management. *J. Archaeol. Sci.* **92**, 13–27 (2018).

26. S. M. Mentzer. Micromorphological analyses of anthropogenic materials and insight into tell formation processes at Aşıklı Höyük, in *The Early Settlement at Aşıklı Höyük. Essays in Honor of Ufuk Esin*, M. Özbaşaran, G. Duru, M. Stiner, Eds. (Ege Yayınları, 2018), pp. 105–128.

27. J T Abell, J. Quade, G. Duru, S M Mentzer, M C Stiner, M. Uzdurum, M. Özbaşaran, Urine salts elucidate Early Neolithic animal management at Aşıklı Höyük, Turkey. *Sci. Adv.* **5**, eaaw0038 (2019).

28. F. Neuberger, M. Balasse, N. Pöllath, J. Peters, Diet of wild versus culturally controlled caprines in Early Neolithic Anatolia based on stable carbon isotope analysis of bone apatite, in: *Animals: Cultural Identifiers in Ancient Societies*, J. Peters, G. McGlynn, V. Goebel, Eds. (Documenta Archaeobiologiae 15, M. Leidorf, 2019), pp. 251–260.

29. G. Tsartsidou, The microscopic record of Aşıklı Höyük: Phytolith analysis of material from the 2012–2016 field seasons, in *The Early Settlement at Aşıklı Höyük. Essays in Honor of Ufuk Esin*, M. Özbaşaran, G. Duru, M. Stiner, Eds. (Ege Yayınları, 2018), pp. 147–189.

30. F.-H. Lv, W.-F. Peng, J. Yang, Y.-X. Zhao, W.-R. Li, M.-J. Liu, Y.-H. Ma, Q.-J. Zhao, G.-L. Yang, F. Wang, J.-Q. Li, Y.-G. Liu, Z.-Q. Shen, S.-G. Zhao, E. Hehua, N. A. Gorkhali, S. M. F. Vahidi, M. Muladno, A. N. Naqvi, J. Tabell, T. Iso-Touru, M. W. Bruford, J. Kantanen, J.-L. Han, M.-H. Li, Mitogenomic meta-analysis identifies two phases of migration in the history of eastern eurasian sheep. *Mol. Biol. Evol.* **32**, 2515–2533 (2015).

31. J. R. S. Meadows, S. Hiendleder, J. W. Kijas, Haplogroup relationships between domestic and wild sheep resolved using a mitogenome panel. *Heredity* **106**, 700–706 (2011).

32. O. H. D. Brahi, H. Xiang, X. Chen, S. Farougou, X. Zhao, Mitogenome revealed multiple postdomestication genetic mixtures of West African sheep. *J. An. Breed. Genet.* **132**, 399–405 (2015).

33. S. Hiendleder, K. Mainz, Y. Plante, H. Lewalski, Analysis of mitochondrial DNA indicates that domestic sheep are derived from two different ancestral maternal sources: No evidence for contributions from urial and argali sheep. *J. Heredity* **89**, 113–120 (1998).

34. J. P. Burgstaller, P. Schinogl, A. Dinnyes, M. Müller, R. Steinborn, Mitochondrial DNA heteroplasmy in ovine fetuses and sheep cloned by somatic cell nuclear transfer. *BMC Dev. Biol.* **7**, 141 (2007).

35. H. Lancioni, Phylogenetic relationships of three italian merino-derived sheep breeds evaluated through a complete mitogenome analysis. *PLOS ONE* **8**, e73712 (2013).

36. X. Li, Whole-genome resequencing of wild and domestic sheep identifies genes associated with morphological and agronomic traits. *Nat. Commun.* **11**, 2815 (2020).

37. S. I. Mustafa, T. Schwarzacher, J. S. Heslop-Harrison, Complete mitogenomes from Kurdistani sheep: Abundant centromeric nuclear copies representing diverse ancestors. *Mitochondrial DNA Part A* **29**, 1180–1193 (2018).

38. Y. Niu, M. Jin, Y. Li, P. Li, J. Zhou, X. Wang, B. Petersen, X. Huang, Q. Kou, Y. Chen, Biallelic β-carotene oxygenase 2 knockout results in yellow fat in sheep via CRISPR/Cas9. *An. Genet.* **48**, 242–244 (2017).

39. S. Reicher, E. Seroussi, J. I. Weller, A. Rosov, E. Gootwine, Ovine mitochondrial DNA sequence variation and its association with production and reproduction traits within an Afec-Assaf flock1. *J. An. Sci.* **90**, 2084–2091 (2012).

40. J. Yang, W.-R. Li, F.-H. Lv, S.-G. He, S.-L. Tian, W.-F. Peng, Y.-W. Sun, Y.-X. Zhao, X.-L. Tu, M. Zhang, X.-L. Xie, Y.-T. Wang, J.-Q. Li, Y.-G. Liu, Z.-Q. Shen, F. Wang, G.-J. Liu, H.-F. Lu, J. Kantanen, J.-L. Han, M.-H. Li, M.-J. Liu, Whole-genome sequencing of native

sheep provides insights into rapid adaptations to extreme environments. *Molec. Biol. Evol.* **33**, 2576–2592 (2016).

41. C. Her, H.-R. Rezaei, S. Hughes, S. Naderi, M. Duffraisse, M. Mashkour, H.-R. Naghash, A. Bălăşescu, G. Luikart, S. Jordan, D. Özüt, A. Kence, M. W. Bruford, A. Tresset, J.-D. Vigne, P. Taberlet, C. Hänni, F. Pompanon, Broad maternal geographic origin of domestic sheep in Anatolia and the Zagros. *An. Genet.* **53**, 452–459 (2022).

42. E. Yurtman, O. Özer, E. Yüncü, N. D. Dağtaş, D. Koptekin, Y. G. Çakan, M. Özkan, A. Akbaba, D. Kaptan, G. Atağ, K. B. Vural, C. Y. Gündem, L. Martin, G. M. Kılınç, A. Ghalichi, S. C. Açan, R. Yaka, E. Sağlıcan, V. K. Lagerholm, M. Krzewińska, T. Günther, P. M. Miranda, E. Pişkin, M. Şevketoğlu, C C. Bilgin, Ç. Atakuman, Y. S. Erdal, E. Sürer, N. E. Altınışık, J. A. Lenstra, S. Yorulmaz, M. F. Abazari, J. Hoseinzadeh, D. Baird, E. Bıçakçı, Ö. Çevik, F. Gerritsen, R. Özbal, A. Götherström, M. Somel, İ. Togan, F. Özer, Archaeogenetic analysis of Neolithic sheep from Anatolia suggests a complex demographic history since domestication. *Commun. Biol.* **4**, 1279 (2021).

43. W. T. T. Taylor, M. Pruvost, C. Posth, W. Rendu, M. T. Krajcarz, A. Abdykanova, G. Brancaleoni, R. Spengler, T. Hermes, S. Schiavinato, G. Hodgins, R. Stahl, J. Min, S. A. Kyzy, S. Fedorowicz, L. Orlando, K. Douka, A. Krivoshapkin, C. Jeong, C. Warinner, S. Shnaider, Evidence for early dispersal of domestic sheep into Central Asia. *Nat. Hum. Behav.* **5**, 1169–1179 (2021).

44. J. R. S. Meadows, I. Cemal, O. Karaca, E. Gootwine, J. W. Kijas, Five ovine mitochondrial lineages identified from sheep breeds of the Near East. *Genetics* **175**, 1371–1379 (2007).

45. R. Kamalakkannan, S. Kumar, K. Bhavana, V. R. Prabhu, C. B. Machado, H. S. Singha, D. Sureshgopi, V. Vijay, M. Nagarajan, Evidence for independent domestication of sheep mtDNA lineage A in India and introduction of lineage B through Arabian sea route. *Sci. Rep.* **11**, 19733 (2021).

46. S. Pedrosa, M. Uzun, J.-J. Arranz, B. Gutiérrez-Gil, F. S. Primitivo, Y. Bayón, Evidence of three maternal lineages in Near Eastern sheep supporting multiple domestication events. *Proc. Biol. Sci.* **272**, 2211–2217 (2005).

47. S. Singh, S. Kumar Jr, A. P. Kolte, S. Kumar, Extensive variation and sub-structuring in lineage A mtDNA in Indian sheep: Genetic evidence for domestication of sheep in India. *PLOS ONE* **8**, e77858 (2013).

48. P. Gerbault, R. G. Allaby, N. Boivin, A. Rudzinski, I. M. Grimaldi, J. C. Pires, C. C. Vigueira, K. Dobney, K. J. Gremillion, L. Barton, M. Arroyo-Kalin, M. D. Purugganan, R. R. de Casas, R. Bollongino, J. Burger, D. Q. Fuller, D. G. Bradley, D. J. Balding, P. J. Richerson, M. T. P. Gilbert, G. Larson, M. G. Thomas, Storytelling and story testing in domestication. *Proc. Natl Acad. Sci. U S A* **111**, 6159 (2014).

49. L. Colli, H. Lancioni, I. Cardinali, A. Olivieri, M. R. Capodiferro, M. Pellecchia, M. Rzepus, W. Zamani, S. Naderi, F. Gandini, S. M. F. Vahidi, S. Agha, E. Randi, V. Battaglia, M. T. Sardina, B. Portolano, H. R. Rezaei, P. Lymberakis, F. Boyer, E. Coissac, F. Pompanon, P. Taberlet, P. A. Marsan, A. Achilli, Whole mitochondrial genomes unveil the impact of domestication on goat matrilineal variability. *BMC Genomics* **16,** 1115 (2015).

50. V. Cubric-Curik, D. Novosel, V. Brajkovic, O. R. Stabelli, S. Krebs, J. Sölkner, D. Šalamon, S. Ristov, B. Berger, S. Trivizaki, I. Bizelis, M. Ferenčaković, S. Rothammer, E. Kunz, M. Simčič, P. Dovč, G. Bunevski, H. Bytyqi, B. Marković, M. Brka, K. Kume, S. Stojanović, V. Nikolov, N. Zinovieva, A. A. Schönherz, B. Guldbrandtsen, M. Čačić, S. Radović, P. Miracle, C. Vernesi, I. Curik, I. Medugorac, Large-scale mitogenome sequencing reveals consecutive expansions of domestic taurine cattle and supports sporadic aurochs introgression. *Evol. Appl.* **15**, 663–678 (2022).

51. K. G. Daly, V. Mattiangeli, A. J. Hare, H. Davoudi, H. Fathi, S. B. Doost, S. Amiri, R. Khazaeli, D. Decruyenaere, J. Nokandeh, T. Richter, H. Darabi, P. Mortensen, A. Pantos, L. Yeomans, P. Bangsgaard, M. Mashkour, M. A. Zeder, D. G. Bradley, Herded and hunted goat genomes from the dawn of domestication in the Zagros Mountains. *Proc. Natl Acad. Sci. U S A* **118**, e2100901118 (2021).

52. R. Bollongino, J. Burger, A. Powell, M. Mashkour, J.-D. Vigne, M. G. Thomas, Modern taurine cattle descended from small number of Near-Eastern founders. *Molec. Biol. Evol.* **29**, 2101–2104 (2012).

53. S. Emra, M. Benz, A. B. Siddiq, V. Özkaya, Adaptions in subsistence strategy to environment changes across the Younger Dryas - Early Holocene boundary at Körtiktepe Southeastern Turkey, *Holocene* **32**, 390–413 (2022).

54. N. Pöllath, R. Garcia-Gonzalez, S. Kevork, U. Mutze, M. I. Zimmermann, M. Ozbasaran, J. Peters, A non-linear prediction model for ageing foetal and neonatal sheep reveals basic issues in early neolithic husbandry. *J. Archaeol. Sci.* **130**, 105344 (2021).

55. M. Ergun, M. Tengberg, G. Willcox, C. Douché, Plants of Aşıklı Höyük and changes through time: First archaeobotanical results from the 2010–14 excavation seasons, in *The Early Settlement at Aşıklı Höyük. Essays in Honor of Ufuk Esin*, M. Ösbaşaran, G. Duru, M. Stiner Eds. (Ege Yayınları, 2018), pp. 191–217.

56. B. Ulaş, G. Fiorentino, Recent attestations of "new" glume wheat in Turkey: a reassessment of its role in the reconstruction of Neolithic agriculture. *Veget. Hist. Archaeob.* **30**, 685–701 (2021).

57. N. Marchi, L. Winkelbach, I. Schulz, M. Brami, Z. Hofmanová, J. Blöcher, C. S. Reyna-Blanco, Y. Diekmann, A. Thiéry, A. Kapopoulou, V. Link, V. Piuz, S. Kreutzer, S. M. Figarska, E. Ganiatsou, A. Pukaj, T. J. Struck, R. N. Gutenkunst, N. Karul, F. Gerritsen, J. Pechtl, J. Peters, A. Zeeb-Lanz, E. Lenneis, M. Teschler-Nicola, S. Triantaphyllou, S. Stefanović, C. Papageorgopoulou, D. Wegmann, J. Burger, L. Excoffier, The genomic origins of the world's first farmers. *Cell* **185**, 1842–1859.e1818 (2022).

58. B. Horejs, B. Milić, F. Ostmann, U. Thanheiser, B. Weninger, A. Galik, The Aegean in the early seventh Millennium BC: Maritime networks and colonization. *J. World Prehist.* **28**, 289–330 (2015).

59. E. Özdoğan, Current research and new evidence for the neolithization process in Western Turkey. *Euro. J. Archaeol.* **18**, 33–59 (2015).

60. M. Özdoğan, in *The Central/Western Anatolian Farming Frontier*, M. Brami, B. Horejs, Eds. (Austrian Academy of Sciences, 2019), pp. 143–158.

61. R. Yaka, I. Mapelli, D. Kaptan, A. Doğu, M. Chyleński, Ö. D. Erdal, D. Koptekin, K. B. Vural, A. Bayliss, C. Mazzucato, E. Fer, S. S. Çokoğlu, V. K. Lagerholm, M. Krzewińska, C. Karamurat, H. C. Gemici, A. Sevkar, N. D. Dağtaş, G. M. Kılınç, D. Adams, A. R. Munters, E. Sağlıcan, M. Milella, E. M. J. Schotsmans, E. Yurtman, M. Çetin, S. Yorulmaz, N. E. Altınışık, A. Ghalichi, A. Juras, C. C. Bilgin, T. Günther, J. Storå, M. Jakobsson, M. de Kleijn, G. Mustafaoğlu, A. Fairbairn, J. Pearson, İ. Togan, N. Kayacan, A. Marciniak, C. S. Larsen, I. Hodder, Ç. Atakuman, M. Pilloud, E. Sürer, F. Gerritsen, R. Özbal, D. Baird, Y. S. Erdal, G. Duru, M. Özbaşaran, S. D. Haddow, C. J. Knüsel, A. Götherström, F. Özer, M. Somel, Variable kinship patterns in Neolithic Anatolia revealed by ancient genomes. *Curr. Biol.* **31**, 2455–2468 (2021).

62. H. Greenfield, A reconsideration of the secondary products revolution in south-eastern Europe: On the origins and use of domestic animals for milk, wool, and traction in the central Balkans, in *The Zooarchaeology of Fats, Oils, Milk and Dairying* (Oxbow Books, 2005), pp. 14–31.

63. C. Becker, The textile revolution. research into the origin and spread of wool production between the Near East and Central Europe. *J. Ancient Stud.* **6**, 102–151 (2016).

64. L. Excoffier, H. E. L. Lischer, Arlequin suite ver 3.5: a new series of programs to perform population genetics analyses under Linux and Windows. *Molecular Ecology Resources* **10**, 564–567 (2010).

65. S. Kumar, G. Stecher, M. Li, C. Knyaz, K. Tamura, MEGA X: Molecular evolutionary genetics analysis across computing platforms. *Mol Biol Evol* **35**, 1547–1549 (2018).

66. R. S. Waples, Temporal variation in allele frequencies: Testing the right hypothesis. *Evolution* **43**, 1236–1251 (1989).

67. E. Sandoval-Castellanos, Testing temporal changes in allele frequencies: a simulation approach. *Genet Res (Camb)* **92**, 309–320 (2010).

68. R. Bouckaert, J. Heled, D. Kühnert, T. Vaughan, C.-H. Wu, D. Xie, M. A. Suchard, A. Rambaut, A. J. Drummond, BEAST 2: A software platform for Bayesian evolutionary analysis. *PLoS Comput. Biol.* **10**, e1003537 (2014).

69. A. J. Drummond, M. A. Suchard, D. Xie, A. Rambaut, Bayesian phylogenetics with BEAUti and the BEAST 1.7. *Molec. Biol. Evol.* **29**, 1969–1973 (2012).

70. A. Rambaut, A. J. Drummond, D. Xie, G. Baele, M. A. Suchard, Posterior summarization in Bayesian phylogenetics using Tracer 1.7. *Syst. Biol.* **67**, 901–904 (2018).

71. S. Y. W. Ho, B. Shapiro, Skyline-plot methods for estimating demographic history from nucleotide sequences. *Molec. Ecol. Resour.* **11**, 423–434 (2011).

72. J. Heled, A. J. Drummond, Bayesian inference of population size history from multiple loci. *BMC Evol. Biol.* **8**, 289 (2008).

73. M. A. Beaumont, Approximate Bayesian Computation in Evolution and Ecology. *Ann. Rev. Ecol. Evol. Syst.* **41**, 379–406 (2010).

74. N. Rohland, D. Reich, S. Mallick, M. Meyer, R. E. Green, N. J. Georgiadis, A. L. Roca, M. Hofreiter, Genomic DNA Sequences from Mastodon and Woolly Mammoth Reveal Deep Speciation of Forest and Savanna Elephants. *PLoS Biol.* **8**, e1000564 (2010).

75. N. Rohland, M. Hofreiter, Comparison and optimization of ancient DNA extraction. *Biotechniques* **42**, 343-352 (2007).

76. J. Dabney, M. Knapp, I. Glocke, M.-T. Gansauge, A. Weihmann, B. Nickel, C. Valdiosera, N. García, S. Pääbo, J.-L. Arsuaga, M. Meyer, Complete mitochondrial genome sequence of a Middle Pleistocene cave bear reconstructed from ultrashort DNA fragments. *Proc. Natl Acad. Sci. U S A* **110**, 15758–15763 (2013).

77. P. Korlević, T. Gerber, M.-T. Gansauge, M. Hajdinjak, S. Nagel, A. Aximu-Petri, M. Meyer, Reducing microbial and human contamination in DNA extractions from ancient bones and teeth. *Biotechniques* **59**, 87–93 (2015).

78. K. G. Daly, P. M. Delser, V. E. Mullin, A. Scheu, V. Mattiangeli, M. D. Teasdale, A. J. Hare, J. Burger, M. P. Verdugo, M. J. Collins, R. Kehati, C. M. Erek, G. Bar-Oz, F. Pompanon, T. Cumer, C. Çakırlar, A. F. Mohaseb, D. Decruyenaere, H. Davoudi, Ö. Çevik, G. Rollefson, J.-D. Vigne, R. Khazaeli, H. Fathi, S. B. Doost, R. R. Sorkhani, A. A. Vahdati, E. W. Sauer, H. A. Kharanaghi, S. Maziar, B. Gasparian, R. Pinhasi, L. Martin, D. Orton, B. S. Arbuckle, N. Benecke, A. Manica, L. K. Horwitz, M. Mashkour, D. G. Bradley, Ancient goat genomes reveal mosaic domestication in the Fertile Crescent. *Science* **361**, 85–88 (2018).

79. M. Meyer, M. Kircher, M.-T. Gansauge, H. Li, F. Racimo, S. Mallick, J. G. Schraiber, F. Jay, K. Prüfer, C. de Filippo, P. H. Sudmant, C. Alkan, Q. Fu, R. Do, N. Rohland, A. Tandon, M. Siebauer, R. E. Green, K. Bryc, A. W. Briggs, U. Stenzel, J. Dabney, J. Shendure, J. Kitzman, M. F. Hammer, M. V. Shunkov, A. P. Derevianko, N. Patterson, A. M. Andrés, E. E. Eichler, M. Slatkin, D. Reich, J. Kelso, S. Pääbo, A high-coverage genome sequence from an archaic Denisovan individual. *Science* **338**, 222-226 (2012).

80. C. Gamba, E. R. Jones, M. D. Teasdale, R. L. McLaughlin, G. Gonzalez-Fortes, V. Mattiangeli, L. Domboróczki, I. Kővári, I. Pap, A. Anders, A. Whittle, J. Dani, P. Raczky, T. F. G. Higham, M. Hofreiter, D. G. Bradley, R. Pinhasi, Genome flux and stasis in a five millennium transect of European prehistory. *Nat. Commun.* **5**, 5257 (2014).

81. M.-T. Gansauge, M. Meyer, Single-stranded DNA library preparation for the sequencing of ancient or damaged DNA. *Nat. Protocols* **8**, 737–748 (2013).

82. H. Li, R. Durbin, Fast and accurate long-read alignment with Burrows–Wheeler transform. *Bioinformatics* **26**, 589-595 (2010).

83. D. C. Koboldt, Q. Zhang, D. E. Larson, D. Shen, M. D. McLellan, L. Lin, C. A. Miller, E. R. Mardis, L. Ding, R. K. Wilson, VarScan 2: Somatic mutation and copy number alteration discovery in cancer by exome sequencing. *Genome Res.* **22**, 568-576 (2012).

84. J. Hein, M. Schierup, C. Wiuf, *Gene Genealogies, Variation and Evolution. A Primer in Coalescent Theory* (Oxford Univ. Press, 2004), vol. 54.

85. G. Bertorelle, A. Benazzo, S. Mona, ABC as a flexible framework to estimate demography over space and time: some cons, many pros. *Mol. Ecol.* **19**, 2609-2625 (2010).

86. C. P. Robert, J. M. Cornuet, J. M. Marin, N. S. Pillai, Lack of confidence in approximate Bayesian computation model choice. *Proc. Natl Acad. Sci. U S A* **108**, 15112-15117 (2011).

87. P. Pudlo, J.-M. Marin, A. Estoup, J.-M. Cornuet, M. Gautier, C. P. Robert, Reliable ABC model choice via random forests. *Bioinformatics* **32**, 859-866 (2016).

88. E. Sandoval-Castellanos, E. Palkopoulou, L. Dalen, Back to BaySICS: A user-friendly program for Bayesian Statistical Inference from Coalescent Simulations. *PLoS One* **9**, e98011 (2014).

89. R. Krauß, Beginnings of the Neolithic in Southeast Europe: the Early Neolithic sequence and absolute dates from Džuljunica-Smărdeš (Bulgaria). *Docum. Praehist.* **41**, 51-77 (2014).

90. D. De Groene, Pigs and humans in Early Neolithic south-eastern Europe. *Docum. Praehist.* **45**, 38-51 (2018).

91. M. Mashkour, R. Khazaeli, H. Fathi, S. Amiri, D. Decruyenaere, A. Mohaseb, H. Davoudi, S. Sheikhi, E. W. Sauer, in *Sasanian Persia: Between Rome and the Steppes of Eurasia*, E. Sauer, Ed. (Edinburgh Univ. Press, 2017), pp. 74-96.

92. K. Daly, B. S. Arbuckle, C. Rossi, V. Mattiangeli, P. A. Lawlor, M. Mashkour, E. Sauer, J. Lesur, L. Atici, C. M. Erek, D. G. Bradley, A novel lineage of the *Capra* genus discovered in the Taurus Mountains of Turkey using ancient genomics. *eLife* **11**, e82984 (2022).

93. J. Oldenstein, *KASTELL ALZEY Archäologische Untersuchungen im Spätrömischen Lager und Studien zur Grenzverteidigung im Mainzer Dukat* (Univ. of Mainz, 2009), vol. **1**.

94. B. Boulestin, Mass cannibalism in the linear pottery culture at Herxheim (Palatinate, Germany). *Antiquity* **83**, 968–982 (2009).

95. N. Card, M. Edmonds, A. Mitchell, *The Ness of Brodgar, as it Stands*. M. Edmonds, A. Mitchell, Eds. (The Orcadian, 2020).

96. N. Card, To cut a long story short: Formal chronological modelling for the Late Neolithic site of Ness of Brodgar Orkney, *Europ. J. Archaeol.* **21**, 217–263 (2018).

97. D. Griffiths, J. Harrison, M. Athanson, *Beside the Ocean: Coastal Landscapes at the Bay of Skaill, Marwick, and Birsay Bay, Orkney: Archaeological Research 2003-18*. (Oxbow Books, 2019), p. 346.

98. N. M. Sharples, in *From Machair to Mountains: Archaeological Survey and Excavation in South Uist*, M. Parker Pearson, Ed. (Oxbow Books, 2012), vol. **4**, pp. 215–258.

99. M. P. Pearson, J. Mulville, H. Smith, P. Marshall, Cladh Hallan: Roundhouses and the dead in the Hebridean Bronze Age and Iron Age, Part I: Stratigraphy, spatial organisation and chronology, in *Sheffield Environmental and Archaeological Research Campaign in the Hebrides*, (Oxbow Books, 2021), vol. **8**, p. 568.

100. N. Sharples, *A Late Iron Age Farmstead in the Outer Hebrides. Excavations at Mound 1, Bornais, South Uist*. (Oxbow Books, 2012), p. 419.

101. M. P. Pearson, M. Brennand, J. Mulville, H. Smith, Cille Pheadair: A norse farmstead and pictish burial cairn in South Uist, in *Sheffield Environmental and Archaeological Research Campaign in the Hebrides*, (Oxbow Books, 2018), vol. **7**, p. 656.

102. A. J. Lawson, M. J. Allen, *Potterne 1982-5: Animal Husbandry in Later Prehistoric Wiltshire*. (Trust for Wessex Archaeology, 2000).

103. B. W. Cunliffe, *Danebury: Anatomy of an Iron Age Hillfort*. (Batsford, 1983).

104. P. Wallace, The archaeology of Viking Dublin, in *The comparative history of urban origins in non-Roman Europe*, H. B. Clarke, A. Simms, Eds. (Oxford 1985), BAR International Series, 255(1), pp. 103–145.

105. S. Stoddart, A. Bonanno, T. Gouder, C. Malone, D. Trump, Cult in an island society: Prehistoric Malta in the Tarxien period. *Cambridge Archaeological J.* **3**, 3–19 (1993).

106. H. Greenfield, T. Jongsma, in *Space and Spatial Analysis in Archaeology*, E. C. Robertson, J. D. Siebert, D. C. Fernandez, M. U. Zender, Eds. (Univ. of Calgary Press, 2006), chap. 8, pp. 69–79.

107. A. Whittle, L. Bartosiewicz, D. Borić, P. Pettitt, M. P. Richards, In the beginning : new radiocarbon dates for the Early Neolithic in northern Serbia and south-east Hungary. *Antaeus.* **25**, 63-118 (2002).

108. M. Porčić, T. Blagojević, J. Pendić, S. Stefanović, The Neolithic demographic transition in the Central Balkans: Population dynamics reconstruction based on new radiocarbon evidence. *Philosop. Trans. R. Soc. B: Biol. Sci.* **376**, 20190712 (2021).

109. A. Bulatović, D. Milanović, *Bubanj The Eneolithic and the Early Bronze Age Tell in Southeastern Serbia*. (Verlag der Österreichischen Akademie der Wissenschaften, 2020), vol. **90**, p. 418.

110. T. Dothan, A. Zukerman, A preliminary study of the Mycenaean IIIC:1 pottery assemblages from Tel Miqne-Ekron and Ashdod. *Bullet. Am. Schools Oriental Res.* **333**, 1 (2004).

111. L. K. Horwitz, E. Dahan. Animal husbandry practices during the historic periods, in *Yoqne'am I. The Late Periods*, A. Ben-Tor, M. Avissar, Y. Portugali, Eds. (Qedem Reports 3, 1996), pp. 246–255.

112. T. E. Levy, D. Alon, *Shiqmim I: Studies Concerning Chalcolithic Societies in the Northern Negev Desert, Israel (1982-1984)* (B.A.R., 1987).

113. B. Horejs, C. Schwall. Interaction as a stimulus?: Çukuriçi Höyük and the transition from the Late Chalcolithic to the Early Bronze Age in Western Anatolia, in *Communities in Transition: The Circum-Aegean Area During the 5th and 4th Millennia BC*, S. Dietz, F. Mavridis, Ž. Tankosić, T. Takaoğlu, Eds. (Oxbow Books, 2018), pp. 530–537.

114. B. S. Arbuckle, V. Özkaya, Animal exploitation at Körtik Tepe : An early Aceramic Neolithic site in southeastern Turkey. *Paléorient* **32**, 113-136 (2006).

115. V. Özkaya, A. Coşkun, Körtik Tepe, in *The Neolithic in Turkey. New Excavations and New Research. The Tigris Basin*, M. Özdoğan, N. Başgelen, I. Kuniholm, Eds. (Archaeology and Art Publications, 2011), pp. 89–127.

116. M. Benz, A. Coşkun, C. Rössner, K. Deckers, S. Riehl, K. W. Alt, V. Özkaya, First evidence of an Epipalaeolithic hunter-fisher-gatherer settlement at Körtik Tepe. *Arkeometri Sonuçları Toplantısı* **28**, 65-78 (2013).

117. B. S. Arbuckle, Revisiting Neolithic caprine exploitation at Suberde, Turkey, *J. Field Archaeol.* **33**, 219–236 (2008).
